# Tailoring Cobalt(II) Schiff Base Photocatalysts for Enhanced LED-Induced Free Radical Polymerization

**DOI:** 10.1021/acspolymersau.5c00112

**Published:** 2025-11-10

**Authors:** Larissa F. Oliveira, Naralyne M. Pesqueira, Yasmin M. Shimizo, Maria L. B. Figueiredo, Valdemiro P. Carvalho-Jr, Beatriz E. Goi

**Affiliations:** School of Technology and Sciences, 28108 São Paulo State University (Unesp), Presidente Prudente, São Paulo 19060-900, Brazil

**Keywords:** cobalt complex, schiff base, photocatalyst, LED irradiation, photopolymerization

## Abstract

Co­(II) complexes, despite their potential as cost-effective alternatives to noble-metal systems, remain underexplored as photocatalysts (PCs) in free radical photopolymerization (FRP). In this study, a series of Co­(II) complexes bearing symmetrical Schiff bases (**Co**–**Ph**, **Co**–**EtO**, **Co–Cl**, **Co**–**Me**, and **Co**–*t* **Bu**) was synthesized and characterized by FTIR, UV–vis, \ fluorescence spectroscopy, MALDI-TOF mass spectrometry, cyclic voltammetry, and advanced density functional theory (DFT/TD-DFT) calculations. Their photocatalytic performance was evaluated in three-component photoinitiating systems with ethyl 4-(dimethylamino)­benzoate (EDB) and diphenyliodonium hexafluorophosphate (Iod) for the FRP of trimethylolpropane ethoxylate triacrylate (TMPETA) under UV, violet, and blue LED irradiation. Co­(II) complexes enabled efficient polymerization under optimized conditions, reaching high conversions without an inhibition period under UV irradiation. **Co–EtO** demonstrated a superior photocatalytic efficiency across all tested wavelengths relative to that of the other Co­(II) complexes evaluated in this study. This enhanced performance is attributed to a synergistic combination of its unique structural, electronic, and electrochemical properties. The proposed mechanism was supported by photolysis experiments, literature data, and free energy calculations, indicating the involvement of both oxidative and reductive pathways.

## Graphical abstract

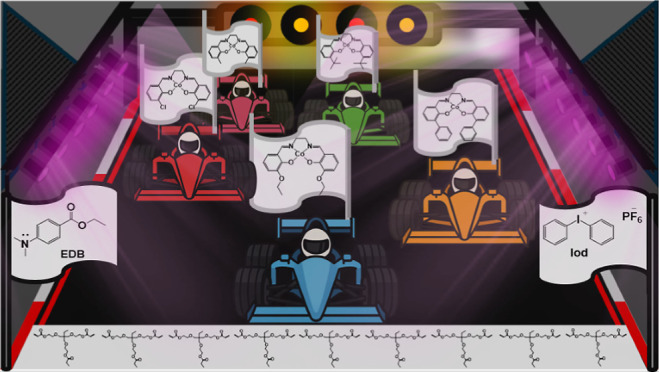

## 1 Introduction

Visible light has emerged as a powerful tool in polymerization reactions, driven by the increasing demand for sustainable and energy-efficient methodologies. Nevertheless, the development of innovative processes remains a significant challenge. Photochemistry enables transformations under mild conditions, reduced temperatures, and lower costs.^1–4^ As the demand for sustainable and energy-efficient strategies increases, photopolymerization has gained prominence in both academic and industrial applications, offering rapid monomer conversion and reduced environmental impact.^1,3,4,6,7^ Compared to thermal methods, this advantage is particularly relevant for acrylates and methacrylates, which typically require elevated temperatures to initiate polymerization.^1,5^

The free radical photopolymerization (FRP) plays a key role in polymer manufacturing, offering broad applicability and high process efficiency.^8,9^ Building upon the advantages of light-driven methods, FRP has advanced rapidly in recent decades, leading to diverse applications, such as paints,^10^ coatings,^11^ varnishes,^12^ microelectronics,^13^ adhesives,^14^ dentistry,^15^ curing technologies^16–18^ and 3D printing,^2,9,19^ owing to its speed, high efficiency, and environmentally friendly conditions.^20,21^ In this process, photoinitiators (PIs) absorb light and generate reactive species that initiate polymerization. The development of redox photocatalysts has enhanced the FRP efficiency by enabling catalytic cycle regeneration.^7,22^ In addition, transition-metal complexes have emerged as photocatalysts due to their electron-donating capabilities, broad visible-light absorption range, and redox potentials tailored to efficiently interact with various additives such as iodonium salts and amines.^22–24^

Ruthenium^25–27^ and iridium^3,22,28^ complexes have been established as the most extensively explored systems in this field of FRP. Despite their high efficiency, these noble metals present limitations related to scarcity, cost, and potential toxicity concerns.^7,28–30^ To overcome these issues, attention has increasingly turned to 3d metal complexes. Copper and iron complexes have been widely explored as photocatalysts in FRP, achieving high monomer conversions under irradiation across a broad spectral range, including UV, violet, blue, and green light.^24,30^ Among 3d metals, cobalt complexes offer a cost-effective and environmentally friendly alternative to noble metal systems.^31–33^ Schiff base–cobalt­(II) complexes, especially those featuring tetradentate N_2_O_2_ coordination frameworks, have emerged as valuable platforms in the development of homogeneous catalytic systems due to their structural adaptability and functional efficiency.^34–36^ Over the years, Schiff bases are widely studied in coordination chemistry for their easy synthesis, structural versatility, and ability to form stable metal complexes.^37^

Our research group has previously investigated photopolymerization reactions, such as organometallic-mediated radical polymerization (OMRP),^35,38–42^ atom-transfer radical polymerization (ATRP),^43–48^ and FRP.^24,29,47^ The aim was to deepen the understanding of these photopolymerization reactions using complexes employing metals as cobalt-, nickel-, iron-, manganese-, and copper-bearing Schiff bases,^29,35,40,43,48,51^ NHCs,^43,47,52^ or pyridine-benzothiazole ligands.^24^ The polymers obtained from the trimethylolpropane ethoxylate triacrylate (TMPETA) monomer via FRP reactions catalyzed by copper, nickel, and manganese complexes have been successfully applied in 3D printing, producing well-defined structures with smooth surfaces and precise spatial control using the direct laser writing technique.^24,47,52^

To further expand the application of first-row transition-metal complexes as photocatalysts in photopolymerization reactions, five Co­(II) complexes bearing Schiff base ligands were evaluated for FRP of TMPETA under UV, violet, and blue light. These complexes were successfully synthesized and exhibited effective photocatalytic activity in combination with EDB and Iod as additives (Scheme 1). Photopolymerization experiments were conducted using LED irradiation at 365, 390–405, or 420 nm, with varying weight percentages of photocatalysts and additives. The reaction mechanism was proposed based on spectroscopic analyses, theoretical calculations, and literature data.

**Scheme 1:**
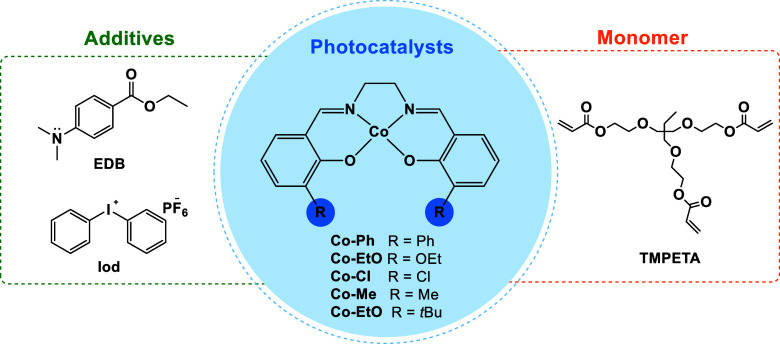
Structures of Photocatalysts, Additives, and Monomers Used in FRP Reactions

## 2 Experimental Section

### 2.1 General Remarks

All reactions and manipulations were performed under an argon atmosphere using standard Schlenk techniques. Ethylenediamine, 3-ethoxy-salicylaldehyde, sodium hydroxide, CoCl_2_ hexahydrate, methanol (MeOH, 99.8%, P.A.), dichloromethane (CH_2_Cl_2,_ 99.5%, P.A.), dimethylformamide (DMF, 99.8%, P.A.), and tetrahydrofuran (THF, 99.0%, P.A.) were purchased from Sigma-Aldrich. CoCl_2_ was carefully dried in a reaction flask under reduced pressure (0.5 Torr) using a hair dryer until its color changed from purple to blue. Diphenyl iodonium hexafluorophosphate (Iod) and ethyl 4-(dimethylamino)-benzoate (EDB) were purchased from Sigma-Aldrich. Trimethylolpropane ethoxylate triacrylate (TMPETA) was acquired from Sigma-Aldrich and used as an acrylic monomer for the radical photopolymerization. The functionalized salicylaldehydes (3-phenyl-salicylaldehyde, 3-chloro-2-hydroxybenzaldehyde, 3-methyl-salicylaldehyde, and 3-*tert*-butyl-salicylaldehyde) were obtained according to the literature.^53–55^ The Salen-type ligands (Salen-Ph, Salen-EtO, Salen-Cl, Salen-Me, and Salen-*t*-Bu) and their respective complexes (**Co–EtO**, **Co–Cl**, **Co–Me**, and **Co–** *t* **Bu**) were obtained following procedures described in the literature.^38,40,71,72^ The characterization data for the ligands and their corresponding complexes are provided in the Supporting Information (Figures S1–S20).

### 2.2 Analysis

FTIR spectra were recorded using a PerkinElmer Frontier spectrometer equipped with a diamond attenuated total reflectance (ATR) accessory. Measurements were taken across the spectral range of 4000–250 cm^–1^, maintaining a resolution of 2 cm^–1^ at room temperature (298 K). Elemental analysis was determined via a PerkinElmer CHN 2400 elemental analyzer. ^1^H NMR spectra were obtained in deuterated chloroform (CDCl_3_) at 298 K by using an Agilent MR 400 Ultrashield spectrometer operating at a frequency of 400.13 MHz for proton analysis. Chemical shifts (δ) are presented in parts per million (ppm) relative to tetramethylsilane (TMS), which was used as an internal reference standard. MALDI-TOF mass spectra were acquired using a Bruker Autoflex Max spectrometer operating in the positive ion reflector mode. Samples were prepared by thoroughly mixing the analyte with the matrix α-cyano-4-hydroxycinnamic acid (HCCA) dissolved in dichloromethane (CH_2_Cl_2_), followed by deposition onto the target plate and subsequent air drying prior to analysis. Electronic absorption spectra were recorded on a Shimadzu UV-2600 UV–vis spectrophotometer utilizing quartz cuvettes with a 1 cm path length. The analyses were performed on CH_2_Cl_2_ solutions of the complexes at a concentration of 1 × 10^–5^ mol L^–1^. All DFT calculations in this work were conducted using the ORCA 5.0 quantum chemistry package.^56^ A computational study was carried out to evaluate the doublet and quartet possible spin multiplicities for the Co­(II)–Schiff base complexes gas-phase geometry optimizations were carried out using the unrestricted DFT method, when applicable. The long-range-corrected (LC) hybrid density ωB97X-D3­(BJ) functional^57,58^ was employed for both optimization and single-point energy calculations, with a def2-SVP basis set applied to C, H, N, and O atoms and def2-TZVP for Co.^59^ Dispersion interactions were accounted for using the Grimme’s D3 correction^60^ with the Becke–Johnson (BJ) damping function.^61^ To reduce computational cost, the resolution-of-the-identity approximation for Coulomb integrals (RI-J) combined with the chain-of-sphere-exchange (COSX) method (RIJCOSX)^62,63^ was applied, utilizing the def2/J auxiliary basis sets. All calculations were performed with tight convergence criteria. Harmonic vibrational frequencies were computed at this level of theory to ensure that the stationary points were true minima, signifying equilibrium structures on the potential energy surfaces. This further ensured that imaginary frequencies were not generated in the minimum structures. Selected bond distances and bond angles from these calculations are listed in Table S1. Among the tested spin states, the quartet state (three unpaired electrons) yielded the lowest energy values: −2570.317521, −2717.393488, −2335.309566, −2563.569336, and −3174.774204 Hartree for **Co–** *t* **Bu**, **Co–Ph**, **Co–Me**, **Co–EtO**, and **Co–Cl**, respectively. The corresponding energies for the doublet state were −2570.303263, −2717.379948, −2335.292411, −2563.553809, and −3174.755416 Hartree, which are 8.9, 8.4, 10.7, 8.7, and 11.8 kcal mol^–1^ higher than the respective quartet states. Based on these results, all subsequent calculations were carried out using the quartet state. To theoretically predict the UV–vis spectra of all complexes, TD-DFT calculations were performed at the ωB97X-D3­(BJ)/def2-SVP [Co: def2-TZVP] level.^64^ This computational approach was chosen because its long-range-separated functional provides a more balanced treatment of charge-transfer excitations, making it particularly suitable for describing electron excitations,^65,66^ including those of organometallic systems.^67,68^ Natural transition orbitals (NTOs)^69^ were generated as cube files using the ORCA program and visualized with GaussView 6.^70^ Electrochemical measurements were conducted via cyclic voltammetry using an Autolab PGSTAT204 potentiostat, at 25 °C, in DMF solution with 0.1 M of tetrabutylammonium hexafluorophosphate (*n*-Bu_4_NPF_6_) under an argon atmosphere. The voltammetric cell consisted of three electrodes: stationary platinum disk (working electrode), platinum wire (auxiliary electrode), and Ag/AgCl (reference electrode). The FRP of TMPETA was initiated under LED irradiation at 365 and 390–405 nm, each delivering an intensity of 10 mW/cm^2^ at the sample position. The polymerization process was monitored in situ by using a PerkinElmer Frontier FTIR spectrometer. Monomer conversion was determined by quantifying the residual monomer content via infrared spectroscopy. Thermogravimetric and differential thermal analyses (TGA/DTA) of the polymers were performed using a Netzsch STA 449 F3 simultaneous analyzer under a dry air atmosphere in the temperature range of 30–800 °C.

### 2.3 Synthesis of **Co–Ph**

**Co–Ph** was synthesized using modified procedures reported in the literature.^38^ A methanolic solution of NaOH (0.160 g, 4.0 mmol) was added to a round-bottom flask containing the Salen-Ph (0.840 g, 2.0 mmol). The mixture was stirred at room temperature for 2 h. Afterward, it was degassed under an Ar atmosphere and transferred to a Schlenk flask connected to a reflux system containing anhydrous CoCl_2_ (0.260 g, 2.0 mmol). The reaction mixture was maintained under reflux for 24 h. The reaction mixture was reduced under a vacuum, and the resulting precipitate was filtered and recrystallized in diethyl ether. Yield: 91% (860 mg). FTIR (cm^–1^): 3088–2932 ν­(aromatic C–H), 1609 ν­(C=N), 1228 ν­(C–O), 520 ν­(Co–N), 480 ν­(Co–O); UV–Vis (CH_2_Cl_2_, nm (mol L^–1^ cm^–1^)): λ_max_ (ε) = 237 (63,000), 362 (23,000), 423 (19,000), 497 (5000); MALDI-TOF: *m*/*z* calcd for C_28_H_22_CoN_2_O_2_, 477.1000 g mol^–1^; found, 477.9822 [M + H]^+^; Elemental analysis Calcd (%) for C_28_H_22_CoN_2_O_2_: C, 70.44; H, 4.64; N, 5.87%. Found: C, 70.68; H, 4.84; N, 5.93%.

### 2.4 FRP Procedure

For the FRP of TMPETA, photoreactive formulations were prepared using a three-component system composed of PC, Iod, and EDB, following a laminate procedure.^29^ The photopolymerization process was initiated by LED irradiation at 365, 390, 405, or 420 nm, with an incident light intensity of 10 mW/cm^2^ at the sample surface. Monomer conversion was monitored by FTIR through the decrease of the characteristic C=C stretching band at 1635 cm^–1^ during 1200 s.

## 3 Results and Discussion

Five symmetrical Schiff-base-Co­(II) complexes, **Co**–**Ph**, **Co**–**EtO**, **Co–Cl**, **Co**–**Me**, and **Co**–*t* **Bu**, were obtained through an equimolar reaction between anhydrous CoCl_2_ and the respective deprotonated Salen-type ligands in a methanolic NaOH solution.^38–40^ These complexes were characterized by FTIR, UV–vis, fluorescence spectroscopy, MALDI-TOF mass spectrometry, cyclic voltammetry, and computational studies.

The FTIR spectra of **Co–Ph** and its corresponding Salen-Ph ligand exhibit characteristic absorption bands in the 250–4000 cm^–1^ region (Figure S6). As expected, the vibrational stretching band of ν­(O–H), observed at 2605 cm^–1^ in the spectrum of the Salen-Ph ligand, is absent in the complex, indicating the coordination of the hydroxyl oxygen atoms to the cobalt center. The coordination of iminic nitrogens to the metal center was evidenced by the shift of the ν­(C=N) vibrational stretching to a lower wavenumber, on the order of 74 cm^–1^, occurring at 1683 cm^–1^ in the FTIR spectrum of the Salen-Ph ligand, while **Co–Ph** exhibited this band at 1609 cm^–1^. Furthermore, the ν­(C–O) stretching band of the ligand, detected at 1080 cm^–1^, is displaced to 1228 cm^–1^ upon complexation, indicating the involvement of the phenolic oxygen in metal coordination. These spectral changes confirm the formation of the Co­(II)–Schiff base complexes and the tetradentate coordination mode of the ligands. Bands around 520 and 480 cm^–1^ were observed in the FTIR spectrum of the **Co–Ph**, attributed to ν­(Co–N) and ν­(Co–O) vibrational transitions, respectively.^73,74^

The MALDI-TOF mass spectrum of **Co–Ph** showed a molecular ion peak at *m*/*z* 477.9822 (Figure S11), corresponding to the expected complex with a theoretical value of 477.1100 *m*/*z*.

The optimized geometries of the Co­(II) complexes were optimized by using DFT calculations (Figure S21). The Schiff base ligands coordinate the Co­(II) center in an (N,O,N,O)-tetradentate coordination mode. In all the five complexes, the Co–N and Co–O bond lengths are similar, indicating comparable coordination environments. Specifically, for **Co–Ph**, the bond lengths are 2.040 and 2.038 Å for N(1)–Co and N(2)–Co, respectively, and 1.922 and 1.923 Å for O(1)–Co and O(2)–Co. For **Co–EtO**, the Co–N and Co–O bond lengths are slightly shorter: 2.028 Å for N(1), 2.030 Å for N(2), 1.913 Å for O(1), and 1.911 Å for O(2). In turn, for **Co–Cl**, the Co–N bonds are slightly longer: 2.065 Å for N(1) and 2.058 Å for N(2), with Co–O distances of 1.923 Å for O(1) and 1.924 Å for O(2). For both **Co–Me** and **Co–** *t* **Bu**, the bond lengths are similar to those of **Co–Ph**. Specifically, for **Co–Me**: 2.029 Å for N(1), 2.033 Å for N(2), 1.920 Å for O(1), and 1.919 Å for O(2); for **Co–** *t* **Bu**: 2.036 Å for N(1), 2.039 Å for N(2), 1.921 Å for O(1), and 1.924 Å for O(2). The bond angles in the Co­(II) complexes illustrate their slightly distortions. The N(1)–Co–O(1) angle ranges from 88.92° to 92.14° and the N(2)–Co–O(2) angle ranges from 89.46° to 92.08°, with **Co**–**Ph** and **Co**–**Me** showing the lower and the highest symmetry among them, respectively. For **Co**–**Ph** and **Co**–*t* **Bu**, the O(1)–Co–O(2) angles are shorter than that obtained for the other complexes, indicating a distorted tetrahedral geometry, while **Co–EtO**, **Co–Cl**, and **Co**–**Me** presents a geometry closer to a tetrahedron.

This structural interpretation is further supported by the geometric parameter τ_4_ ^76^ calculated using the formula $\tau_{4}=360-\frac{\left(\alpha +\beta \right)}{141}$ , where α and β are the two largest bond angles around the metal center. The τ_4_ values range from 1.00 for a perfect tetrahedral geometry to zero for an ideal square planar geometry. For **Co**–**Ph** and **Co**–*t* **Bu** τ_4_ are 1.19 and 1.16, respectively, indicating a distorted tetrahedral geometry, while τ_4_ are 1.11, 1.11, and 1.10 for **Co**–**EtO**, **Co–Cl**, and **Co**–**Me**, respectively, indicating a more regular tetrahedral geometry. It is important to highlight that all bond lengths and angles around the Co­(II) center are consistent with those typically observed in similar coordination compounds.^38^

The UV–vis spectra of the Co­(II) complexes and their respective Schiff base ligands were obtained in a CH_2_Cl_2_ solution at room temperature (Figures S16–S20). To better understand their absorption properties, the electronic structure of the Co­(II) complexes was computationally investigated. For these investigations, time-dependent density functional theory (TD-DFT) calculations were used. Both simulated and experimental spectra were collected across the 200–800 nm range (as shown in Figures S22–S26). The absorption maxima and their theoretical assignments are summarized in Tables S2–S6. The main transitions are illustrated using natural transition orbitals (NTOs).^43^ The absorption spectra of Salen-type ligands exhibited bands with maxima in the region of 215 to 280 nm and 290 to 350 nm, which are attributed to the π → π* and *n* → π* transitions corresponding to the benzene ring and the imine group, respectively. On the other hand, in the absorption spectra of the Co­(II) complexes, the benzene ring transitions are hypsochromically shifted and the imine group transitions are bathochromically shifted. The Co­(II) complexes also exhibited bands in the regions of 330 to 445 nm and 450 to 550 nm, corresponding to metal-to-ligand charge transfer (MLCT) and d–d transitions, respectively.^41^ The experimental data have a good agreement with the quartet ground-state calculations of the complexes. The Co­(II) complexes exhibited significant absorption in the 300–500 nm range, which makes them suitable for use as photocatalysts under UV and visible light irradiations. The molar extinction coefficients (ε) at their respective absorption maxima, along with their absorption at the wavelengths corresponding to the LEDs employed in the photopolymerization experiments (365, 405, and 420 nm), are presented in Figure 1 and summarized in Table S7. **Co**–**EtO** and **Co**–**Ph** exhibit the highest molar extinction coefficients at 365, 390–405, and 420 nm, followed by those of **Co–Cl**. Meanwhile, **Co**–*t* **Bu** and **Co–Me** showed the lowest molar extinction coefficients at the mentioned wavelengths (Table S7).

**Figure 1:**
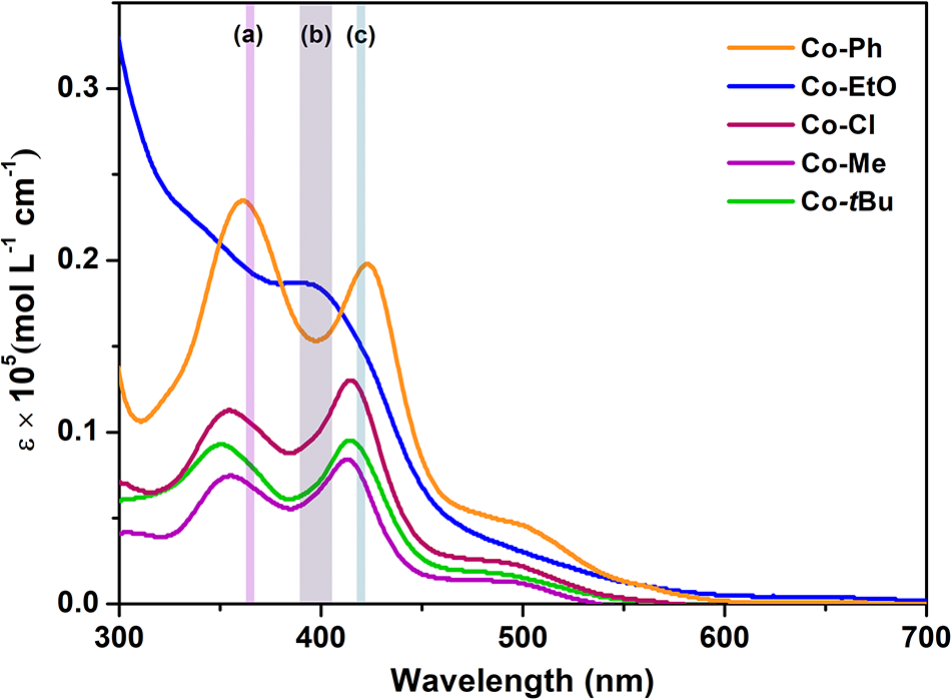
UV–vis spectra of the complexes **Co**–**Ph**, **Co**–**EtO**, **Co–Cl**, **Co**–**Me**, and **Co**–*t* **Bu** in CH_2_Cl_2_ ([Co] = 1 × 10^–5^ mol L^–1^) at 25 °C; (a) LED@365 nm, (b) LED@390–405 nm, and (c) LED@420 nm.

The emission properties of the Co­(II) complexes were investigated in CH_2_Cl_2_ at 25 °C upon excitation at 365 nm (Figure S27). **Co–EtO** and **Co–** *t* **Bu** exhibited similar profiles, with maximum emission bands centered at 400 nm. Notably, **Co–Cl** displays an emission band at 460 nm, exhibiting fluorescence in the green light spectrum. In contrast, **Co**–**Ph** and **Co**–**Me** were nonemissive, which may be attributed to nonradiative relaxation pathways involving the rapid deactivation of the excited state.^29,51^ Despite their strong absorption in the UV–visible region, 3d metal complexes with similar Schiff base ligands have been reported to exhibit weak or no emission, attributed to the limited orbital overlap between the metal center and the ligands.^29,49^

Electrochemical properties of the Co­(II) complexes were investigated by cyclic voltammetry to elucidate their redox behavior, which is critical for understanding their role in photocatalysis (Figures S28 and S29) The half-wave potentials (*E*_1/2_) for the Co­(II)/Co­(III) redox processes are summarized in Table S8, while the Co­(I)/Co­(II) redox processes are presented in Table S9.^38,73,77,78^ As the electron-donating ability of the substituents in the complexes increases, following the order: **Co–Cl** < **Co–Ph** < **Co–Me** < **Co–** *t* **Bu** < **Co–EtO**.^38,73,77,78^ Notably, **Co–EtO** exhibited the most favorable Co­(II)/Co­(III) oxidation potential at −0.24 V (vs Ag/AgCl) and a Co­(II)/Co­(I) oxidation potential at −1.03 V (vs Ag/AgCl), indicating its enhanced ability to participate in both oxidative and reductive electron-transfer reactions. In contrast, **Co–Cl** showed a Co­(II)/Co­(III) oxidation potential of 0.38 V and a Co­(II)/Co­(I) oxidation potential at −1.07 V (vs Ag/AgCl), highlighting the influence of the chloro substituent. These variations in redox potentials across the series of complexes are directly influenced by the electronic nature of the Schiff base ligands, with electron-donating groups generally shifting potentials to more negative values, facilitating oxidation, and electron-withdrawing groups making oxidation more difficult. Furthermore, the electrochemical properties of the photocatalysts are a key parameter used to calculate the free energy change of electron transfer (Δ*G*_et_) between the PC and Iod or EDB.^29^ A thermodynamic analysis, comparing the redox potentials of the Co­(II) complexes with those of the electron donor (EDB, *E*_ox_ = 1.00 V vs SCE) and electron acceptor (Iod, *E*_red_ = −0.20 V vs SCE), confirms the feasibility of both oxidative and reductive pathways in the proposed photocatalytic cycle. For instance, the oxidation potentials of all Co­(II) complexes are sufficiently negative to be oxidized by Iod, while their reduction potentials are positive enough to be reduced by EDB. The free energy changes (Δ*G*_et_) for the PC/Iod and PC/EDB interactions, as detailed in Table S10, further support the thermodynamic driving force for these electron-transfer processes, with **Co–EtO** showing the most negative Δ*G*_et_ values, consistent with its superior photocatalytic performance.

### 3.1 Free Radical Photopolymerization

The FRP of TMPETA was carried out at 25 °C using five Co­(II) complexes, **Co–Ph**, **Co–EtO**, **Co–Cl**, **Co–Me**, and **Co–** *t* **Bu**, as photocatalysts, in the presence of Iod and EDB as additives, under LED irradiation at 365, 390–405, or 420 nm. Previous results demonstrated that the PC/Iod/EDB system promotes efficient polymerization, enhancing TMPETA conversion and enabling activity under longer wavelength irradiation.^24,29,49,52^ Thus, photopolymerization experiments were performed with different amounts of Co^II^/Iod/EDB, relative to the monomer weight (Tables 1 and 2, Figures 2, 3, and S30–S34), using a lamination procedure to minimize inner filter effects.^24^ The formulations were irradiated directly in the FTIR sample compartment without prior deoxygenation.

**Table 1:** Final Conversions of TMPETA Achieved in the Laminate under LED Irradiation Using 0.1 w % of PC and Different Amounts of Additives

entry	PC	Co^II^/Iod/EDB (w %/w %/w %)	conv. (%) at 365 nm LED	conv. (%) at 390–405 nm LED
1	**Co–Ph**	0.1/1/1	57	0
2	**Co–Ph**	0.1/2/2	76	0
3	**Co–Ph**	0.1/3/3	91	68
4	**Co–EtO**	0.1/1/1	74	51
5	**Co–EtO**	0.1/2/2	78	72
6	**Co–EtO**	0.1/3/3	78	79
7	**Co–Cl**	0.1/1/1	79	48
8	**Co–Cl**	0.1/2/2	82	69
9	**Co–Cl**	0.1/3/3	77	75
10	**Co–Me**	0.1/1/1	59	12
11	**Co–Me**	0.1/2/2	62	35
12	**Co–Me**	0.1/3/3	78	41
13	**Co–tBu**	0.1/1/1	0	0
14	**Co–*t*Bu**	0.1/2/2	65	0
15	**Co–*t*Bu**	0.1/3/3	76	51
16	---	0/1/1	60	48
17	---	0/2/2	64	57
18	---	0/3/3	70	70

**Table 2:** Final Conversions of TMPETA Achieved in the Laminate under LED Irradiation Using a 0.2 w % Photocatalyst and Different Amounts of Additives

entry	PC	Co^II^/Iod/EDB (w %/w %/w %)	conv. (%) at 365 nm LED	conv. (%) at 390–405 nm LED
1	**Co–Ph**	0.2/1/1	0	0
2	**Co–Ph**	0.2/2/2	71	0
3	**Co–Ph**	0.2/3/3	83	58
4	**Co–EtO**	0.2/1/1	74	71
5	**Co–EtO**	0.2/2/2	84	66
6	**Co–EtO**	0.2/3/3	84	67
7	**Co–Cl**	0.2/1/1	74	62
8	**Co–Cl**	0.2/2/2	76	67
9	**Co–Cl**	0.2/3/3	72	75
10	**Co–Me**	0.2/1/1	12	0
11	**Co–Me**	0.2/2/2	51	0
12	**Co–Me**	0.2/3/3	61	12
13	**Co–*t*Bu**	0.2/1/1	0	0
14	**Co–*t*Bu**	0.2/2/2	45	0
15	**Co-*t*Bu**	0.2/3/3	49	13

**Figure 2:**
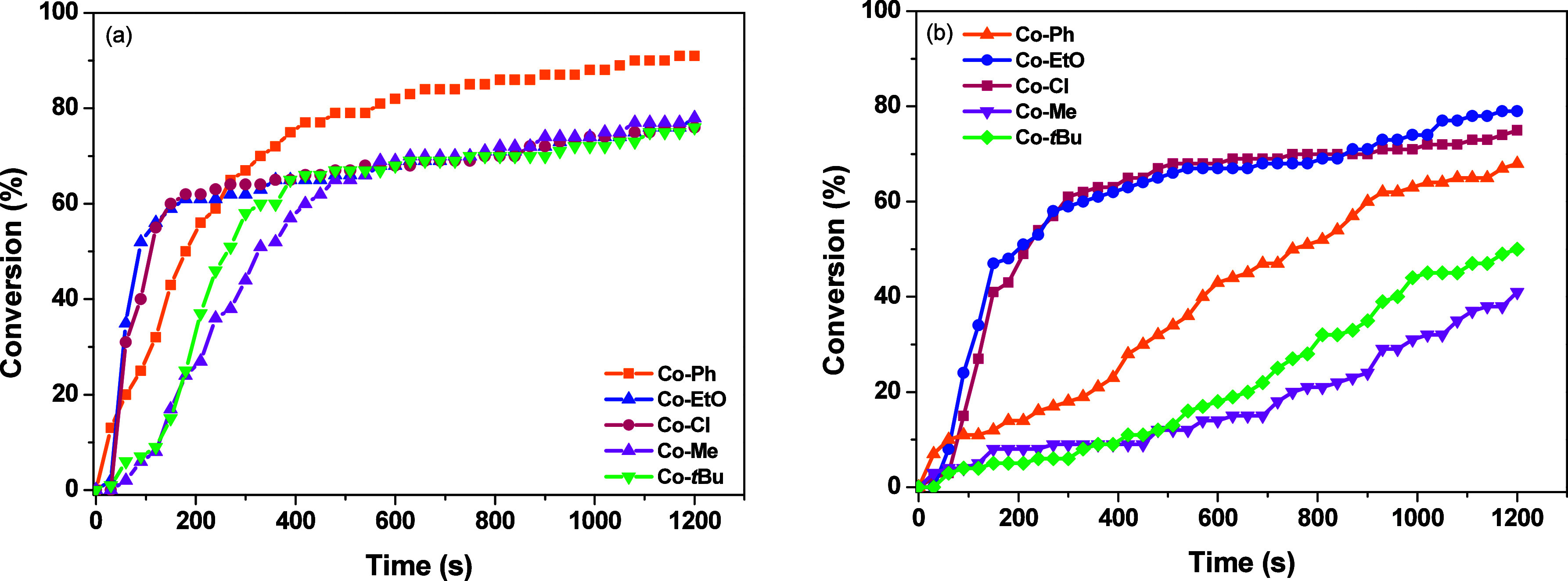
Conversions of TMPETA and irradiation time in laminate, using different photocatalysts with 0.1%/3%/3% w/w/w for Co^II^/Iod/EDB. (a) LED@365 nm and (b) LED@390–405 nm.

**Figure 3:**
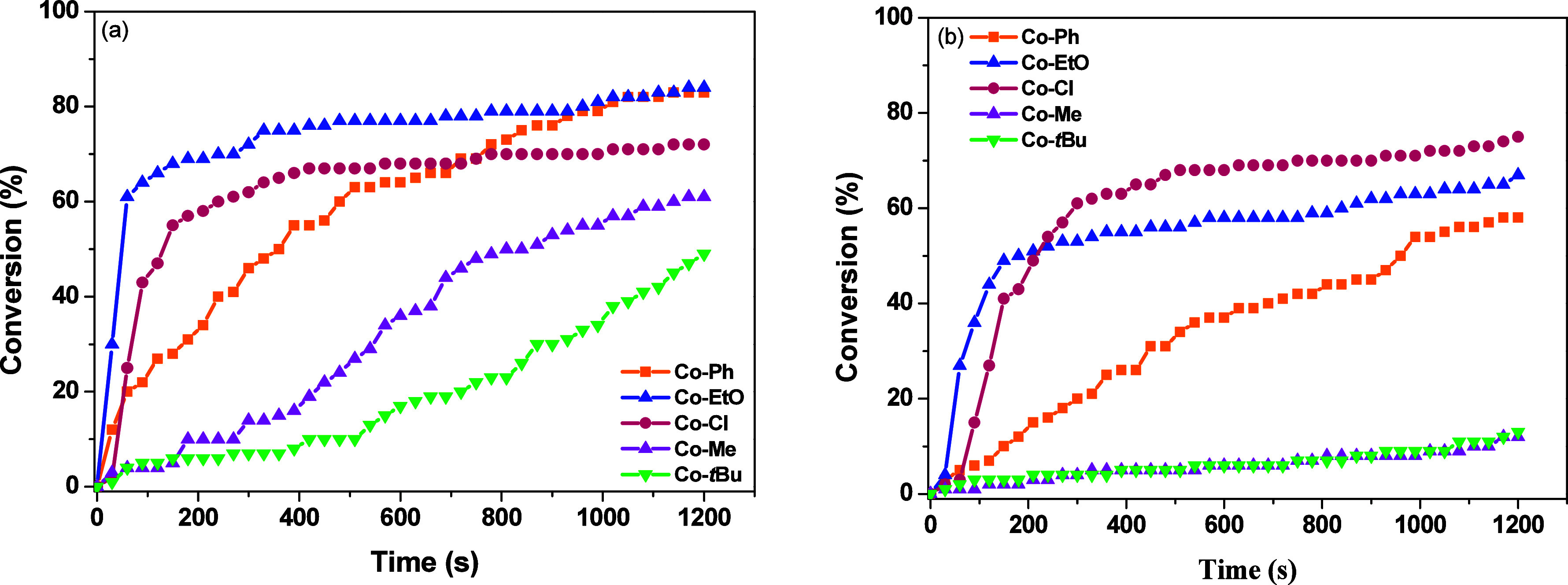
Conversions of TMPETA and irradiation time in laminate, using different photocatalysts with 0.2%/3%/3% w/w/w Co^II^/Iod/EDB. (a) LED@365 nm and (b) LED@390–405 nm.

To evaluate the catalytic performance of the Co­(II) complexes, initial photopolymerizations were performed using 0.1 w % of PC and different amounts of Iod and EDB (1, 2, or 3 w %) under LED irradiation at 365 and 390–405 nm, wavelengths selected based on the higher molar absorptivity of the complexes (Figure 1 and Table S7). The investigated Co­(II) complexes exhibited good catalytic activity when irradiated at 365 nm; for example, **Co–Ph** reached a high monomer conversion (91%) within 1200 s (Figure 2a, entry 3, Table 1).

In contrast, the reduced additive content led to moderate polymerization under LED@365 and LED@390–405 nm, with monomer conversions around 60%. No polymerization was observed for **Co–Ph** and **Co–** *t* **Bu** in entries 1, 2, 13, and 14 under LED@390–405 nm (Table 1). Notably, **Co–EtO** and **Co–Cl** exhibited a good catalytic efficiency (entries 4–6, Table 1), achieving a high TMPETA conversion in shorter reaction times under both 365 and 390–405 nm irradiation, with an enhanced performance at higher additive amounts (Figure 2a,b). Overall, the Co­(II) complexes demonstrated good activity under the 0.1%/3%/3% (w/w/w for Co^II^/Iod/EDB) conditions, with final conversions exceeding 76% after 1200 s of irradiation at 365 nm, without any inhibition period (Figure 2a). Under LED@390–405 nm, **Co**–**Ph**, **Co**–**EtO**, and **Co–Cl** maintained good catalytic activity, reaching conversions of 68, 79, and 75%, respectively (entries 3, 6, and 9, Table 1).

Photopolymerization experiments conducted without PC resulted in lower conversions and an inhibition period under certain conditions, indicating radical generation through charge-transfer complex between Iod and EDB under both LED@365 nm and LED@390–405 nm (Figure S30).^24,29,50,53^

The performance of the photoinitiating system was further evaluated through a series of photopolymerization experiments using 0.2 wt % PC (Table 2). An increase in the amount of **Co–EtO** led to higher TMPETA conversions, with a marked enhancement in the polymerization efficiency relative to entries 4–6 in Table 2. Moderate conversions or no polymerization were observed when using 0.2%/1%/1% and 0.2%/2%/2% (w/w/w for Co^II^/Iod/EDB) for **Co–Me** and **Co–** *t* **Bu** under LED@365 nm and LED@390–405 nm (Figures S33 and S34). The optimal condition was identified as 0.2%/3%/3% (w/w/w for Co^II^/Iod/EDB), under which TMPETA conversion was observed for all Co­(II) complexes when irradiated with both LED@365 nm and LED@390–405 nm (Figure 3a,b). Among the tested complexes, **Co–EtO** and **Co–Cl** exhibited the best performance, reaching conversions above 60% within 300 s. This behavior is attributed to the superior light absorption capacity of these complexes in the 365 and 390–405 nm regions. In addition, **Co–Cl** exhibits emission at longer wavelengths compared with the other complexes.

At the optimal condition of 0.2%/3%/3% w/w/w, **Co**–**EtO** exhibited a superior catalytic activity under all tested LED irradiations (Figure 4). Markedly, **Co**–**EtO** achieved a final monomer conversion of 63% within 1200 s under LED@420 nm, whereas no polymerization was observed for the other Co­(II) complexes. These results are attributed to the superior optical and electrochemical properties of **Co–EtO**, which demonstrate a higher molar absorptivity in the visible region and a lower oxidation potential relative to the other Co­(II) complexes investigated.

**Figure 4:**
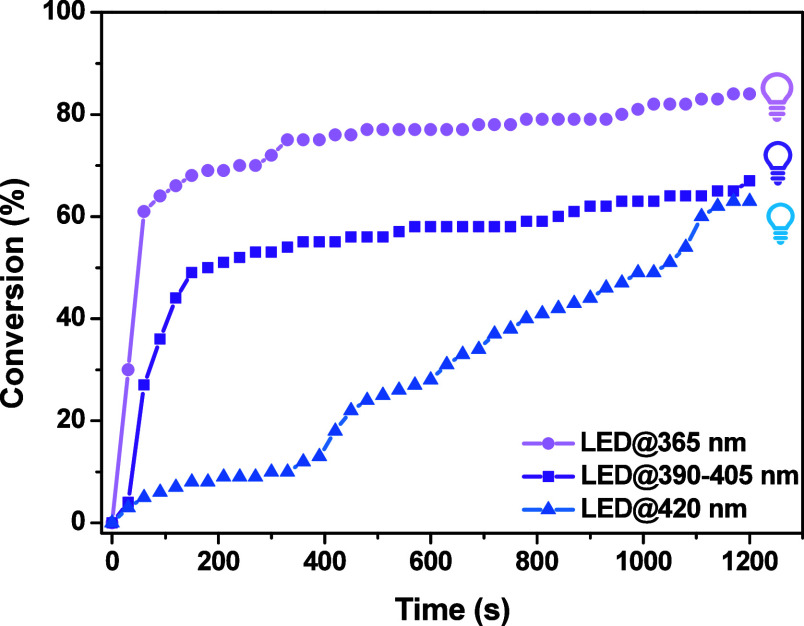
Conversions of TMPETA and irradiation time in the laminate, using 0.2%/3%/3% w/w/w **Co**–**EtO**/Iod/EDB under different LED irradiations.

The influence of light on the FRP reactions was demonstrated through an on–off light modulation experiment conducted under optimal conditions (LED@365 nm, 0.2%/3%/3% w/w/w for **Co**–**EtO**/Iod/EDB) (Figure 5). Notably, TMPETA conversion occurred predominantly during the periods when the UV light was on, while no conversion was detected during the dark intervals. The photoinitiating system was evaluated over successive 30 s on–off cycles, confirming that the polymerization process is light dependent and can be precisely controlled by LED irradiation, enabling the accurate temporal regulation of the reaction. In a previous study, a Ni­(II) complex bearing Schiff base ligands exhibited similar behavior during on–off experiments, where light reactivated dormant surface species during FRP reactions.^29^

**Figure 5:**
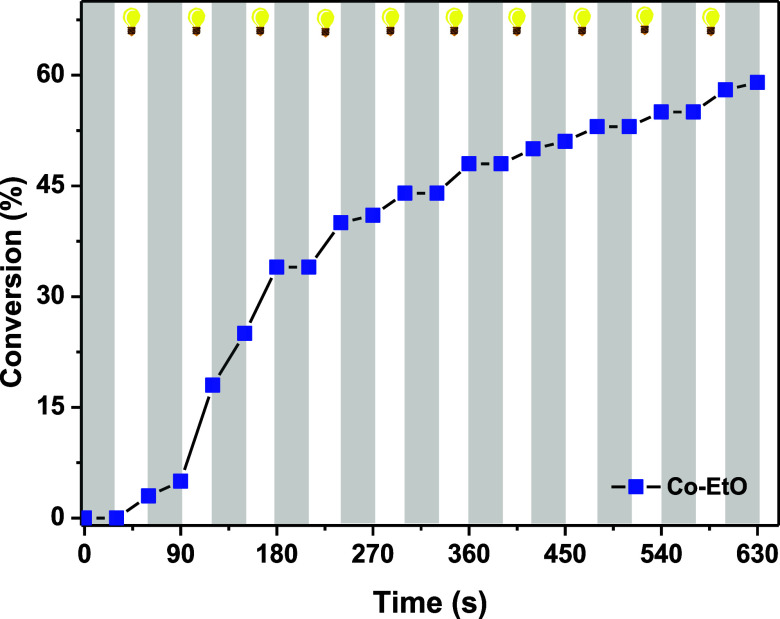
On and off experiment of conversions of TMPETA and irradiation time in the laminate, using 0.2%/3%/3% w/w/w for **Co**–**EtO**/Iod/EDB under LED@365 nm.

To further characterize the polymers obtained under the optimal conditions for each PC at LED@365 nm, the thermal stability of the TMPETA polymer was evaluated by TGA (Figure 6). The polymers prepared under UV irradiation in the presence of the Co­(II) complexes exhibited similar thermal profiles, showing a continuous weight loss starting at around 220 °C for all systems. The polymer obtained using the **Co–Cl** was not analyzed by TGA due to the presence of chlorine substituents, which could interfere with the thermal decomposition profile. According to the TGA data, the main decomposition of the TMPETA polymer was evidenced by a sharp weight loss near 300 °C.

**Figure 6:**
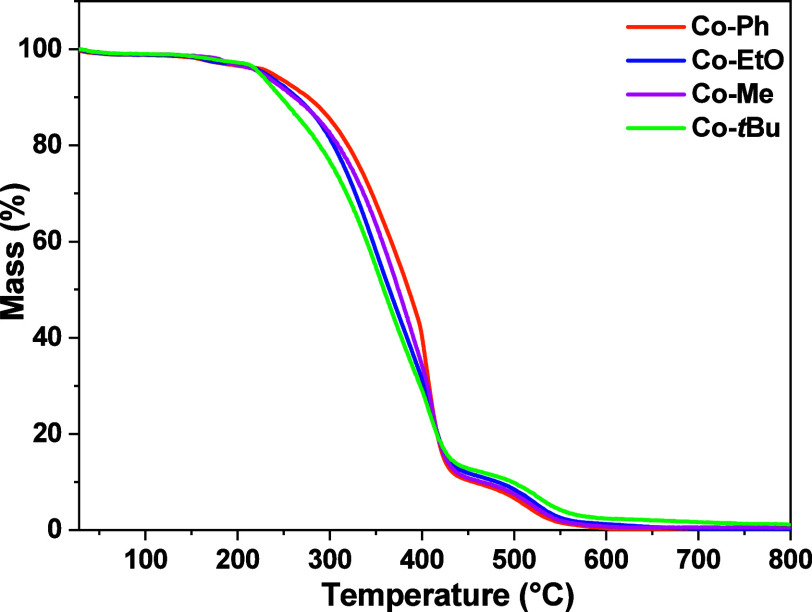
TGA curves of TMPETA polymers prepared under LED@365 nm irradiation using different Co­(II)/Iod/EDB systems.

### 3.2 Investigation of Interaction between Co­(II) Complexes and Additives

The redox reaction between the PC and additives was evaluated through photolysis experiments under LED@365 nm, where the PC exhibited a superior catalytic activity compared to irradiations at 390–405 and 420 nm. Initially, the stability of the Co­(II) complexes under LED irradiation was assessed, as evidenced by the absence of changes in the absorption spectra (Figures S35–S39). Regarding the interaction between Co­(II) complexes and EDB, no significant change in absorption was observed for **Co**–**Ph**, **Co–Cl**, **Co**–**Me**, and **Co**–*t* **Bu**, indicating that no reaction occurs between these Co­(II) complexes and EDB (Figures S36–S39). However, an increase in absorption bands within the 280–650 nm range was observed for **Co**–**EtO**, attributed to its reduction reaction with EDB and the consequent generation of radicals (Figure 7a).^49,52^

**Figure 7:**
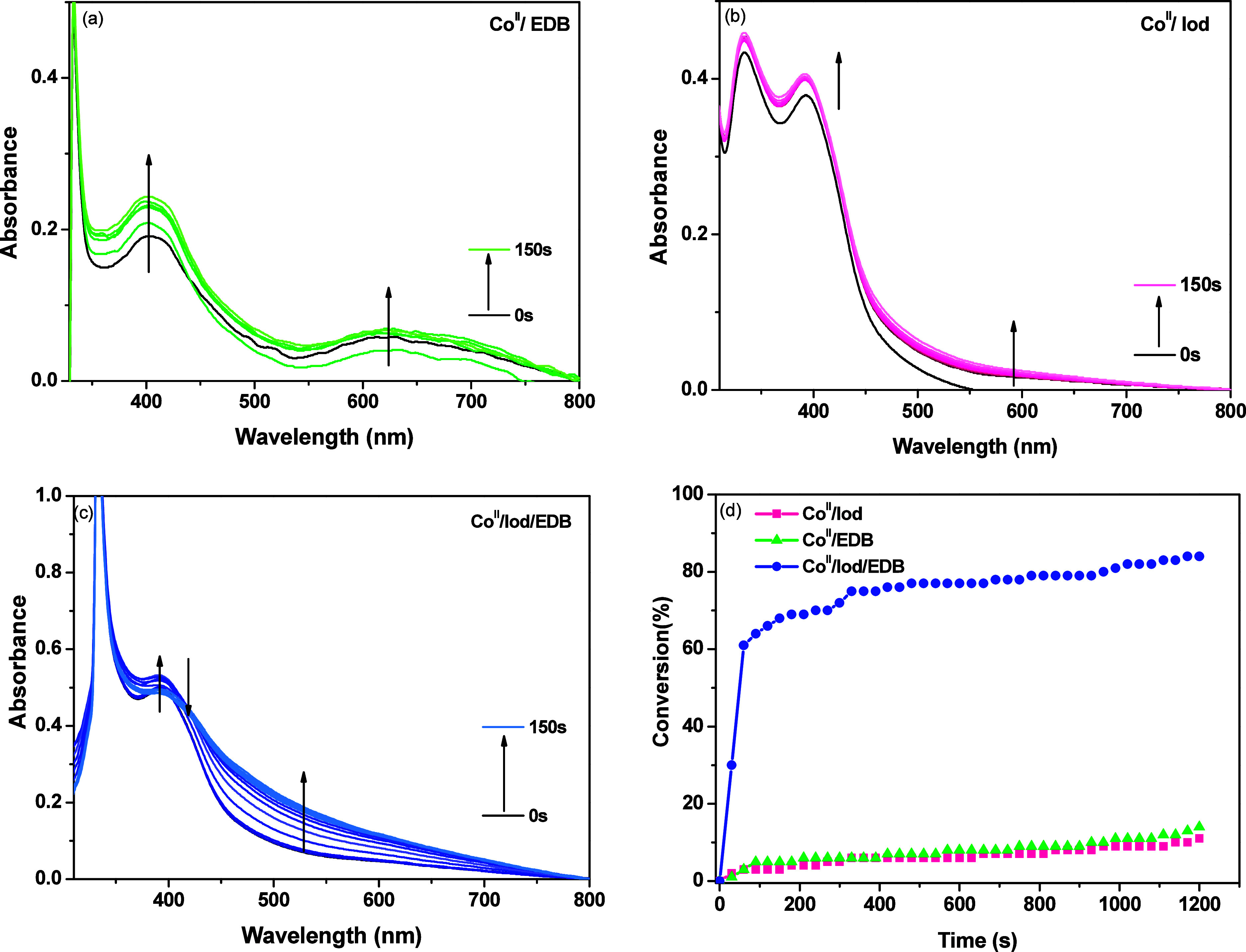
UV–vis absorption spectra of **Co**–**EtO** in CH_2_Cl_2_ and FRP experiments under LED@365 nm for different times; (a) **Co**–**EtO**/EDB, (b) **Co**–**EtO**/Iod, (c) **Co**–**EtO**/Iod/EDB, and (d) FRP conversion profiles using 0.2%/3%/3% w/w/w for **Co**–**EtO** and Iod/EDB.

On the other hand, Co­(II) complexes demonstrated significant interactions with Iod, indicated by changes in absorption bands (Figures 7b, S36 and S37). Notably, **Co**–**EtO** exhibited an increase in absorption, suggesting the formation of photoproducts in the presence of Iod (Figure 7b).^24,49,52^ For the remaining complexes, the observed decreases in absorption correspond to the Co­(II) consumption associated with electron transfer to the Iod (Figures S36 and S37).

In the three-component system (Co^II^/Iod/EDB), the absorption bands increased more compared to systems containing only one additive (Figures S36–S39). However, **Co**–**EtO** exhibited both increases (0–40 s) and decreases (40–150 s) in absorption between 300 and 500 nm, which can be attributed to the formation of photoproducts and radical generation (Figure 7c).

To gain insights into the interaction between **Co**–**EtO** and the additives, FRP experiments were conducted using two-component systems (PC/Iod and PC/EDB) and compared to a three-component system (PC/Iod/EDB) under an LED@365 nm (Figure 7d). In the two-component systems, low conversions (<15%) were observed within 1200 s using **Co**–**EtO**/Iod and **Co**–**EtO**/EDB, indicating that radical generation can proceed via oxidative or reductive pathways, respectively. Nevertheless, the three-component system proved to be more effective, which is attributed to the efficient regeneration of the photocatalyst.

### 3.3 Proposed Mechanism of FRP

The proposed photocatalytic mechanism is divided into three steps, based on experimental data and literature under similar conditions (Scheme 2).^24,29,49,52^ In step 1, light absorption promotes the Co­(II) complexes to an excited state (Co­(II)*), enabling electron-transfer reactions with the co-initiators, Iod and EDB. The specific pathway (oxidative or reductive) is dictated by the redox potentials of the photocatalysts and co-initiators, as well as the thermodynamic favorability of the electron transfer, quantified by the free energy change (Δ*G*_et_). For **Co–Ph**, **Co–Cl**, **Co–Me**, and **Co–** *t* **Bu**, the primary mechanism proceeds via an oxidative pathway. Upon excitation, these Co­(II)* complexes interact with Iod (*E*_red_ = −0.20 V vs SCE). The Co­(II)/(III) oxidation potentials of these complexes are sufficiently negative to allow for electron transfer to Iod, generating highly reactive phenyl radicals (Ph^•^) and a Co­(III) species. The free energy changes for these interactions (Δ*G*_et_^Iod^ ranging from −2.27 to −2.50 eV, Table S10) confirm the thermodynamic favorability of this oxidative process. Subsequently, the resulting Co­(III) species is regenerated back to Co­(II) through the interaction with EDB (*E*_ox_ = 1.00 V vs SCE), via electron transfer from EDB to the Co­(III) complex (Scheme 2). In contrast, **Co**–**EtO** exhibits a unique ability to follow two distinct mechanisms: both oxidative and reductive pathways. Its more favorable oxidation potential (−0.29 V vs Ag/AgCl, Table S8) allows for efficient interactions with Iod, leading to Ph^•^ radical generation via the oxidative cycle (Δ*G*_et_^Iod^ = −2.94 eV, Table S10). Crucially, **Co**–**EtO** also possesses a sufficiently negative Co­(II)/(I) reduction potential (−1.08 V vs Ag/AgCl, Table S9) to interact with EDB, enabling a reductive pathway where the excited complex is reduced by EDB, generating EDB^•+^ radicals (Δ*G*_et_^EDB^ = −0.79 eV, Table S10). This dual reactivity is attributed to the electronic influence of the ethoxy substituent, which modulates the redox properties of the cobalt center, making both electron-transfer processes thermodynamically viable. In the reductive pathway, the Co­(I) complex formed is regenerated back to Co­(II) by transferring an electron to Iod, generating an additional Ph^•^ radical (Scheme 2). Step 2 involves the initiation of polymerization, where the radicals generated from the redox reactions between the photoexcited Co­(II)* complex and Iod or EDB add to the trimethylolpropane ethoxylate triacrylate (TMPETA) monomer under LED irradiation, yielding a monomer-centered radical (Scheme 2). In step 3, the propagation of the radical occurs through successive additions of monomer units, resulting in polymer chain growth (Scheme 2).

**Scheme 2:**
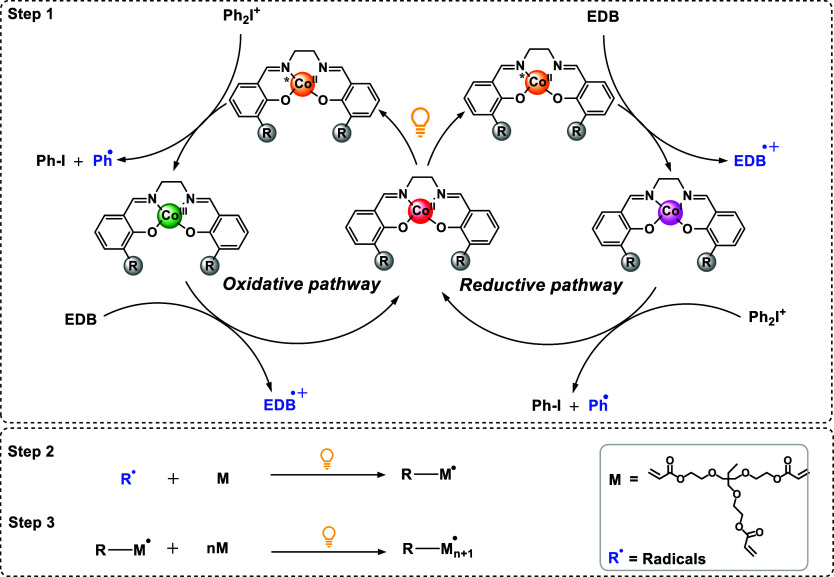
Proposed Mechanism for the Redox Reactions in the Co­(II)/Iod/EDB System

## 4 Conclusions

In this study, a comprehensive series of five Co­(II)–Schiff base complexes, including the newly synthesized **Co–Ph**, was successfully developed and thoroughly characterized as efficient photocatalysts for LED-FRP. All complexes demonstrated the effective photopolymerization of TMPETA in combination with Iod and EDB under UV (365 nm) and violet (390–405 nm) LED irradiation. Notably, **Co**–**EtO** consistently exhibited the highest photocatalytic efficiency across all tested wavelengths, including blue light (420 nm), achieving high monomer conversions and rapid polymerization rates under optimized conditions (0.2%/3%/3% w/w/w for Co^II^/Iod/EDB). On/off light experiments confirmed the light-dependent nature of the FRP process. The superior performance of **Co**–**EtO** is attributed to the synergistic interplay of its unique structural, electronic, and electrochemical properties. DFT calculations revealed that the electron-donating ethoxy substituent in **Co**–**EtO** subtly modulates the electronic environment of the Co center, leading to optimized bond lengths and a more favorable electronic configuration. Crucially, detailed electrochemical studies demonstrated that **Co**–**EtO** possesses significantly more advantageous redox potentials compared with the other complexes. This optimized redox behavior facilitates more efficient electron transfer processes with both Iod and EDB, enabling its exceptional catalytic activity. Mechanistic investigations, supported by photolysis studies and free energy calculations, elucidated distinct pathways for the series. For **Co–Ph**, **Co–Cl**, **Co–Me**, and **Co–** *t* **Bu**, an oxidative pathway was predominantly observed, involving the interaction of the excited Co­(II)* complex with Iod to generate phenyl radicals, followed by the regeneration of the photocatalyst via EDB. In contrast, **Co**–**EtO** uniquely demonstrated the ability to engage in both oxidative and reductive pathways. This dual mechanistic capability underscores the versatility and enhanced efficiency of **Co**–**EtO**.

## Supplementary Material



## Notes

FTIR, UV–vis, ^1^H NMR, MALDI-TOF mass spectra, cyclic voltammetry, emission fluorescence spectra of Co­(II) complexes, FRP and photolysis experiments, and computational data (PDF)

## References

[1] Ferraro V., Adam C. R., Vranic A., Bräse S. (2024). Recent Advances
of Transition Metal Complexes for Photopolymerization and 3D Printing
under Visible Light. Adv. Funct. Mater..

[2] Rahal M., Graff B., Toufaily J., Hamieh T., Dumur F., Lalevée J. (2021). Design of Keto-Coumarin Based Photoinitiator
for Free
Radical Photopolymerization: Towards 3D Printing and Photocomposites
Applications. Eur. Polym. J..

[3] Bonardi A.-H., Dumur F., Noirbent G., Lalevée J., Gigmes D. (2018). Organometallic vs Organic Photoredox Catalysts for
Photocuring Reactions in the Visible Region. Beilstein J. Org. Chem..

[4] Balzani, V. ; Ceroni, P. ; Juris, A. Photochemistry and Photophysics: Concepts, Research, Applications, 2nd ed.; Blackwell Verlag: Berlim, Germany, 2024.

[5] Pirman T., Ocepek M., Likozar B. (2021). Radical Polymerization
of Acrylates,
Methacrylates, and Styrene: Biobased Approaches, Mechanism, Kinetics,
Secondary Reactions, and Modeling. Ind. Eng.
Chem. Res..

[6] Chatani S., Kloxin C. J., Bowman C. N. (2014). The Power of Light in Polymer Science:
Photochemical Processes to Manipulate Polymer Formation, Structure,
and Properties. Polym. Chem..

[7] Zivic N., Bouzrati-Zerelli M., Kermagoret A., Dumur F., Fouassier J.-P., Gigmes D., Lalevée J. (2016). Photocatalysts in Polymerization
Reactions. ChemCatChem.

[8] Garra P., Dietlin C., Morlet-Savary F., Dumur F., Gigmes D., Fouassier J.-P., Lalevée J. (2019). Redox Two-Component Initiated Free
Radical and Cationic Polymerizations: Concepts, Reactions and Applications. Prog. Polym. Sci..

[9] Braun D. (2009). Origins and
Development of Initiation of Free Radical Polymerization Processes. Int. J. Polym. Sci..

[10] Garra P., Dumur F., Gigmes D., Al Mousawi A., Morlet-Savary F., Dietlin C., Fouassier J. P., Lalevée J. (2017). Copper (Photo)­Redox Catalyst for Radical Photopolymerization
in Shadowed Areas and Access to Thick and Filled Samples. Macromolecules.

[11] Xu J., Jiang Y., Zhang T., Dai Y., Yang D., Qiu F., Yu Z., Yang P. (2018). Synthesis of UV-Curing Waterborne
Polyurethane-Acrylate Coating and Its Photopolymerization Kinetics
Using FT-IR and Photo-DSC Methods. Prog. Org.
Coat..

[12] Hou C., Gui Q. (2024). Preparation and Properties
of UV Curing Varnish Suited for Various
Substrates. J. Photochem. Photobiol., A.

[13] Andrzejewska, E. Free Radical Photopolymerization of Multifunctional Monomers. In Three-Dimensional Microfabrication Using Two-photon Polymerization; Elsevier, 2016; pp 62–81.

[14] Llorente O., Agirre A., Calvo I., Olaso M., Tomovska R., Sardon H. (2021). Exploring the Advantages
of Oxygen-Tolerant Thiol-Ene
Polymerization over Conventional Acrylate Free Radical Photopolymerization
Processes for Pressure-Sensitive Adhesives. Polym. J..

[15] Dickens S. H., Stansbury J. W., Choi K. M., Floyd C. J. E. (2003). Photopolymerization
Kinetics of Methacrylate Dental Resins. Macromolecules.

[16] Lalevée J., Fouassier J. P. (2011). Recent
Advances in Sunlight Induced Polymerization:
Role of New Photoinitiating Systems Based on the Silyl Radical Chemistry. Polym. Chem..

[17] Ribas-Massonis A., Cicujano M., Duran J., Besalú E., Poater A. (2022). Free-Radical Photopolymerization for Curing Products
for Refinish Coatings Market. Polymers.

[18] Khudyakov I. V. (2018). Fast Photopolymerization
of Acrylate Coatings: Achievements and Problems. Prog. Org. Coat..

[19] Sun K., Pigot C., Chen H., Nechab M., Gigmes D., Morlet-Savary F., Graff B., Liu S., Xiao P., Dumur F., Lalevée J. (2020). Free Radical Photopolymerization
and 3D Printing Using Newly Developed Dyes: Indane-1,3-Dione and 1H-Cyclopentanaphthalene-1,3-Dione
Derivatives as Photoinitiators in Three-Component Systems. Catalysts.

[20] Zhang Y., Song B., Dietlin C., Morlet-Savary F., Schmitt M., Dumur F., Lalevée J. (2024). Naphthoquinone-Based
Oxime Esters for Free Radical Photopolymerization under Sunlight or
a Blue Light-Emitting Diode. Ind. Eng. Chem.
Res..

[21] Borjigin T., Noirbent G., Gigmes D., Xiao P., Dumur F., Lalevée J. (2022). The New LED-Sensitive
Photoinitiators of Polymerization:
Copper Complexes in Free Radical and Cationic Photoinitiating Systems
and Application in 3D Printing. Eur. Polym.
J..

[22] Tehfe M.-A., Lepeltier M., Dumur F., Gigmes D., Fouassier J.-P., Lalevée J. (2017). Structural Effects in the Iridium Complex Series: Photoredox
Catalysis and Photoinitiation of Polymerization Reactions under Visible
Lights. Macromol. Chem. Phys..

[23] Al
Mousawi A., Kermagoret A., Versace D. L., Toufaily J., Hamieh T., Graff B., Dumur F., Gigmes D., Fouassier J. P., Lalevée J. (2017). Copper photoredox catalysts for polymerization
upon near UV or visible light: Structure/reactivity/efficiency relationships
and use in LED projector 3D printing resins. Polym. Chem..

[24] Pesqueira N. M., Morlet-Savary F., Schmitt M., Jouaiti A., Goi B. E., Mauro M., Lalevée J. (2025). Heteroleptic Copper­(I) Complexes
with Pyridine-Benzothiazole Ligands as Photocatalysts for Free Radical
Photopolymerization and 3D Printing. ACS Appl.
Polym. Mater..

[25] Sun G., Huang Y., Ma J., Li D., Fan Q., Li Y., Shao J. (2021). Photoinitiation Mechanisms and Photogelation Kinetics
of Blue Light Induced Polymerization of Acrylamide with Bicomponent
Photoinitiators. J. Polym. Sci..

[26] Zhang G., Song I. Y., Park T., Choi W. (2012). Recyclable and Stable
Ruthenium Catalyst for Free Radical Polymerization at Ambient Temperature
Initiated by Visible Light Photocatalysis. Green
Chem..

[27] Versace D.-L., Cerezo Bastida J., Lorenzini C., Cachet-Vivier C., Renard E., Langlois V., Malval J.-P., Fouassier J.-P., Lalevée J. (2013). A Tris­(Triphenylphosphine)­Ruthenium­(II)
Complex as
a UV Photoinitiator for Free-Radical Polymerization and in Situ Silver
Nanoparticle Formation in Cationic Films. Macromolecules.

[28] Lalevée J., Tehfe M.-A., Dumur F., Gigmes D., Blanchard N., Morlet-Savary F., Fouassier J. P. (2012). Iridium Photocatalysts in Free Radical
Photopolymerization under Visible Lights. ACS
Macro Lett..

[29] Pesqueira N. M., Morlet-Savary F., Schmitt M., Carvalho V. P., Goi B. E., Lalevée J. (2024). Advancing Photopolymerization and
3D Printing: High-Performance NiII Complexes Bearing N2O2 Schiff-Base
Ligands as Photocatalysts. Eur. Polym. J..

[30] Lalevée J., Telitel S., Xiao P., Lepeltier M., Dumur F., Morlet-Savary F., Gigmes D., Fouassier J.-P. (2014). Metal and
Metal-Free Photocatalysts: Mechanistic Approach and Application as
Photoinitiators of Photopolymerization. Beilstein
J. Org. Chem..

[31] Liu W., Sahoo B., Junge K., Beller M. (2018). Cobalt Complexes as
an Emerging Class of Catalysts for Homogeneous Hydrogenations. Acc. Chem. Res..

[32] Chirik P., Morris R. (2015). Getting down to Earth: The Renaissance
of Catalysis
with Abundant Metals. Acc. Chem. Res..

[33] Bullock R. M. (2013). Chemistry.
Abundant Metals Give Precious Hydrogenation Performance. Science.

[34] Kar K., Ghosh D., Kabi B., Chandra A. (2022). A Concise Review on
Cobalt Schiff Base Complexes as Anticancer Agents. Polyhedron.

[35] Bignardi C., Oliveira L. F., Pesqueira N. M., Riga-Rocha B. A., Machado A. E. H., Carvalho-Jr V. P., Goi B. E. (2022). Photoinduced Organometallic
Mediating Radical Polymerization of Acrylates Mediated by CoII Complexes
of Non-Symmetrical Tetradentate Schiff-Base Ligands. J. Photochem. Photobiol., A.

[36] Fleck M., Layek M., Saha R., Bandyopadhyay D. (2013). Synthetic
Aspects, Crystal Structures and Antibacterial Activities of Manganese­(III)
and Cobalt­(III) Complexes Containing a Tetradentate Schiff Base. Transit. Met. Chem..

[37] Pogány L., Moncol J., Gál M., Šalitroš I., Boča R. (2017). Four Cobalt­(III)
Schiff Base Complexes – Structural,
Spectroscopic and Electrochemical Studies. Inorg.
Chim. Acta.

[38] Oliveira L. F., Bignardi C., Pesqueira N. M., Riga–Rocha B. A., Machado A. E. H., Carvalho–Jr V.
P., Goi B. E. (2021). Photocontrolled
Reversible-Deactivation Radical Polymerization of Butyl Acrylate Mediated
by Salen-Type CoII Complexes. Eur. Polym. J..

[39] Pesqueira N. M., Lira K. H., Bignardi C., da Silva M. K. C., Silva T. F., Machado A. E. H., Nascimento O. R., Carvalho-Jr V. P., Goi B. E. (2024). Photo-induced Organo-manganese-mediated
Radical Polymerization
of Acrylates under LED Irradiation. Appl. Organomet.
Chem..

[40] Pesqueira N. M., Bignardi C., Oliveira L. F., Machado A. E. H., Carvalho-Jr V. P., Goi B. E. (2023). Visible Light-Induced
Radical Polymerization of Vinyl
Acetate Mediated by Organo-Nickel N2O2 Schiff-Base Complexes. J. Photochem. Photobiol., A.

[41] Silva T. T., Silva Y. F., Machado A. E. H., Maia P. I. S., Tasso C. R. B., Lima-Neto B. S., Silva Sá J. L., Carvalho-Jr V. P., Batista N. C., Goi B. E. (2019). Cycloalkyl-Substituted
Salicylaldimine-Nickel­(II)
Complexes as Mediators in Controlled Radical Polymerization of Vinyl
Acetate. J. Macromol. Sci., Part A:Pure Appl.
Chem..

[42] Riga B. A., Silva Y. F., Nascimento O. R., Machado A. E. H., Carvalho-Jr V. P., Goi B. E. (2020). Cobalt­(II) Complexes of α-Diimine Derived from
Cycloalkylamines as Controlling Agents for Organometallic Mediated
Radical Polymerization of Vinyl Acetate. Polyhedron.

[43] Afonso M. B. A., Gonçalves L. G., Silva T. T., Sá J. L. S., Batista N. C., Goi B. E., Carvalho Júnior V. P. (2018). Synthesis
of Poly­(Ethyl Methacrylate-Co-Methyl Methacrylate) Obtained via ATRP
Using Ruthenium Benzylidene Complexes. Polymers.

[44] Figueiredo M. L. B., Bignardi C., Pesqueira N. M., Machado A. E. H., Carvalho-Jr V. P., Nascimento O. R., Goi B. E. (2024). Well-Defined Non-Symmetric NHC-Iron­(III)
Catalyst for Photoinduced Atom-Transfer Radical Polymerization of
Methyl Methacrylate. J. Photochem. Photobiol.,
A.

[45] Afonso M. B. A., Cruz T. R., Silva Y. F., Pereira J. C. A., Machado A. E. H., Goi B. E., Lima-Neto B. S., Carvalho-Jr V. P. (2017). Ruthenium­(II)
Complexes of Schiff Base Derived from Cycloalkylamines as Pre-Catalysts
for ROMP of Norbornene and ATRP of Methyl Methacrylate. J. Organomet. Chem..

[46] Cruz T. R., Silva E. A., Oliveira D. P., Martins D. M., Gois P. D. S., Machado A. E. H., Maia P. I. S., Goi B. E., Lima-Neto B. S., Carvalho-Jr V. P. (2020). Dual Catalytic
Performance of Arene-ruthenium
Amine Complexes for Norbornene Ring-opening Metathesis and Methyl
Methacrylate Atom-transfer Radical Polymerizations: Arene-Ruthenium
Amines Complexes as Dual Catalysts for ROMP and ATRP. Appl. Organomet. Chem..

[47] Borim P., Lima-Neto B. S., Goi B. E., Carvalho V. P. (2017). Ru-Dimethyl Sulfoxide Complexes as Catalysts Precursors for ROMP
of Norbornene and ATRP of Methyl Methacrylate. Inorg. Chim. Acta.

[48] Silva R. A. N., Borim P., Fonseca L. R., Lima-Neto B. S., Silva Sá J. L., Carvalho-Jr V. P. (2017). Non-Carbene
Complex [RuCl2­(PPh3)­2­(Azocane)]
as Active Catalyst Precursor for ROMP and ATRP. Catal. Lett..

[49] Bignardi C., Pesqueira N. M., Shimizo Y. M., Machado A. E. H., Araújo D. M. S., Nascimento O. R., Carvalho V. P., Lalevée J., Goi B. E. (2025). Manganese­(II)-PyNHC
Complex as Visible-Light-Triggered Photocatalyst for Photopolymerization
of Acrylates and 3D Printing. ACS Appl. Polym.
Mater..

[50] Yamada S. M. M., Figueiredo M. L. B., Pesqueira N. M., Fantuzzi F., Carvalho-Jr V. P., Goi B. E. (2025). Iron­(II)–Schiff
Base Complexes as Photocatalysts for Controlled Radical Photopolymerization
under Light Emitting Diode Irradiation. Eur.
J. Inorg. Chem..

[51] Silva Y. F., Riga B. A., Deflon V. M., Souza J. R., Silva L. H. F., Machado A. E. H., Maia P. I. S., Valdemiro
P C. J., Goi B. E. (2018). Organometallic-Mediated Radical Polymerization Using
Well-Defined Schiff Base Cobalt­(II) Complexes. J. Coord. Chem..

[52] Pesqueira N. M., Morlet-Savary F., Schmitt M., Carvalho V. P., Goi B. E., Lalevée J. (2025). Visible Light-Promoted Nickel-NHC
Photocatalysts for
Free Radical Photopolymerization and 3D Printing Application. Polym. Chem..

[53] Akselsen Ø. W., Skattebøl L., Hansen T. V. (2009). ortho-Formylation
of oxygenated phenols. Tetrahedron Lett..

[54] Hansen, T. V. , Skattebøl, L. ortho -Formylation of Phenols; Preparation of 3-Bromosalicylaldehyde. Org. Synth.; John Wiley & Sons, 2005; Vol. 82, pp 64–68.

[55] Hofsløkken N. U., Skattebøl L., Johansson F., Bertilsson S. K., Andersson P. G., Møller J., Senning A., Yao X. K., Wang H. G., Tuchagues J. P. (1999). Convenient Method for
the ortho-Formylation of Phenols. Acta Chem.
Scand..

[56] Neese F. (2022). Software update:
The ORCA program system—Version 5.0. Wiley Interdiscip. Rev.:Comput. Mol. Sci..

[57] Ernzerhof M., Scuseria G. E. (1999). Assessment of the Perdew–Burke–Ernzerhof
exchange-correlation functional. J. Chem. Phys..

[58] Adamo C., Barone V. (1999). Toward reliable density functional methods without
adjustable parameters: The PBE0 model. J. Chem.
Phys..

[59] Weigend F., Ahlrichs R. (2005). Balanced basis sets
of split valence, triple zeta valence
and quadruple zeta valence quality for H to Rn: Design and assessment
of accuracy. Phys. Chem. Chem. Phys..

[60] Grimme S., Antony J., Ehrlich S., Krieg H. (2010). A consistent and accurate
ab initio parametrization of density functional dispersion correction
(DFT-D) for the 94 elements H-Pu. J. Chem. Phys..

[61] Grimme S., Ehrlich S., Goerigk S. (2011). Effect of the damping function in
dispersion corrected density functional theory. J. Comput. Chem..

[62] Neese F. (2003). An improvement
of the resolution of the identity approximation for the formation
of the Coulomb matrix. J. Comput. Chem..

[63] Neese F., Wennmohs F., Hansen A., Becker U. (2009). Efficient, approximate
and parallel Hartree–Fock and hybrid DFT calculations. A ‘chain-of-spheres’
algorithm for the Hartree–Fock exchange. Chem. Phys..

[64] Najubi A., Goerigk L. (2020). DFT-D4 counterparts
of leading meta-generalized-gradient
approximation and hybrid density functionals for energetics and geometries. J. Chem. Theory Comput..

[65] Craciunescu L., Wirsing S., Hammer S., Broch K., Dreuw A., Fantuzzi F., Sivanesan V., Tegeder P., Engels B. (2022). Accurate Polarization-Resolved
Absorption Spectra of Organic Semiconductor Thin Films Using First-Principles
Quantum-Chemical Methods: Pentacene as a Case Study. Chem. Lett..

[66] Craciunescu L., Asbach M., Wirsing S., Hammer S., Unger F., Broch K., Schreiber F., Witte G., Dreuw A., Tegeder P., Fantuzzi F., Engels B. (2023). Cluster-Based Approach
Utilizing Optimally Tuned TD-DFT to Calculate Absorption Spectra of
Organic Semiconductor Thin Films. J. Chem. Theory
Comput..

[67] Yáñez-S M., Moya S. A., Zúñiga C., Cárdenas-Jirón G. (2017). Theoretical
assessment of TD-DFT applied to a ferrocene-based complex. Comput. Theor. Chem..

[68] Das D., Roy A., Sutradhar S., Fantuzzi F., Ghosh B. N. (2023). A Simple Copper
(II) dppy-based Receptor for Sensing of L-cysteine and L-histidine
in Aqueous Acetonitrile Medium. Diagn.

[69] Martin R. L. (2003). Natural
transition orbitals. J. Chem. Phys..

[70] Dennington, R. ; Keith, T. A. ; Millam, J. M. GaussView. Version 6; Semichem Inc.: Shawnee Mission, KS, 2016.

[71] Bendia S., Bourzami R., Weiss J., Ouari K. (2021). Structural
investigation
of the catalytic activity of Fe­(III) and Mn­(III) Schiff base complexes. Polyhedron.

[72] Plitt P., Pritzkow H., Oeser T., Kraemer R. (2005). Biphenyl derived oxovanadium­(IV)
and copper­(II) salen-type complexes--structure and redox tuning. J. Inorg. Biochem..

[73] Kleij A. W. (2009). Nonsymmetrical
Salen Ligands and Their Complexes: Synthesis and Applications. Eur. J. Inorg. Chem..

[74] Ceyhan G., Köse M., McKee V., Urus S., Gölcü A., Tümer M. (2012). Tetradentate Schiff base ligands and their complexes:
Synthesis, structural characterization, thermal, electrochemical and
alkane oxidation. Spectrochim. Acta, Part A.

[76] Deligonul N., Tumer M., Serin S. (2006). Synthesis
characterization, catalytic,
electrochemical and thermal properties of tetradentate Schiff base
complexes. Transition Met. Chem..

[77] Kianfara A. H., Zargari S., Khavasi H. R. (2010). Synthesis and electrochemistry
of
M­(II) N2O2 schiff base complexes: X-Ray structure of {Ni­[Bis­(3-chloroacetylacetone)­ethylenediimine]}. J. Iran. Chem. Soc..

[78] Ourari A., Messali S., Bouzerafa B., Ouennoughi Y., Aggoun D., Mubarak M. S., Strawsine L. M., Peters D. G. (2015). Synthesis, characterization, and electrochemical behavior
of a cobalt­(II) salen-like complex. Polyhedron.

