# Supplementary material for: Tailoring Cobalt(II) Schiff Base Photocatalysts for Enhanced LED-Induced Free Radical Polymerization
Source: ACS Polym Au. 2025 Nov 10;5(6):944–55. doi: 10.1021/acspolymersau.5c00112 (PMC12874166; doi:10.1021/acspolymersau.5c00112)
Supplement: Supplementary file 1 [file lg5c00112_si_001.pdf]

Supporting Information

Tailoring Cobalt(II) Schiff Base Photocatalysts for enhanced LED-Induced Free Radical Polymerization

Larissa Fazioni de Oliveira, Naralyne Martins Pesqueira, Yasmin de Morais Shimizo, Maria Luísa Botter Figueiredo, Valdemiro P. Carvalho-Jr, Beatriz Eleutério Goi*

São Paulo State University (Unesp), School of Technology and Sciences, Presidente Prudente, SP, 19060-900, Brazil

*Corresponding author:

B. E. Goi (e-mail: beatriz.goi@unesp.br)

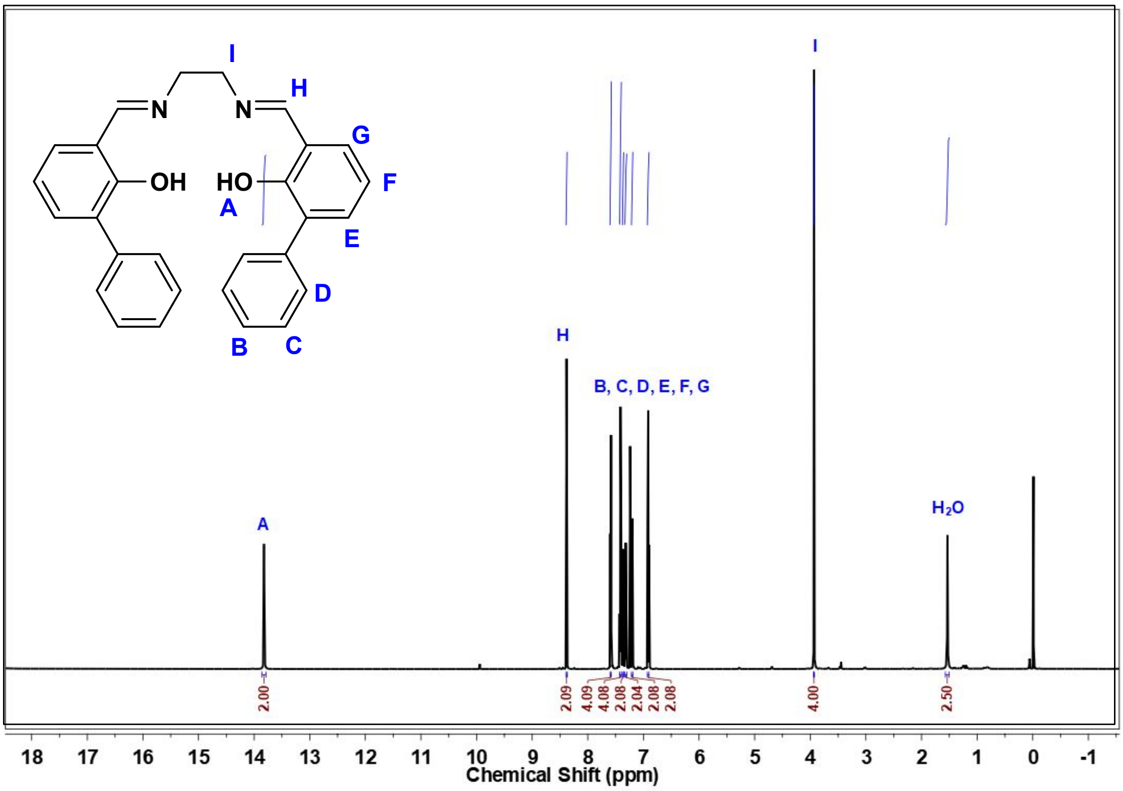

Figure S1. ^{1}H NMR spectrum for Salen-Ph in $CDCl_3$.

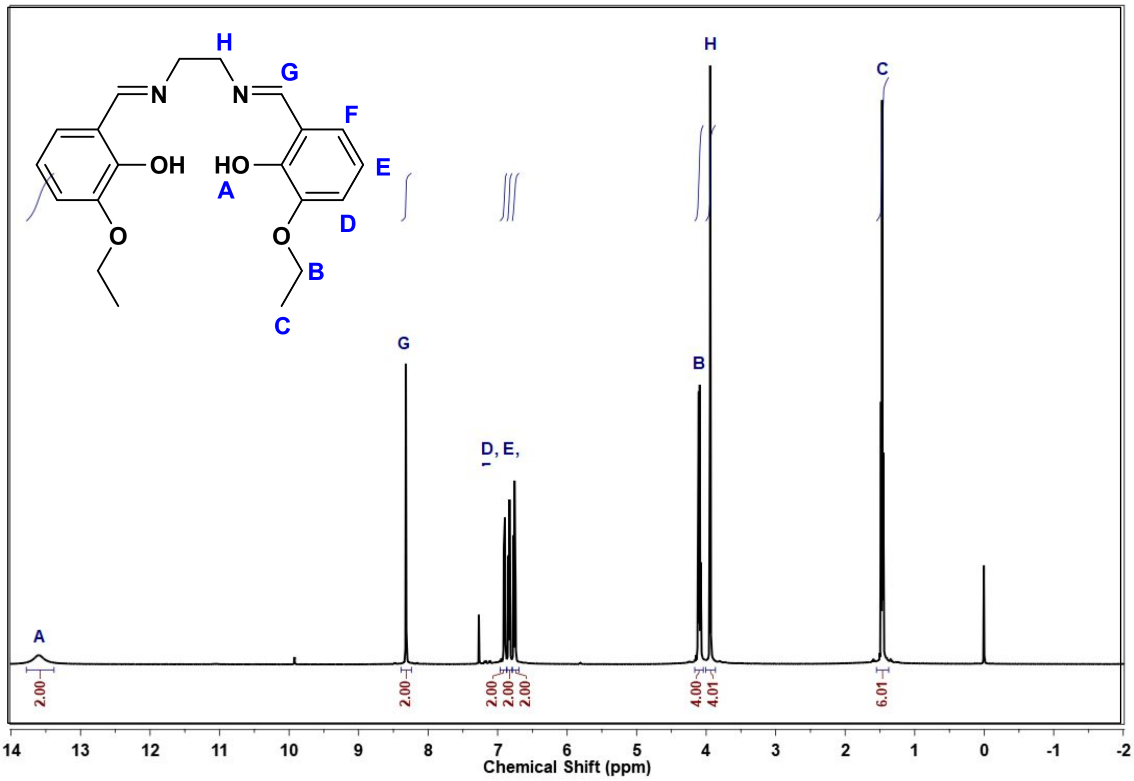

Figure S2. ^{1}H NMR spectrum for Salen-EtO in $CDCl_3$.

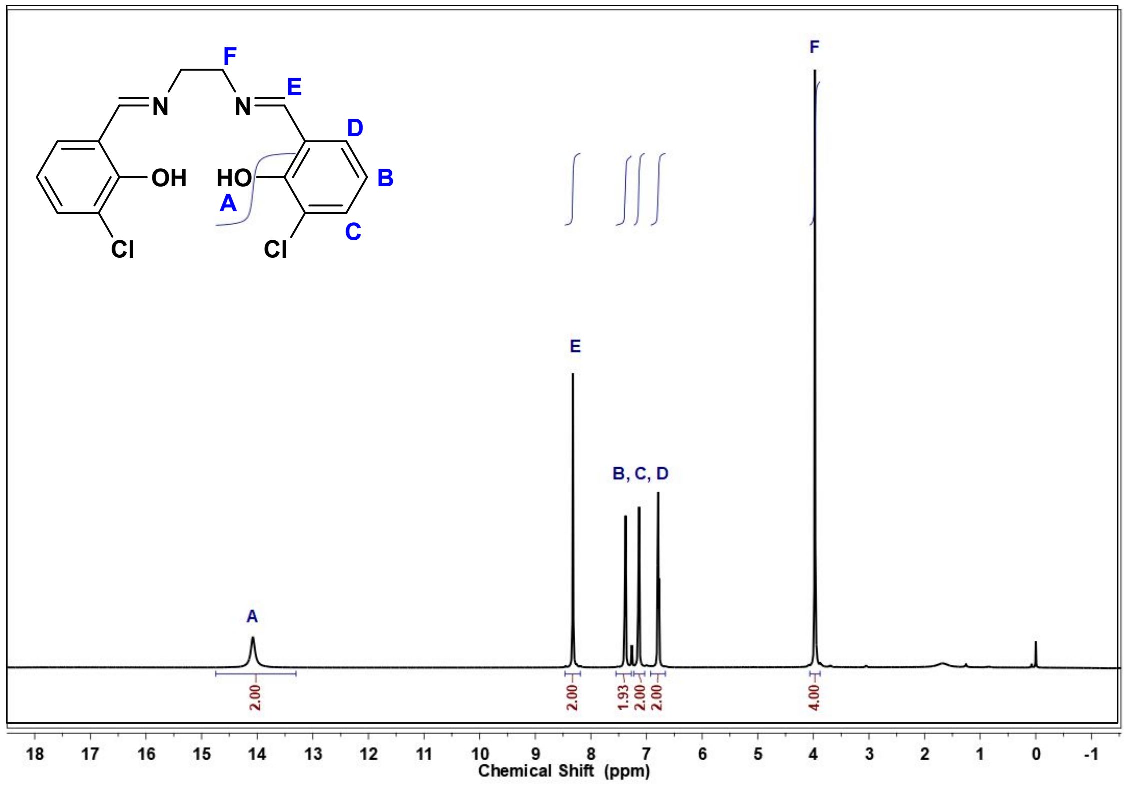

Figure S3. ^{1}H NMR spectrum for Salen-Cl in $CDCl_3$.

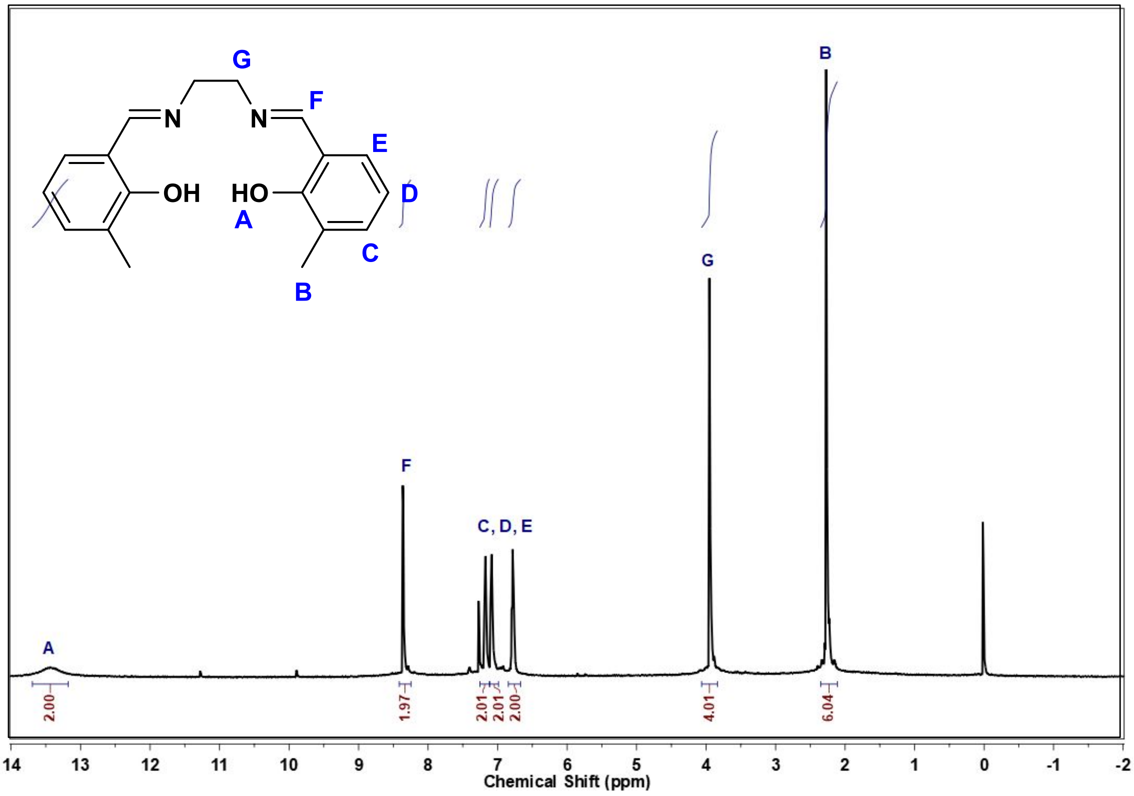

Figure S4. ^{1}H NMR spectrum for Salen-Me in $CDCl_3$.

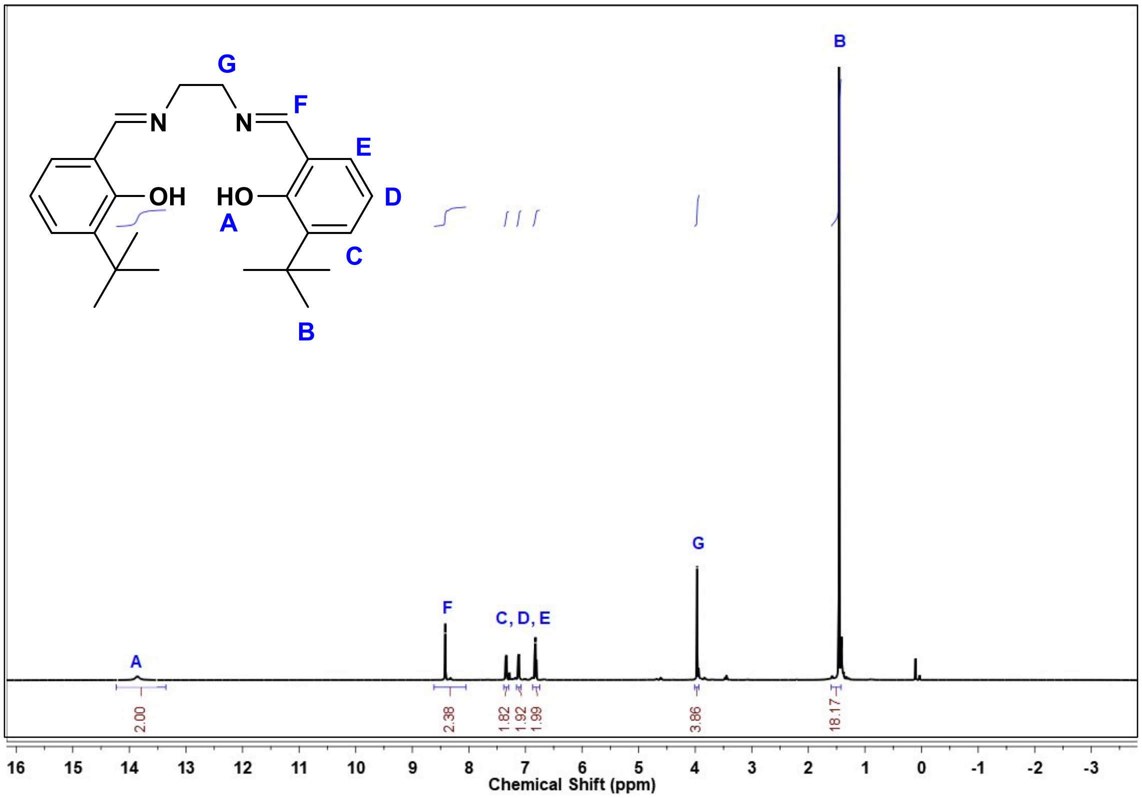

Figure S5. ^{1}H NMR spectrum for Salen-*t*Bu in $CDCl_3$.

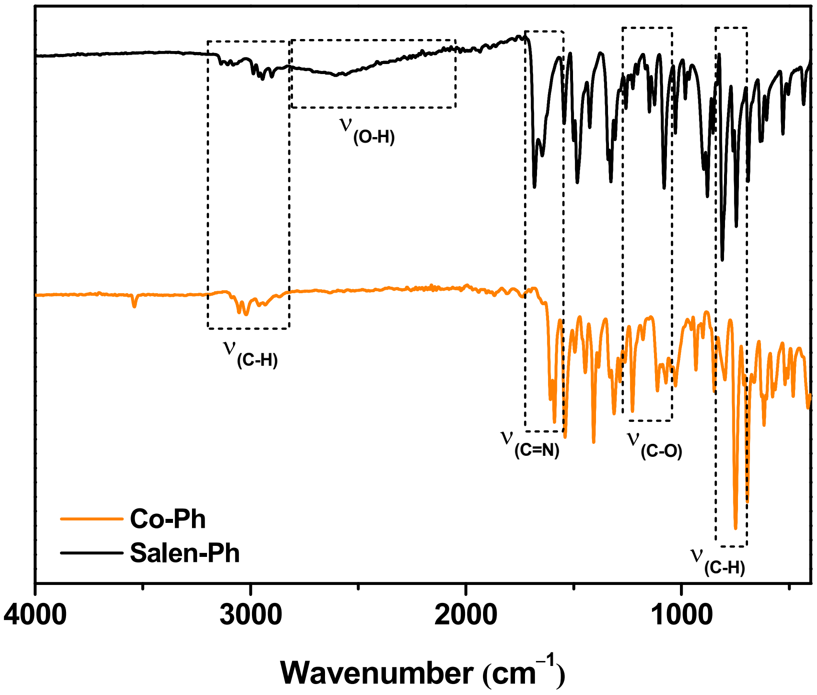

Figure S6. FTIR spectra for **Co-Ph** and ligand Salen-Ph.

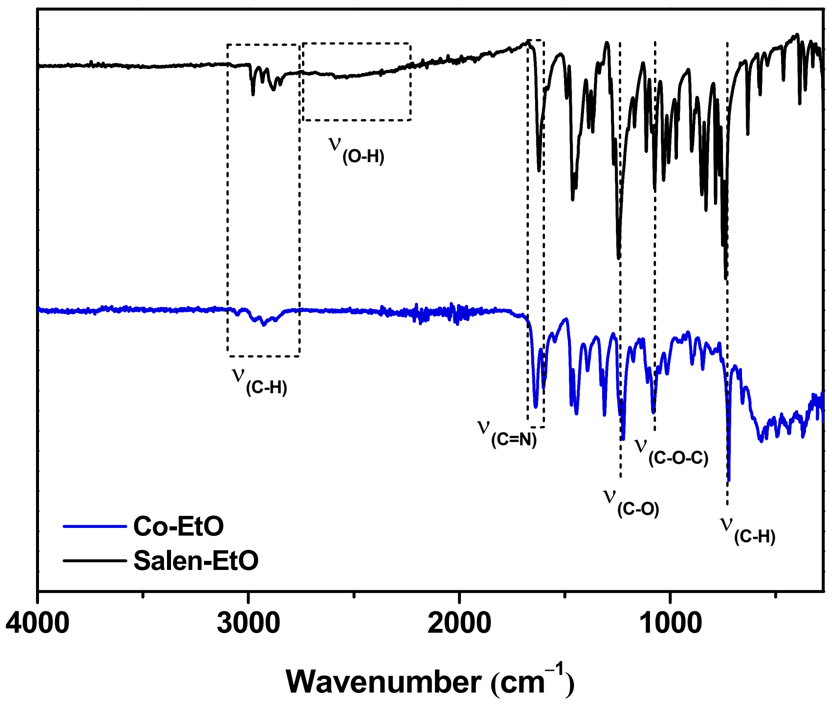

Figure S7. FTIR spectra for **Co-EtO** and ligand Salen-EtO.

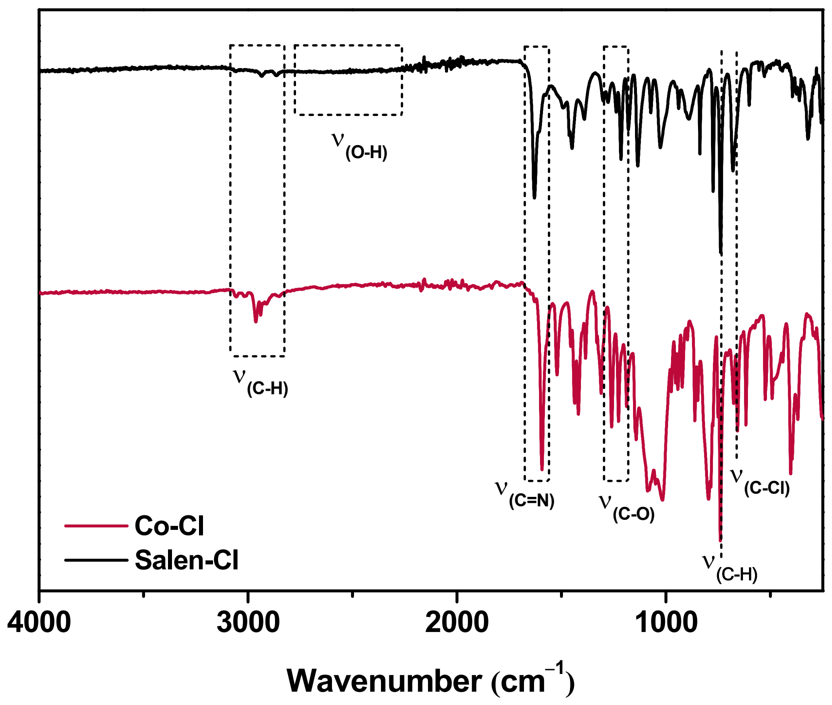

Figure S8. FTIR spectra for **Co-Cl** and ligand Salen-Cl.

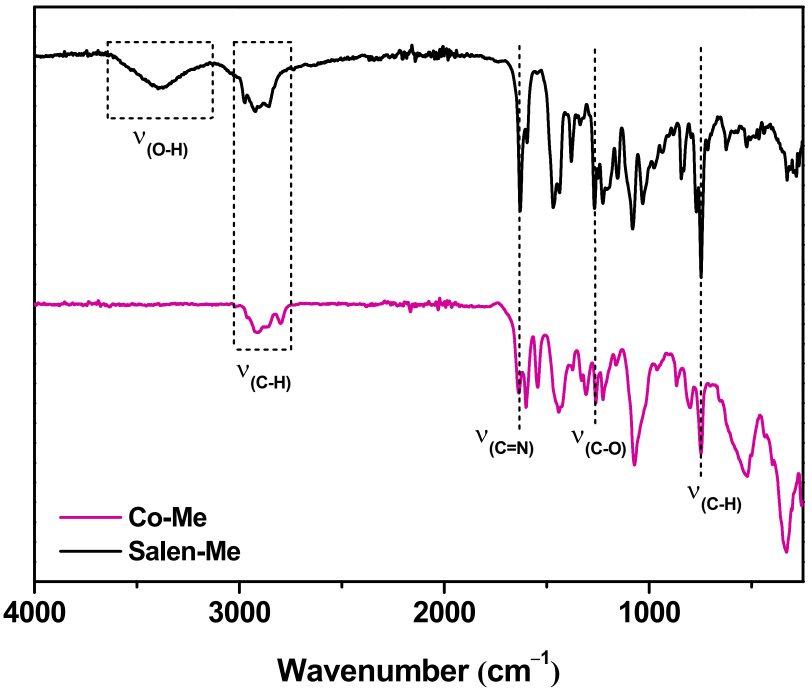

Figure S9. FTIR spectra for **Co-Me** and ligand Salen-Me.

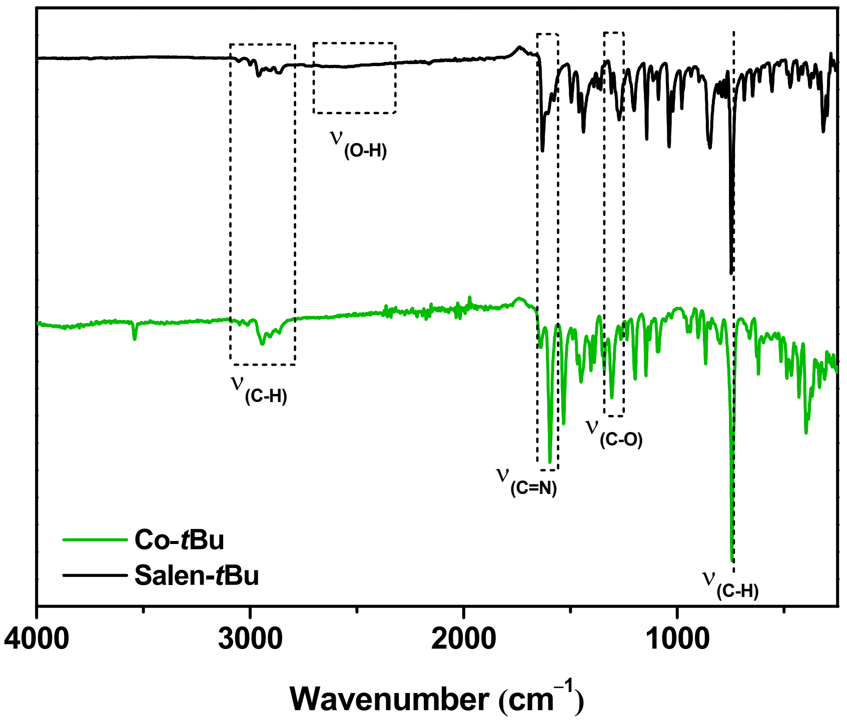

Figure S10. FTIR spectra for **Co-*t*Bu** and ligand Salen-*t*Bu.

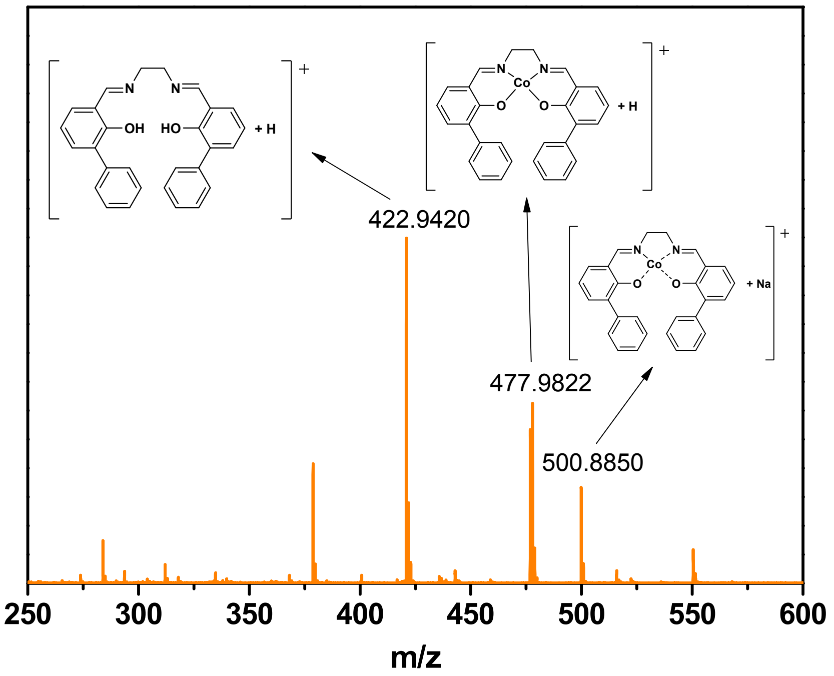

Figure S11. MALDI-TOF mass spectrum of the **Co-Ph** in CH_2Cl_2.

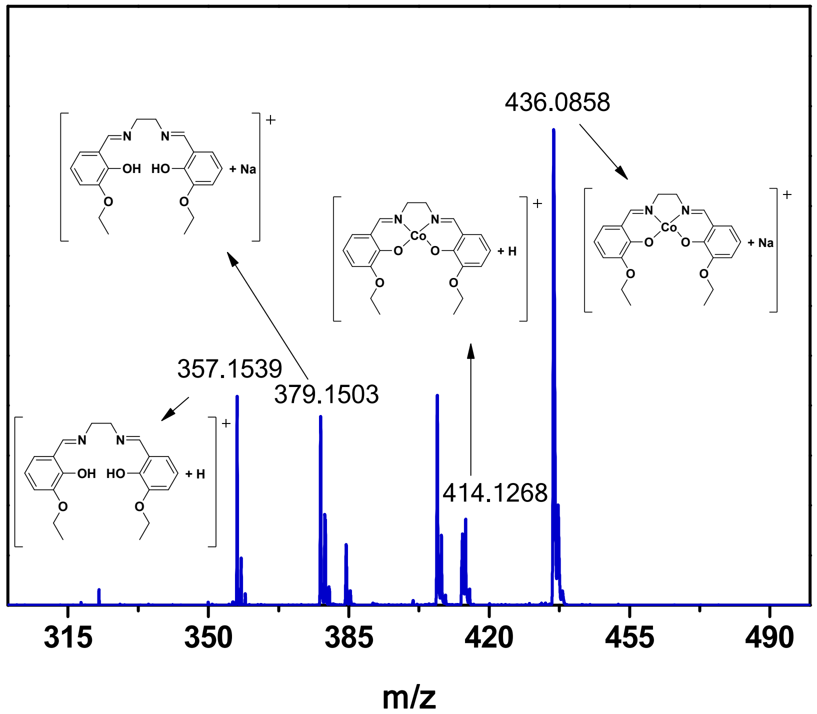

Figure S12. MALDI-TOF mass spectrum of the **Co-EtO** in CH_2Cl_2.

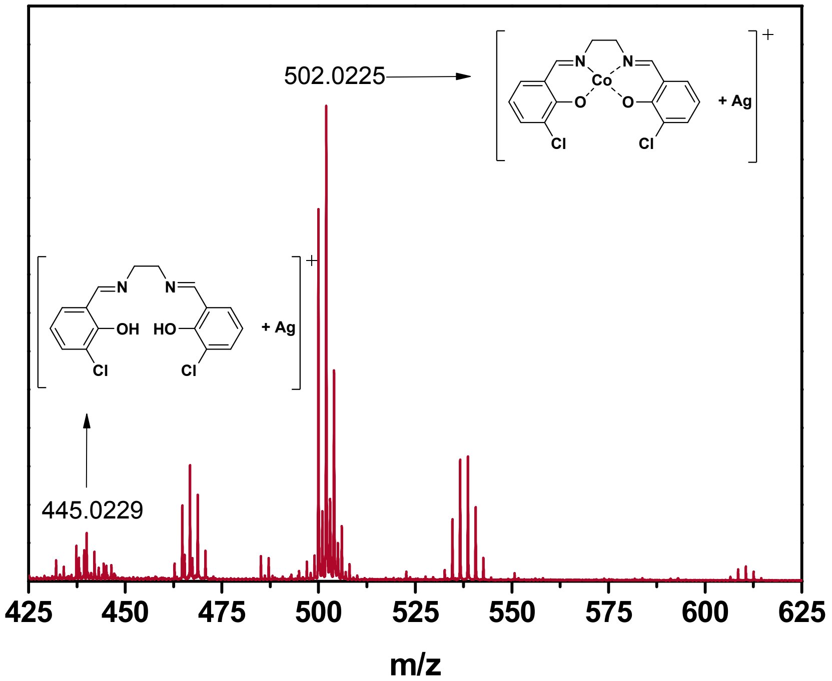

Figure S13. MALDI-TOF mass spectrum of the **Co-Cl** in CH_2Cl_2.

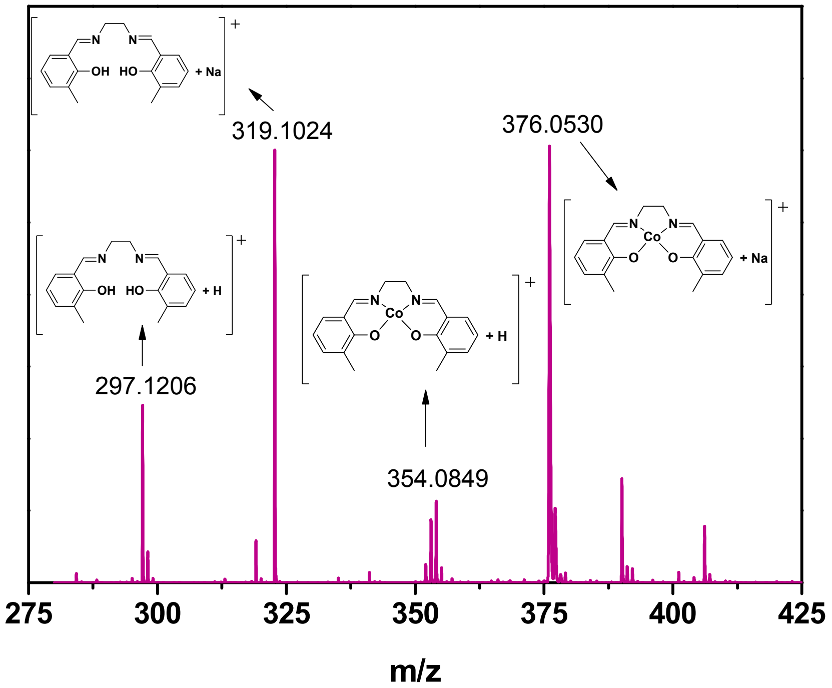

Figure S14. MALDI-TOF mass spectrum of the **Co-Me** in CH_2Cl_2.

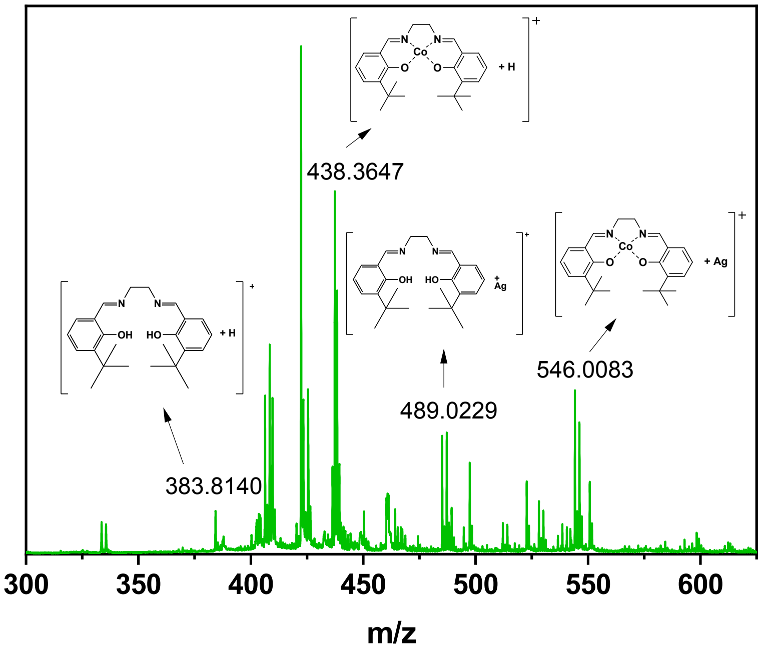

Figure S15. MALDI-TOF mass spectrum of the **Co-*t*Bu** in CH_2Cl_2.

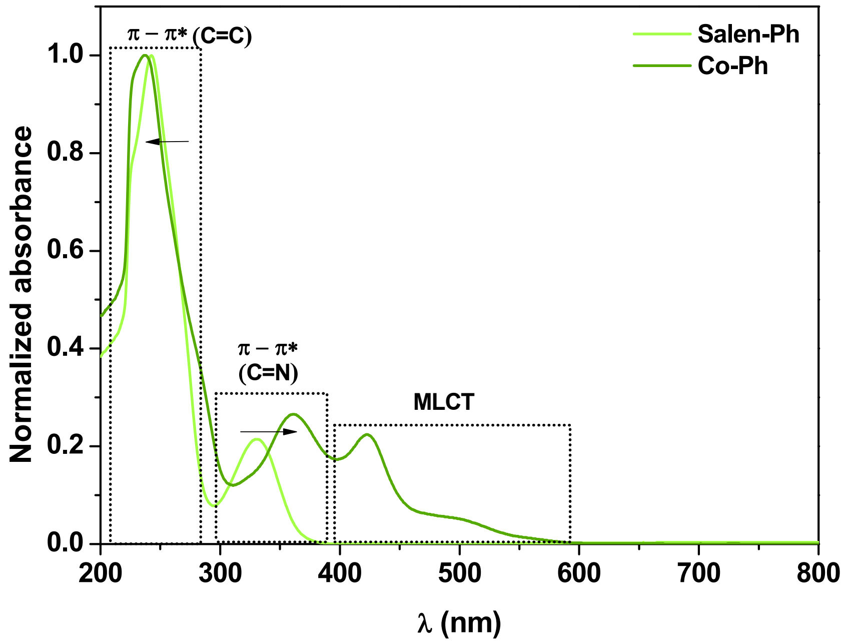

Figure S16. UV–Vis spectra for **Co-Ph** and Ligand Salen-Ph in CH_2Cl_2 at 25°C; [Co] = 1×10^{-5} mol L^{-1}.

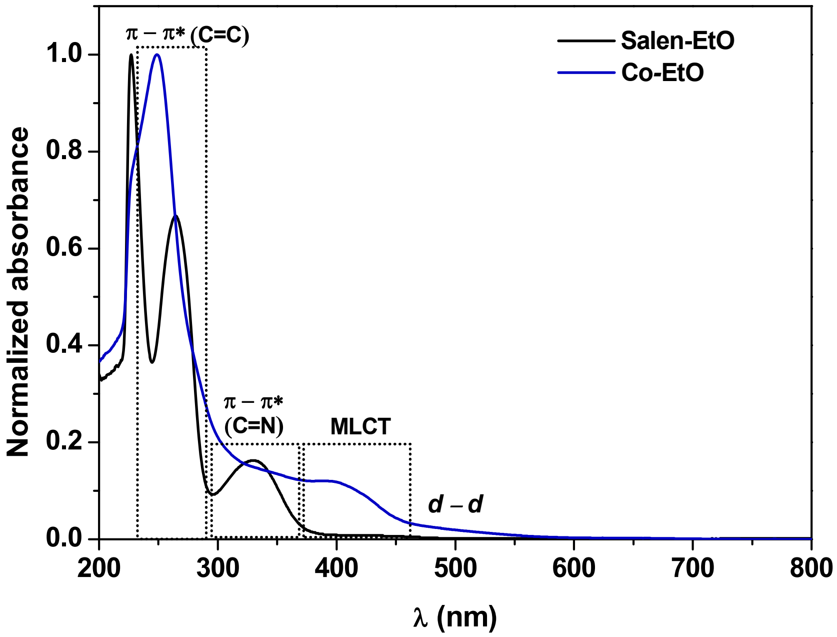

Figure S17. UV–Vis spectra for **Co-EtO** and Ligand Salen-EtO in CH_2Cl_2 at 25°C; [Co] = 1×10^{-5} mol L^{-1}.

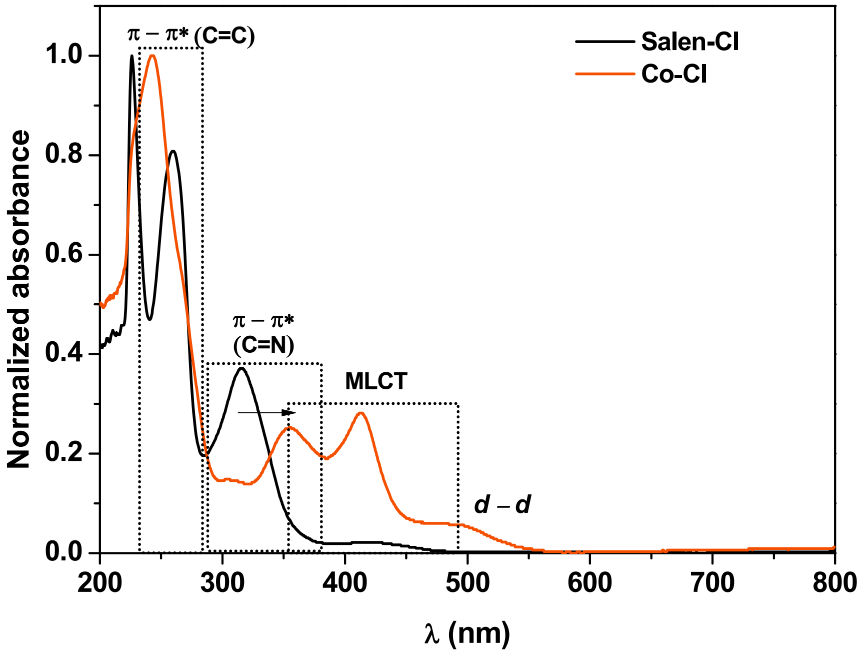

Figure S18. UV–Vis spectra for **Co-Cl** and Ligand Salen-Cl in CH_2Cl_2 at 25°C; [Co] = 1×10^{-5} mol L^{-1}.

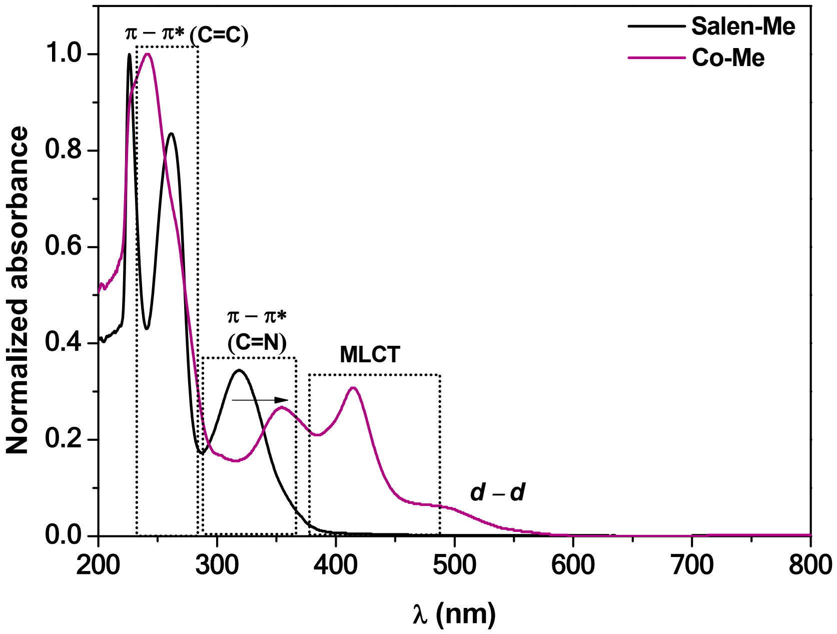

Figure S19. UV–Vis spectra for **Co-Me** and Ligand Salen-Me in CH_2Cl_2 at 25°C; [Co] = 1×10^{-5} mol L^{-1}.

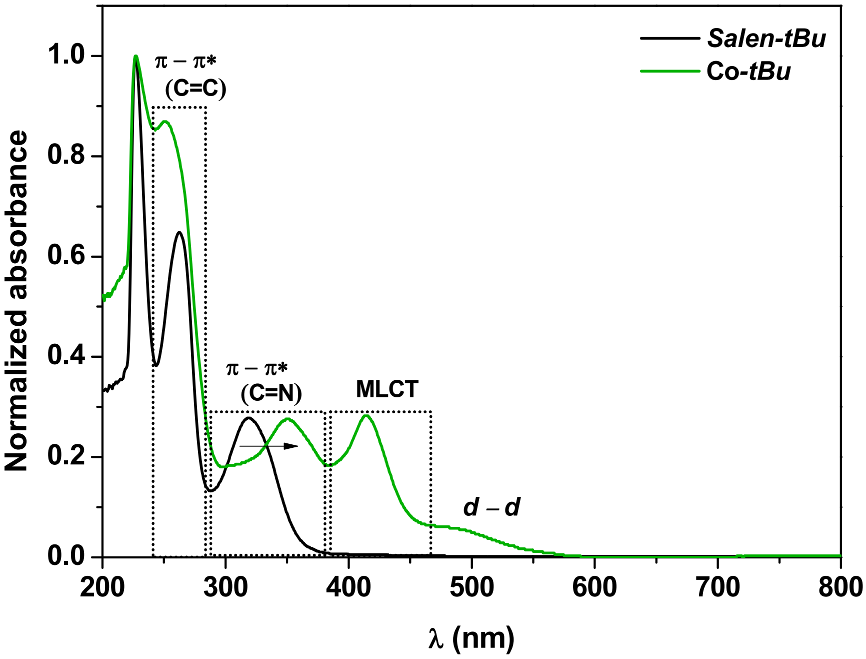

Figure S20. UV–Vis spectra for **Co-*t*Bu** and Ligand Salen-*t*Bu in CH_2Cl_2 at 25°C; [Co] = 1×10^{-5} mol L^{-1}.

Table S1. Geometric parameters for Co(II) complexes with singlet multiplicity.

Geometric parameter	Co-Ph	Co-EtO	Co-Cl	Co-Me	Co-*t*Bu
**Bond lengths (Å)**					
N(1)-Co	2.0404(7)	2.0282(6)	2.0650(6)	2.0296(8)	2.0363(8)
O(1)-Co	1.9221(3)	1.9136(0)	1.9234(7)	1.9206(6)	1.9211(7)
N(2)-Co	2.0387(0)	2.0302(9)	2.0581(2)	2.0335(8)	2.0394(8)
O(2)-Co	1.9239(6)	1.9110(0)	1.9242(9)	1.9194(7)	1.9245(6)
**Bond angles (º)**					
N(1)-Co-O(1)	89.38(9)	92.06(4)	88.92(9)	92.14(1)	89.72(6)
N(2)-Co-O(1)	164.97(0)	145.13(0)	147.82(5)	145.02(8)	158.38(7)
O(1)-Co-O(2)	101.92(9)	110.47(3)	113.15(7)	111.74(4)	106.19(9)
N(1)-Co-N(2)	81.26(0)	81.79(0)	80.07(4)	81.58(1)	81.08(1)
N(1)-Co-O(2)	165.03(0)	146.20(9)	150.15(8)	144.14(8)	155.20(5)
N(2)-Co-O(2)	89.46(8)	92.08(0)	89.47(1)	91.81(3)	89.45(2)

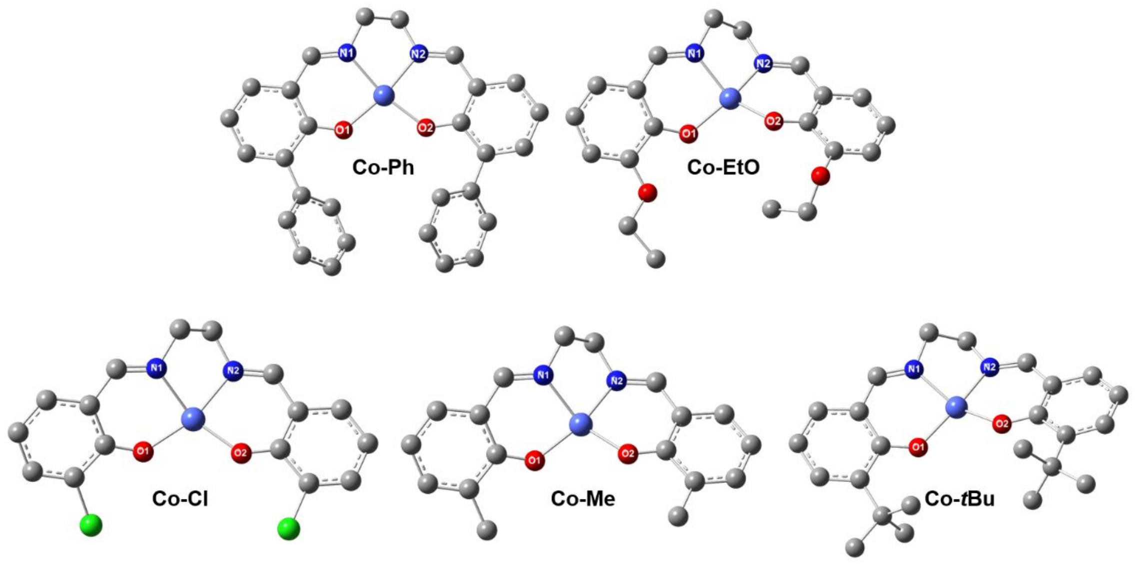

Figure S21. Optimized geometries of the Co(II) complexes.

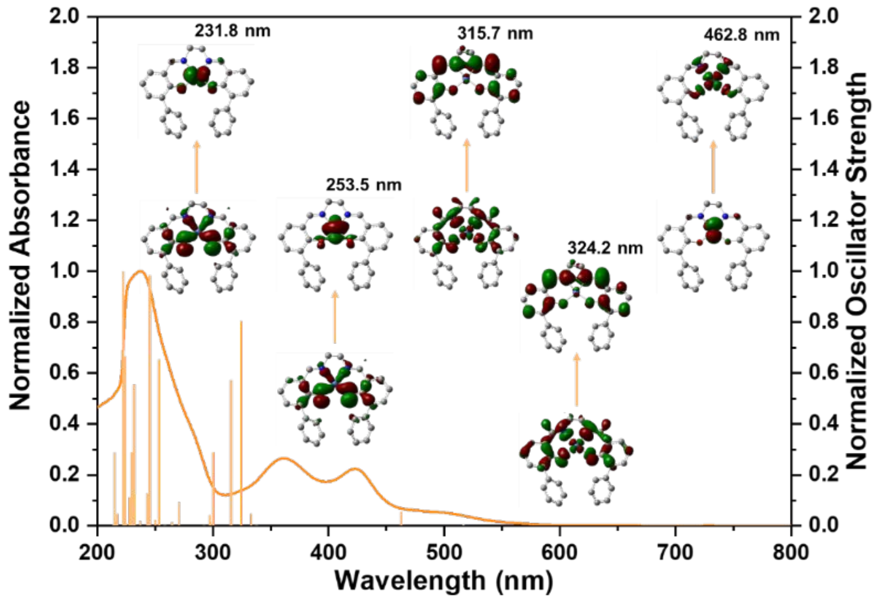

Figure S22. Normalized experimental (curve) and theoretical (vertical bars) electronic absorption spectrum for **Co-Ph** (quartet state), along with the corresponding NTO orbitals for selected main transitions in the simulated spectrum.

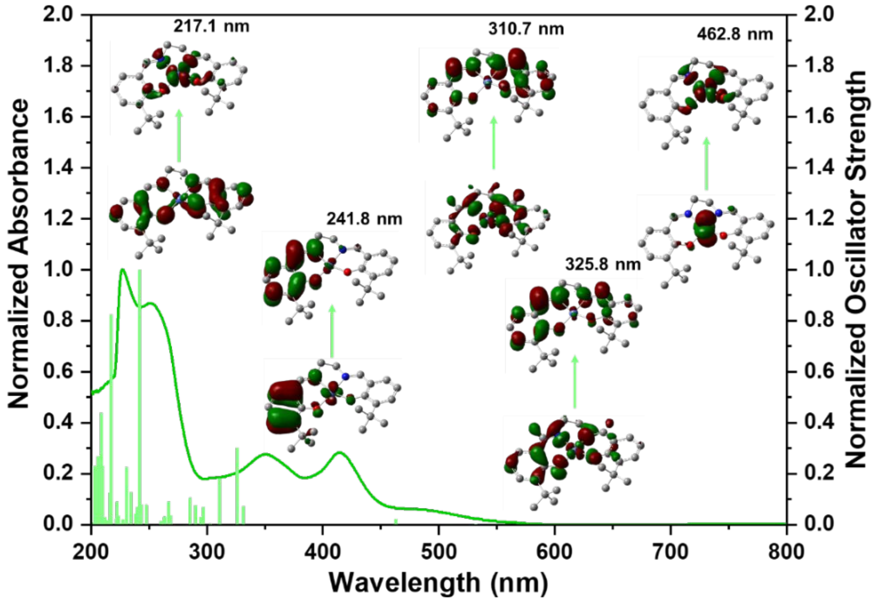

Figure S23. Normalized experimental (curve) and theoretical (vertical bars) electronic absorption spectrum for **Co-*t*Bu** (quartet state), along with the corresponding NTO orbitals for selected main transitions in the simulated spectrum.

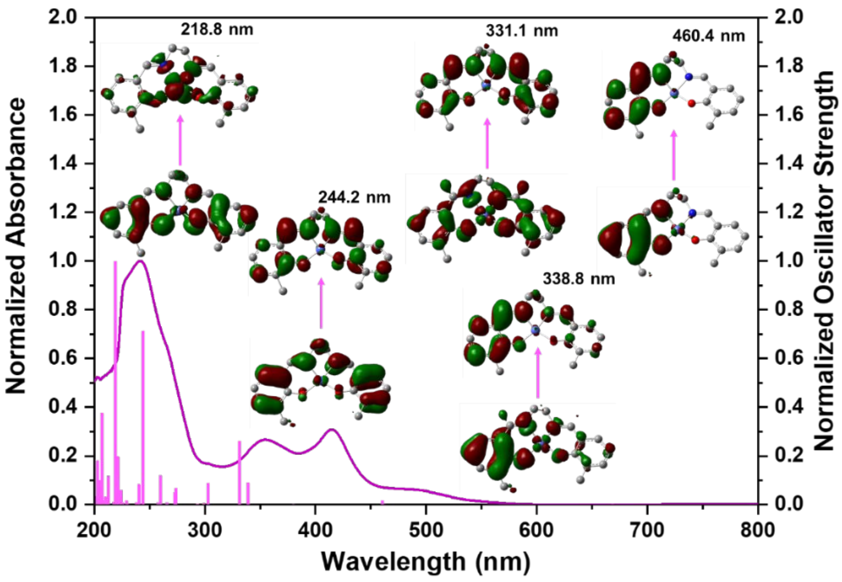

Figure S24. Normalized experimental (curve) and theoretical (vertical bars) electronic absorption spectrum for **Co-Me** (quartet state), along with the corresponding NTO orbitals for selected main transitions in the simulated spectrum.

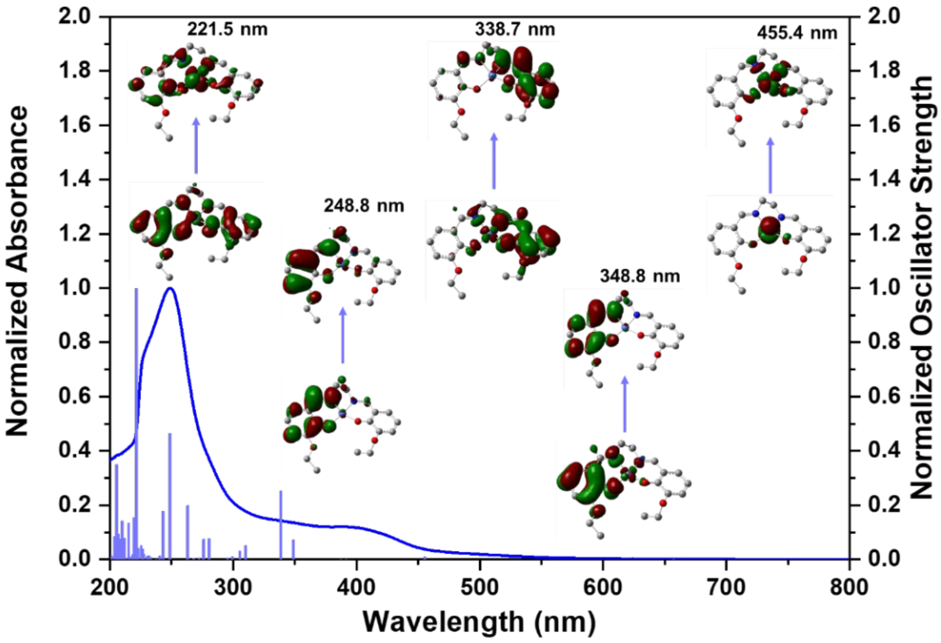

Figure S25. Normalized experimental (curve) and theoretical (vertical bars) electronic absorption spectrum for **Co-EtO** (quartet state), along with the corresponding NTO orbitals for selected main transitions in the simulated spectrum.

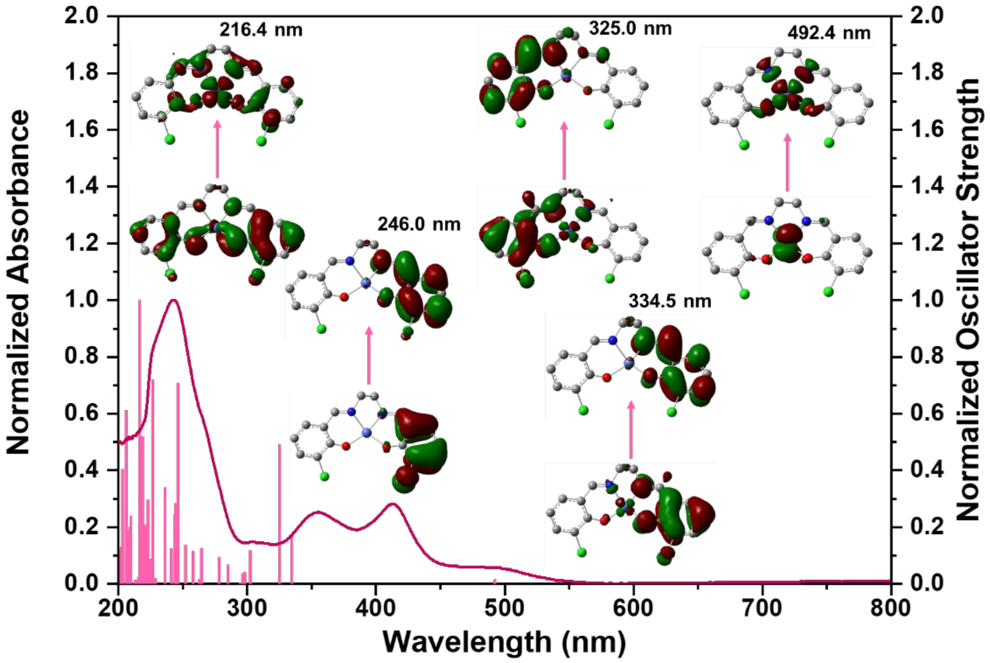

Figure S26. Normalized experimental (curve) and theoretical (vertical bars) electronic absorption spectrum for **Co-Cl** (quartet state), along with the corresponding NTO orbitals for selected main transitions in the simulated spectrum.

Table S2. Selected spin-allowed electronic transitions for **Co-*t*Bu**, determined using TD-DFT calculations on its quartet (S = 3/2) ground state. It includes the excitation energies (in eV), corresponding wavelengths (λ), oscillator strengths (f), and major orbital contributions for these transitions. Given the open-shell nature of the quartet state, orbitals are categorized as either alpha (α) spin orbitals or beta (β) spin orbitals. The three singly occupied α-spin orbitals (114a to 116a) are responsible for forming the quartet configuration. All listed transitions are spin-conserving, meaning they involve either an α→α or a β→β excitation. The orbital indices refer to the unrestricted Kohn-Sham orbitals of the quartet reference state, and the numerical value accompanying each orbital excitation indicates the weight of that configuration in the overall transition character.

Transition	Energy (eV)	*λ* (nm)	*f*	Major contributions
$Q_0 \rightarrow Q_5$	2.332	531.7	0.00099	106b→118b : 0.200051<br>106b→119b : 0.031834<br>106b→122b : 0.034287<br>107b→117b : 0.081122<br>109b→118b : 0.254792<br>109b→119b : 0.038810<br>109b→122b : 0.040210<br>113b→118b : 0.069386
$Q_0 \rightarrow Q_6$	2.679	462.8	0.00817	107b→118b : 0.496642<br>107b→119b : 0.074193<br>107b→120b : 0.048028<br>107b→122b : 0.082165<br>110b→118b : 0.026404<br>111b→118b : 0.073734
$Q_0 \rightarrow Q_7$	2.741	452.3	0.00003	112a→118a : 0.022083<br>115a→117a : 0.022035<br>115a→118a : 0.166903<br>116a→117a : 0.202954<br>116a→118a : 0.033089<br>112b→114b : 0.136302<br>112b→116b : 0.021638<br>113b→114b : 0.119805<br>113b→115b : 0.119171
$Q_0 \rightarrow Q_8$	2.756	449.9	0.00002	112a→117a : 0.025089<br>115a→117a : 0.179971<br>116a→117a : 0.049238<br>116a→118a : 0.189355<br>111b→115b : 0.032528<br>112b→115b : 0.204017<br>113b→114b : 0.093579<br>113b→115b : 0.064283<br>113b→116b : 0.023568

$Q_0 \rightarrow Q_9$	3.299	375.8	0.00003	112a→117a : 0.100380<br>113a→118a : 0.107410<br>115a→117a : 0.024669<br>115a→119a : 0.053933<br>116a→120a : 0.053814<br>110b→114b : 0.043735<br>111b→115b : 0.163506<br>112b→115b : 0.032974<br>112b→120b : 0.040378<br>113b→120b : 0.036773
$Q_0 \rightarrow Q_{10}$	3.304	375.3	0.00011	112a→118a : 0.097922<br>113a→117a : 0.112348<br>115a→118a : 0.021456<br>115a→120a : 0.056878<br>116a→119a : 0.053058<br>110b→114b : 0.103982<br>110b→115b : 0.056071<br>110b→116b : 0.029840<br>111b→114b : 0.022506<br>112b→119b : 0.026464<br>112b→120b : 0.022366<br>113b→119b : 0.054100
$Q_0 \rightarrow Q_{11}$	3.742	331.3	0.02721	115a→118a : 0.195574<br>116a→117a : 0.215888<br>112b→114b : 0.227940<br>112b→115b : 0.028608<br>113b→114b : 0.091223<br>113b→115b : 0.139981
$Q_0 \rightarrow Q_{12}$	3.806	325.8	0.11440	111a→118a : 0.041663<br>114a→117a : 0.187223<br>115a→117a : 0.256445<br>116a→118a : 0.068706<br>112b→115b : 0.120322<br>113b→114b : 0.175872<br>113b→115b : 0.036696
$Q_0 \rightarrow Q_{13}$	3.990	310.7	0.06913	111a→118a : 0.075045<br>114a→117a : 0.435569<br>116a→118a : 0.120448<br>112b→115b : 0.031105<br>113b→114b : 0.139980<br>113b→116b : 0.020331
$Q_0 \rightarrow Q_{14}$	4.095	302.7	0.00129	111a→117a : 0.181162<br>114a→118a : 0.544376<br>114a→120a : 0.020143<br>115a→118a : 0.051745<br>116a→117a : 0.040761
$Q_0 \rightarrow Q_{15}$	4.177	296.9	0.02610	112a→118a : 0.021420<br>113a→117a : 0.035535<br>115a→120a : 0.035273<br>116a→119a : 0.052960

				111b→115b : 0.063000<br>111b→116b : 0.022049<br>112b→114b : 0.091725<br>112b→116b : 0.133411<br>112b→117b : 0.045153<br>112b→120b : 0.051958<br>113b→115b : 0.031002<br>113b→116b : 0.042347<br>113b→118b : 0.022936<br>113b→120b : 0.021660
$Q_0 \rightarrow Q_{16}$	4.211	294.5	0.01104	112a→117a : 0.041644<br>113a→118a : 0.050872<br>115a→119a : 0.072264<br>115a→120a : 0.035307<br>116a→119a : 0.022244<br>116a→120a : 0.086003<br>110b→114b : 0.074716<br>112b→118b : 0.033223<br>112b→119b : 0.057718<br>113b→116b : 0.036205<br>113b→117b : 0.070194<br>113b→119b : 0.076806
$Q_0 \rightarrow Q_{17}$	4.276	290.0	0.02905	115a→119a : 0.027443<br>115a→120a : 0.028112<br>116a→119a : 0.043581<br>116a→120a : 0.021736<br>111b→114b : 0.027937<br>111b→115b : 0.027351<br>112b→114b : 0.103570<br>112b→115b : 0.037568<br>112b→116b : 0.131309<br>112b→117b : 0.027389<br>112b→118b : 0.024395<br>112b→120b : 0.033850<br>113b→114b : 0.034812<br>113b→116b : 0.128523<br>113b→118b : 0.021017
$Q_0 \rightarrow Q_{18}$	4.342	285.5	0.03972	109b→114b : 0.025423<br>112b→114b : 0.036046<br>112b→115b : 0.061987<br>112b→116b : 0.196673<br>113b→115b : 0.064453<br>113b→116b : 0.324042
$Q_0 \rightarrow Q_{19}$	4.615	268.6	0.01357	98b→117b : 0.023385<br>109b→117b : 0.036893<br>111b→115b : 0.031369<br>111b→117b : 0.021365<br>112b→117b : 0.267529<br>113b→117b : 0.371837
$Q_0 \rightarrow Q_{20}$	4.647	266.8	0.03425	115a→120a : 0.032136

				116a→119a : 0.029598<br>97b→117b : 0.022551<br>112b→117b : 0.393755<br>113b→117b : 0.251315
$Q_0 \rightarrow Q_{21}$	4.709	263.3	0.01177	106b→114b : 0.031148<br>108b→114b : 0.349095<br>109b→114b : 0.152748<br>109b→115b : 0.150742<br>112b→116b : 0.057168
$Q_0 \rightarrow Q_{22}$	4.765	260.2	0.00466	100b→114b : 0.020723<br>106b→114b : 0.089014<br>106b→116b : 0.033518<br>108b→115b : 0.179410<br>109b→114b : 0.190618<br>109b→115b : 0.180551<br>112b→115b : 0.027138<br>113b→115b : 0.024065
$Q_0 \rightarrow Q_{23}$	5.009	247.5	0.02941	112a→118a : 0.039654<br>113a→117a : 0.048552<br>108b→114b : 0.147149<br>108b→116b : 0.243043<br>109b→114b : 0.025734<br>109b→115b : 0.048961<br>110b→115b : 0.028174<br>111b→115b : 0.047807<br>112b→114b : 0.022264
$Q_0 \rightarrow Q_{24}$	5.101	243.0	0.02988	112a→117a : 0.076547<br>112a→122a : 0.020457<br>113a→118a : 0.079275<br>106b→114b : 0.036301<br>106b→116b : 0.024744<br>107b→115b : 0.068683<br>109b→116b : 0.047055<br>110b→114b : 0.069679<br>110b→115b : 0.023517<br>111b→115b : 0.032499<br>113b→119b : 0.037557<br>113b→120b : 0.022650
$Q_0 \rightarrow Q_{25}$	5.129	241.8	0.37893	112a→118a : 0.076061<br>113a→117a : 0.148518<br>116a→119a : 0.026898<br>108b→116b : 0.055854<br>109b→115b : 0.102282<br>110b→114b : 0.028258<br>111b→114b : 0.055133<br>111b→115b : 0.105985<br>112b→117b : 0.020530
$Q_0 \rightarrow Q_{26}$	5.171	239.8	0.02596	102a→117a : 0.027411<br>102a→118a : 0.020885<br>116a→120a : 0.025878

				96b→115b : 0.020669<br>107b→115b : 0.074245<br>110b→114b : 0.037591<br>110b→116b : 0.022111<br>111b→120b : 0.039966<br>111b→122b : 0.020995<br>111b→124b : 0.021508
$Q_0 \rightarrow Q_{27}$	5.198	238.5	0.01621	102a→117a : 0.028956<br>113a→122a : 0.024556<br>116a→120a : 0.021806<br>96b→114b : 0.020318<br>107b→114b : 0.037590<br>107b→116b : 0.020853<br>108b→116b : 0.036445<br>109b→115b : 0.032231<br>110b→119b : 0.033782<br>110b→122b : 0.029171<br>110b→124b : 0.021759
$Q_0 \rightarrow Q_{28}$	5.292	234.3	0.04779	112a→117a : 0.037870<br>113a→118a : 0.040789<br>100b→116b : 0.025341<br>106b→114b : 0.073354<br>106b→116b : 0.090187<br>108b→115b : 0.064687<br>109b→116b : 0.250603<br>110b→114b : 0.044973<br>111b→115b : 0.043114<br>112b→118b : 0.026961
$Q_0 \rightarrow Q_{29}$	5.383	230.3	0.08509	99b→117b : 0.042455<br>101b→117b : 0.044916<br>108b→117b : 0.733636
$Q_0 \rightarrow Q_{30}$	5.453	227.4	0.00738	110a→117a : 0.149180<br>111a→117a : 0.035074<br>111a→118a : 0.225796<br>115a→117a : 0.039198<br>115a→122a : 0.058683<br>116a→118a : 0.023131<br>106b→117b : 0.042654<br>107b→115b : 0.041124<br>109b→117b : 0.027702
$Q_0 \rightarrow Q_{31}$	5.522	224.5	0.00204	110a→118a : 0.163076<br>111a→117a : 0.317443<br>111a→119a : 0.020989<br>114a→118a : 0.027996<br>115a→118a : 0.040332<br>106b→117b : 0.056738<br>109b→117b : 0.044264
$Q_0 \rightarrow Q_{32}$	5.548	223.5	0.01282	110a→117a : 0.021860<br>110a→118a : 0.023242<br>111a→117a : 0.036259

				111a→118a : 0.048377<br>100b→117b : 0.041420<br>106b→117b : 0.231884<br>109b→117b : 0.208317<br>112b→118b : 0.073636
$Q_0 \rightarrow Q_{33}$	5.574	222.4	0.03425	111a→117a : 0.032274<br>111a→118a : 0.023144<br>116a→117a : 0.027253<br>116a→122a : 0.039159<br>106b→115b : 0.059804<br>107b→115b : 0.046389<br>108b→116b : 0.037653<br>112b→118b : 0.029108<br>112b→120b : 0.022102<br>112b→124b : 0.034179<br>113b→118b : 0.147223<br>113b→119b : 0.024830<br>113b→120b : 0.042961
$Q_0 \rightarrow Q_{34}$	5.589	221.8	0.00913	110a→117a : 0.051605<br>111a→118a : 0.089342<br>114a→122a : 0.020306<br>116a→124a : 0.029699<br>107b→114b : 0.032049<br>107b→115b : 0.096071<br>112b→115b : 0.041289<br>112b→118b : 0.059457<br>112b→119b : 0.042190<br>112b→120b : 0.024952<br>112b→122b : 0.020728<br>113b→118b : 0.034117<br>113b→119b : 0.025365<br>113b→124b : 0.038909
$Q_0 \rightarrow Q_{35}$	5.711	217.1	0.31240	115a→118a : 0.020164<br>115a→120a : 0.040620<br>116a→117a : 0.022685<br>116a→119a : 0.040544<br>106b→115b : 0.075699<br>108b→116b : 0.034702<br>109b→118b : 0.046232<br>113b→118b : 0.354338<br>113b→122b : 0.036855
$Q_0 \rightarrow Q_{36}$	5.736	216.2	0.04718	115a→118a : 0.058946<br>115a→124a : 0.022087<br>116a→117a : 0.068453<br>116a→122a : 0.048156<br>106b→115b : 0.202775<br>107b→114b : 0.026047<br>107b→115b : 0.020726<br>107b→116b : 0.020446<br>108b→115b : 0.043494

				108b→116b : 0.052563<br>108b→117b : 0.020275<br>112b→115b : 0.021897<br>113b→115b : 0.031270
$Q_0 \rightarrow Q_{37}$	5.793	214.0	0.00622	112a→119a : 0.031212<br>113a→120a : 0.036908<br>115a→117a : 0.053784<br>116a→118a : 0.041356<br>107b→114b : 0.036782<br>107b→115b : 0.102509<br>109b→114b : 0.022217<br>111b→114b : 0.034725<br>111b→118b : 0.036286<br>112b→118b : 0.130851
$Q_0 \rightarrow Q_{38}$	5.849	212.0	0.01056	112a→117a : 0.029648<br>112a→119a : 0.033752<br>113a→119a : 0.025316<br>114a→117a : 0.045494<br>115a→117a : 0.155881<br>116a→118a : 0.159109<br>107b→114b : 0.022914<br>111b→118b : 0.044837<br>111b→120b : 0.057702<br>112b→118b : 0.035416
$Q_0 \rightarrow Q_{39}$	5.865	211.4	0.00578	106b→114b : 0.125822<br>106b→116b : 0.109561<br>108b→114b : 0.024701<br>108b→115b : 0.091604<br>108b→116b : 0.036924<br>109b→114b : 0.023245<br>112b→115b : 0.075769<br>112b→118b : 0.021525<br>113b→114b : 0.048640<br>113b→116b : 0.052661
$Q_0 \rightarrow Q_{40}$	5.874	211.1	0.00919	112a→120a : 0.111502<br>113a→119a : 0.104333<br>115a→117a : 0.036300<br>116a→118a : 0.041933<br>110b→117b : 0.024709<br>110b→118b : 0.062189<br>110b→119b : 0.124454<br>111b→117b : 0.027185<br>111b→120b : 0.065150
$Q_0 \rightarrow Q_{41}$	5.913	209.7	0.08722	112a→119a : 0.053686<br>113a→120a : 0.046372<br>115a→122a : 0.022918<br>116a→120a : 0.024856<br>116a→124a : 0.028167<br>110b→114b : 0.027392<br>110b→117b : 0.034577

				110b→119b : 0.052077<br>111b→120b : 0.035383<br>112b→118b : 0.168390<br>112b→122b : 0.041063
$Q_0 \rightarrow Q_{42}$	5.942	208.6	0.16600	112a→118a : 0.036524<br>114a→118a : 0.042295<br>115a→118a : 0.099034<br>115a→120a : 0.038573<br>116a→117a : 0.137154<br>116a→119a : 0.074526<br>110b→114b : 0.046339<br>112b→120b : 0.048804<br>113b→119b : 0.080741
$Q_0 \rightarrow Q_{43}$	5.976	207.5	0.02078	114a→117a : 0.027895<br>115a→117a : 0.050765<br>116a→118a : 0.092455<br>107b→115b : 0.043287<br>109b→114b : 0.042457<br>111b→114b : 0.038882<br>112b→115b : 0.040532<br>112b→122b : 0.027426<br>113b→116b : 0.023002<br>113b→120b : 0.023233
$Q_0 \rightarrow Q_{44}$	6.017	206.1	0.10119	115a→120a : 0.020416<br>97b→114b : 0.035305<br>99b→114b : 0.038116<br>101b→114b : 0.053348<br>109b→115b : 0.049608<br>112b→114b : 0.081114<br>112b→116b : 0.087644<br>112b→119b : 0.023437<br>112b→120b : 0.026358<br>113b→115b : 0.092206<br>113b→119b : 0.059911
$Q_0 \rightarrow Q_{45}$	6.071	204.2	0.03892	114a→118a : 0.024359<br>115a→118a : 0.084458<br>115a→120a : 0.041866<br>115a→124a : 0.045355<br>116a→117a : 0.039465<br>116a→122a : 0.059002<br>101b→114b : 0.027206<br>110b→114b : 0.031719<br>112b→114b : 0.035263<br>112b→116b : 0.041842<br>113b→115b : 0.026057<br>113b→119b : 0.084623<br>113b→122b : 0.039814
$Q_0 \rightarrow Q_{46}$	6.093	203.5	0.01844	116a→122a : 0.027376<br>97b→114b : 0.034397<br>106b→115b : 0.049114

				107b→114b : 0.106737<br>107b→115b : 0.043359<br>107b→116b : 0.033659<br>109b→115b : 0.044007<br>110b→115b : 0.033646<br>112b→114b : 0.045925<br>112b→116b : 0.078145<br>113b→115b : 0.107074
$Q_0 \rightarrow Q_{47}$	6.100	203.3	0.08746	112a→117a : 0.053500<br>115a→119a : 0.086229<br>115a→122a : 0.050932<br>116a→118a : 0.049694<br>116a→120a : 0.126780<br>116a→124a : 0.048078<br>110b→114b : 0.028956<br>111b→115b : 0.026282<br>112b→119b : 0.056531<br>112b→120b : 0.084943<br>113b→120b : 0.081543
$Q_0 \rightarrow Q_{48}$	6.128	202.3	0.00257	115a→122a : 0.044141<br>116a→124a : 0.040393<br>99b→115b : 0.023828<br>101b→115b : 0.041071<br>106b→114b : 0.037085<br>109b→114b : 0.030379<br>109b→116b : 0.089889<br>110b→114b : 0.040513<br>110b→116b : 0.046229<br>111b→114b : 0.037332<br>112b→115b : 0.087388<br>113b→114b : 0.044765<br>113b→115b : 0.021294<br>113b→116b : 0.029684

Table S3. Selected spin-allowed electronic transitions for **Co-Ph**, determined using TD-DFT calculations on its quartet (S = 3/2) ground state. It includes the excitation energies (in eV), corresponding wavelengths (λ), oscillator strengths (f), and major orbital contributions for these transitions. Given the open-shell nature of the quartet state, orbitals are categorized as either alpha (α) spin orbitals or beta (β) spin orbitals. The three singly occupied α-spin orbitals (114a to 116a) are responsible for forming the quartet configuration. All listed transitions are spin-conserving, meaning they involve either an α→α or a β→β excitation. The orbital indices refer to the unrestricted Kohn-Sham orbitals of the quartet reference state, and the numerical value accompanying each

orbital excitation indicates the weight of that configuration in the overall transition character.

Transition	Energy (eV)	λ (nm)	f	Major contributions
$Q_0 \rightarrow Q_5$	2.401	516.5	0.00110	110b→128b : 0.223596<br>110b→131b : 0.024551<br>111b→125b : 0.052119<br>114b→125b : 0.034270<br>115b→128b : 0.349884<br>115b→131b : 0.036201<br>116b→128b : 0.101291
$Q_0 \rightarrow Q_6$	2.679	462.8	0.01272	98b→128b : 0.021210<br>111b→128b : 0.457891<br>111b→131b : 0.048809<br>114b→128b : 0.285899<br>114b→131b : 0.030011
$Q_0 \rightarrow Q_7$	2.734	453.5	0.000006	120a→126a : 0.042941<br>121a→125a : 0.027749<br>123a→126a : 0.155185<br>124a→125a : 0.249357<br>118b→123b : 0.028650<br>119b→122b : 0.030172<br>120b→122b : 0.059290<br>120b→123b : 0.062463<br>121b→122b : 0.141473<br>121b→123b : 0.084333
$Q_0 \rightarrow Q_8$	2.745	451.6	0.000007	120a→125a : 0.047243<br>121a→126a : 0.021615<br>123a→125a : 0.175903<br>124a→126a : 0.221776<br>118b→122b : 0.037178<br>120b→122b : 0.109451<br>120b→123b : 0.113146<br>121b→122b : 0.058777<br>121b→123b : 0.065043
$Q_0 \rightarrow Q_9$	3.039	407.9	0.00014	115a→125a : 0.026544<br>115a→126a : 0.020329<br>120a→125a : 0.022589<br>120a→126a : 0.028744<br>121a→125a : 0.052323<br>121a→126a : 0.038293<br>123a→127a : 0.058444<br>124a→127a : 0.060695<br>113b→122b : 0.020462<br>119b→122b : 0.076969<br>119b→123b : 0.050621<br>121b→126b : 0.112133
$Q_0 \rightarrow Q_{10}$	3.054	406.0	0.00015	116a→125a : 0.031177<br>116a→126a : 0.025145

				120a→125a : 0.031459<br>120a→126a : 0.023923<br>121a→125a : 0.034618<br>121a→126a : 0.049350<br>123a→128a : 0.069755<br>124a→128a : 0.050172<br>118b→122b : 0.059690<br>118b→123b : 0.054351<br>120b→126b : 0.024746<br>120b→127b : 0.099542
$Q_0 \rightarrow Q_{11}$	3.669	337.9	0.00038	115a→125a : 0.023827<br>116a→126a : 0.031638<br>118a→130a : 0.063183<br>119a→129a : 0.071563<br>120a→127a : 0.021141<br>121a→128a : 0.031163<br>113b→122b : 0.028334<br>114b→123b : 0.022792<br>116b→130b : 0.053264<br>117b→129b : 0.089091<br>118b→127b : 0.042512<br>119b→126b : 0.021585
$Q_0 \rightarrow Q_{12}$	3.679	337.0	0.00028	115a→126a : 0.030578<br>115a→127a : 0.023549<br>116a→125a : 0.025564<br>118a→129a : 0.071235<br>119a→130a : 0.064304<br>121a→127a : 0.037436<br>123a→134a : 0.020684<br>113b→123b : 0.026117<br>114b→122b : 0.029696<br>116b→129b : 0.059639<br>117b→130b : 0.077609<br>119b→126b : 0.027345
$Q_0 \rightarrow Q_{13}$	3.722	333.1	0.01076	123a→126a : 0.162157<br>124a→125a : 0.249784<br>118b→123b : 0.034242<br>119b→122b : 0.023760<br>120b→122b : 0.089743<br>120b→123b : 0.110525<br>121b→122b : 0.129676<br>121b→123b : 0.086465
$Q_0 \rightarrow Q_{14}$	3.825	324.2	0.18982	117a→126a : 0.032649<br>119a→125a : 0.029342<br>122a→125a : 0.239142<br>123a→125a : 0.163241<br>124a→126a : 0.101131<br>120b→122b : 0.055094<br>120b→123b : 0.091938<br>121b→122b : 0.053014

				121b→123b : 0.087442
$Q_0 \rightarrow Q_{15}$	3.927	315.7	0.13497	117a→126a : 0.056583<br>119a→125a : 0.066880<br>120a→125a : 0.073619<br>122a→125a : 0.330040<br>122a→128a : 0.021078<br>124a→126a : 0.100957<br>120b→122b : 0.025042<br>120b→123b : 0.060628<br>121b→122b : 0.025194<br>121b→123b : 0.059991
$Q_0 \rightarrow Q_{16}$	4.062	305.3	0.00020	117a→125a : 0.134644<br>119a→126a : 0.079039<br>120a→126a : 0.060563<br>122a→126a : 0.459577<br>122a→127a : 0.042885
$Q_0 \rightarrow Q_{17}$	4.129	300.3	0.06790	123a→125a : 0.033496<br>124a→126a : 0.031029<br>118b→122b : 0.020475<br>119b→124b : 0.082826<br>120b→122b : 0.022139<br>120b→124b : 0.210678<br>121b→124b : 0.280381
$Q_0 \rightarrow Q_{18}$	4.164	297.7	0.00318	121a→125a : 0.036944<br>122a→126a : 0.047512<br>123a→127a : 0.035904<br>123a→133a : 0.047951<br>124a→128a : 0.038403<br>124a→131a : 0.064434<br>118b→124b : 0.025942<br>119b→122b : 0.037560<br>120b→124b : 0.044119<br>120b→125b : 0.030970<br>120b→127b : 0.022231<br>121b→125b : 0.053007<br>121b→126b : 0.059119<br>121b→131b : 0.039095<br>121b→132b : 0.028521
$Q_0 \rightarrow Q_{19}$	4.172	297.2	0.00985	120a→125a : 0.023987<br>121a→126a : 0.031539<br>123a→128a : 0.051184<br>123a→131a : 0.052455<br>124a→127a : 0.042006<br>124a→133a : 0.056844<br>119b→123b : 0.034812<br>120b→124b : 0.026064<br>120b→125b : 0.057514<br>120b→127b : 0.043680<br>120b→131b : 0.042817<br>120b→132b : 0.028027

				121b→124b : 0.062341<br>121b→125b : 0.032463<br>121b→126b : 0.022339
$Q_0 \rightarrow Q_{20}$	4.243	292.2	0.00061	103b→124b : 0.023351<br>118b→123b : 0.037681<br>118b→124b : 0.114499<br>119b→124b : 0.025827<br>120b→122b : 0.020467<br>120b→123b : 0.038611<br>120b→124b : 0.296297<br>121b→123b : 0.035509<br>121b→124b : 0.225757
$Q_0 \rightarrow Q_{21}$	4.544	272.9	0.00018	123a→128a : 0.023210<br>124a→127a : 0.031624<br>114b→122b : 0.023164<br>115b→125b : 0.022631<br>119b→125b : 0.089958<br>120b→125b : 0.216143<br>121b→125b : 0.309892
$Q_0 \rightarrow Q_{22}$	4.586	270.4	0.02199	123a→127a : 0.037215<br>124a→128a : 0.052143<br>103b→125b : 0.020844<br>118b→125b : 0.135780<br>119b→125b : 0.026814<br>120b→125b : 0.281468<br>121b→125b : 0.189793
$Q_0 \rightarrow Q_{23}$	4.685	264.6	0.00312	112b→123b : 0.224500<br>115b→122b : 0.356021<br>115b→126b : 0.029886<br>116b→122b : 0.177081
$Q_0 \rightarrow Q_{24}$	4.760	260.5	0.00002	118a→127a : 0.055542<br>119a→127a : 0.077701<br>121a→130a : 0.028336<br>123a→129a : 0.034915<br>124a→130a : 0.022375<br>116b→126b : 0.061023<br>117b→126b : 0.068778<br>117b→127b : 0.029069<br>119b→129b : 0.028943<br>119b→130b : 0.033771<br>121b→129b : 0.044171<br>121b→130b : 0.035837
$Q_0 \rightarrow Q_{25}$	4.767	260.1	0.00005	118a→128a : 0.056019<br>119a→128a : 0.065506<br>121a→129a : 0.027243<br>123a→129a : 0.022797<br>123a→130a : 0.027249<br>115b→127b : 0.025699<br>116b→123b : 0.039996<br>116b→127b : 0.046991

				117b→126b : 0.021415<br>117b→127b : 0.064479<br>118b→129b : 0.030001<br>118b→130b : 0.029913<br>120b→129b : 0.041338<br>120b→130b : 0.034463
$Q_0 \rightarrow Q_{26}$	4.788	258.9	0.00003	118a→129a : 0.026607<br>119a→130a : 0.025547<br>110b→123b : 0.043611<br>110b→124b : 0.023326<br>112b→122b : 0.109477<br>115b→123b : 0.190770<br>116b→123b : 0.087855<br>116b→129b : 0.023340<br>117b→130b : 0.034377
$Q_0 \rightarrow Q_{27}$	4.794	258.6	0.00035	118a→130a : 0.090653<br>119a→129a : 0.108735<br>120a→127a : 0.033217<br>121a→128a : 0.037147<br>115b→123b : 0.021848<br>116b→130b : 0.073091<br>117b→129b : 0.130477<br>118b→127b : 0.034359<br>119b→126b : 0.040500
$Q_0 \rightarrow Q_{28}$	4.808	257.9	0.00029	118a→129a : 0.086536<br>119a→130a : 0.075073<br>120a→128a : 0.033872<br>121a→127a : 0.028041<br>110b→123b : 0.020192<br>112b→122b : 0.046661<br>115b→123b : 0.075945<br>116b→123b : 0.023860<br>116b→129b : 0.069825<br>117b→130b : 0.087537<br>118b→127b : 0.026334
$Q_0 \rightarrow Q_{29}$	4.890	253.5	0.15403	120a→126a : 0.048670<br>121a→125a : 0.096533<br>123a→126a : 0.027763<br>124a→128a : 0.021896<br>112b→123b : 0.041022<br>112b→124b : 0.183068<br>118b→123b : 0.037382<br>119b→122b : 0.064216<br>120b→127b : 0.041242<br>121b→126b : 0.059514
$Q_0 \rightarrow Q_{30}$	4.960	250.0	0.00566	116a→125a : 0.021570<br>120a→125a : 0.081840<br>121a→126a : 0.111162<br>123a→125a : 0.021250<br>123a→128a : 0.024618

				124a→127a : 0.025854<br>110b→124b : 0.062520<br>114b→122b : 0.039815<br>115b→124b : 0.027790<br>116b→124b : 0.024321<br>118b→122b : 0.051925<br>118b→123b : 0.021368<br>119b→123b : 0.064126<br>120b→127b : 0.066168<br>121b→126b : 0.046153
$Q_0 \rightarrow Q_{31}$	5.059	245.1	0.09995	118a→128a : 0.022185<br>119a→127a : 0.035445<br>120a→129a : 0.022648<br>121a→130a : 0.032455<br>123a→129a : 0.053447<br>124a→130a : 0.030118<br>112b→123b : 0.026217<br>112b→124b : 0.107142<br>116b→126b : 0.022313<br>117b→124b : 0.023790<br>117b→127b : 0.033047<br>118b→129b : 0.031648<br>119b→130b : 0.029918<br>120b→129b : 0.023775<br>121b→129b : 0.034933<br>121b→130b : 0.024021
$Q_0 \rightarrow Q_{32}$	5.061	245.0	0.23192	121a→125a : 0.043042<br>123a→129a : 0.026615<br>112b→123b : 0.046954<br>112b→124b : 0.223668<br>115b→122b : 0.042235<br>118b→123b : 0.030624<br>119b→122b : 0.030816
$Q_0 \rightarrow Q_{33}$	5.074	244.3	0.00207	118a→127a : 0.035649<br>119a→128a : 0.039448<br>120a→130a : 0.024928<br>121a→129a : 0.048750<br>123a→130a : 0.053864<br>124a→129a : 0.044355<br>113b→129b : 0.020177<br>116b→127b : 0.037550<br>117b→126b : 0.032149<br>118b→130b : 0.032983<br>119b→129b : 0.028796<br>120b→129b : 0.032524<br>120b→130b : 0.034458
$Q_0 \rightarrow Q_{34}$	5.087	243.7	0.00343	108a→126a : 0.020810<br>115a→127a : 0.022128<br>121a→136a : 0.023228<br>124a→127a : 0.033108

				104b→123b : 0.021501<br>111b→122b : 0.067619<br>114b→122b : 0.030090<br>114b→126b : 0.024925
$Q_0 \rightarrow Q_{35}$	5.101	243.1	0.02949	105a→125a : 0.020909<br>108a→125a : 0.031296<br>113a→125a : 0.029754<br>120a→126a : 0.039458<br>120a→136a : 0.028655<br>121a→134a : 0.035866<br>102b→122b : 0.020935<br>104b→122b : 0.036076<br>112b→124b : 0.060954<br>114b→123b : 0.028009<br>114b→127b : 0.025377<br>119b→134b : 0.021744
$Q_0 \rightarrow Q_{36}$	5.230	237.0	0.00448	121a→126a : 0.024311<br>110b→123b : 0.063850<br>110b→124b : 0.259591<br>112b→122b : 0.031290<br>115b→124b : 0.155131<br>116b→124b : 0.114812<br>118b→122b : 0.021874<br>121b→126b : 0.021700
$Q_0 \rightarrow Q_{37}$	5.336	232.4	0.02924	118a→128a : 0.035322<br>119a→127a : 0.060695<br>120a→129a : 0.025196<br>121a→130a : 0.034301<br>123a→129a : 0.057826<br>124a→130a : 0.031369<br>116b→126b : 0.043782<br>117b→126b : 0.024575<br>117b→127b : 0.059855<br>118b→129b : 0.024059<br>119b→130b : 0.029875<br>120b→129b : 0.020980<br>121b→129b : 0.037835<br>121b→130b : 0.028247
$Q_0 \rightarrow Q_{38}$	5.350	231.8	0.13081	123a→127a : 0.032639<br>124a→128a : 0.022503<br>112b→123b : 0.022344<br>112b→125b : 0.426341<br>117b→125b : 0.025140<br>120b→127b : 0.049652<br>121b→126b : 0.045031
$Q_0 \rightarrow Q_{39}$	5.358	231.4	0.00370	118a→126a : 0.023625<br>118a→127a : 0.042565<br>118a→128a : 0.024278<br>119a→128a : 0.059955<br>120a→130a : 0.024918

				121a→129a : 0.049544<br>123a→130a : 0.055530<br>124a→129a : 0.047462<br>116b→127b : 0.058391<br>117b→126b : 0.056292<br>117b→127b : 0.025285<br>118b→130b : 0.033597<br>119b→129b : 0.028042<br>120b→129b : 0.039868<br>120b→130b : 0.038892
$Q_0 \rightarrow Q_{40}$	5.399	229.6	0.06816	123a→127a : 0.060537<br>124a→128a : 0.041897<br>112b→125b : 0.256499<br>114b→123b : 0.030755<br>120b→127b : 0.078036<br>121b→126b : 0.092918
$Q_0 \rightarrow Q_{41}$	5.438	228.0	0.02632	123a→131a : 0.034372<br>124a→136a : 0.028173<br>110b→125b : 0.126821<br>111b→122b : 0.073119<br>114b→122b : 0.048341<br>115b→125b : 0.042442<br>116b→125b : 0.039998<br>120b→122b : 0.024805<br>120b→127b : 0.025514<br>120b→128b : 0.022069
$Q_0 \rightarrow Q_{42}$	5.509	225.0	0.00199	106b→125b : 0.022747<br>110b→125b : 0.295565<br>115b→125b : 0.135986<br>116b→125b : 0.094695<br>120b→122b : 0.025873<br>120b→128b : 0.021782<br>121b→128b : 0.020943
$Q_0 \rightarrow Q_{43}$	5.543	223.7	0.15666	114a→125a : 0.124086<br>117a→126a : 0.178713<br>118a→126a : 0.034169<br>122a→131a : 0.021444<br>123a→125a : 0.094808<br>123a→128a : 0.059925<br>124a→126a : 0.063164<br>124a→127a : 0.040195<br>120b→127b : 0.038283<br>121b→126b : 0.035753
$Q_0 \rightarrow Q_{44}$	5.559	223.0	0.00429	114a→126a : 0.022125<br>117a→125a : 0.048765<br>123a→136a : 0.020161<br>124a→125a : 0.054021<br>124a→128a : 0.023731<br>124a→131a : 0.032717<br>111b→123b : 0.031439

				114b→123b : 0.020178<br>119b→128b : 0.032980<br>120b→128b : 0.101054<br>121b→128b : 0.122192
$Q_0 \rightarrow Q_{45}$	5.573	222.5	0.0038	114a→126a : 0.152073<br>117a→125a : 0.274878<br>117a→128a : 0.020733<br>118a→125a : 0.048158<br>122a→126a : 0.033947<br>123a→126a : 0.026181
$Q_0 \rightarrow Q_{46}$	5.583	222.1	0.23597	114a→125a : 0.094889<br>117a→126a : 0.108251<br>121a→126a : 0.049482<br>122a→125a : 0.028640<br>123a→125a : 0.022766<br>123a→128a : 0.080863<br>124a→127a : 0.067124<br>110b→125b : 0.020569<br>118b→122b : 0.023544<br>119b→123b : 0.025246<br>120b→127b : 0.051150<br>121b→126b : 0.043359
$Q_0 \rightarrow Q_{47}$	5.714	217.0	0.01084	120a→126a : 0.025662<br>123a→126a : 0.065529<br>124a→125a : 0.093793<br>124a→128a : 0.027137<br>124a→131a : 0.033404<br>119b→128b : 0.048771<br>120b→128b : 0.121771<br>121b→128b : 0.150785
$Q_0 \rightarrow Q_{48}$	5.748	215.7	0.00409	110b→122b : 0.236664<br>112b→123b : 0.062409<br>112b→124b : 0.052062<br>112b→125b : 0.042750<br>120b→122b : 0.054214<br>120b→123b : 0.022089<br>120b→128b : 0.038670<br>121b→122b : 0.055952<br>121b→123b : 0.024138<br>121b→128b : 0.047538
$Q_0 \rightarrow Q_{49}$	5.771	214.8	0.06788	120a→125a : 0.041881<br>123a→125a : 0.179725<br>124a→126a : 0.143280<br>111b→122b : 0.043380<br>114b→122b : 0.028478<br>119b→123b : 0.047824<br>119b→124b : 0.025701<br>120b→123b : 0.025775<br>121b→123b : 0.030514
$Q_0 \rightarrow Q_{50}$	5.787	214.2	0.00108	117a→125a : 0.020191

				120a→126a : 0.043978<br>120a→127a : 0.022967<br>120a→133a : 0.023333<br>121a→131a : 0.037175<br>123a→126a : 0.029793<br>124a→125a : 0.078085<br>113b→126b : 0.022823<br>118b→125b : 0.040396<br>118b→127b : 0.048072<br>118b→132b : 0.027015<br>119b→126b : 0.087344<br>119b→131b : 0.041836

Table S4. Selected spin-allowed electronic transitions for **Co-Me**, determined using TD-DFT calculations on its quartet (S = 3/2) ground state. It includes the excitation energies (in eV), corresponding wavelengths (λ), oscillator strengths (f), and major orbital contributions for these transitions. Given the open-shell nature of the quartet state, orbitals are categorized as either alpha (α) spin orbitals or beta (β) spin orbitals. The three singly occupied α-spin orbitals (114a to 116a) are responsible for forming the quartet configuration. All listed transitions are spin-conserving, meaning they involve either an α→α or a β→β excitation. The orbital indices refer to the unrestricted Kohn-Sham orbitals of the quartet reference state, and the numerical value accompanying each orbital excitation indicates the weight of that configuration in the overall transition character.

Transition	Energy (eV)	λ (nm)	f	Major contributions
$Q_0 \rightarrow Q_5$	1.855	668.5	0.00148	82b→ 94b : 0.154439<br>82b→ 96b : 0.024202<br>82b→ 98b : 0.034857<br>85b→ 94b : 0.396267<br>85b→ 96b : 0.060846<br>85b→ 98b : 0.084486<br>87b→ 94b : 0.087152
$Q_0 \rightarrow Q_6$	2.693	460.4	0.00849	91a→ 93a : 0.178769<br>92a→ 94a : 0.165084<br>84b→ 94b : 0.034916<br>88b→ 91b : 0.148304<br>89b→ 90b : 0.233939
$Q_0 \rightarrow Q_7$	2.698	459.6	0.00042	91a→ 93a : 0.024960<br>92a→ 94a : 0.026412<br>84b→ 94b : 0.510840<br>84b→ 96b : 0.082193<br>84b→ 98b : 0.097746<br>87b→ 94b : 0.025558

				89b→ 94b : 0.054649
$Q_0 \rightarrow Q_8$	2.704	458.5	0.00003	91a→ 94a : 0.191941
				92a→ 93a : 0.222420
				86b→ 90b : 0.020196
				88b→ 90b : 0.207390
				89b→ 91b : 0.176620
$Q_0 \rightarrow Q_9$	3.259	380.5	0.00017	88a→ 94a : 0.070123
				89a→ 93a : 0.127551
				90a→ 94a : 0.052501
				91a→ 96a : 0.057634
				92a→ 95a : 0.032230
				92a→ 96a : 0.026160
				86b→ 91b : 0.104188
				87b→ 90b : 0.113553
				87b→ 93b : 0.021242
				88b→ 91b : 0.028581
				88b→ 95b : 0.051685
				89b→ 96b : 0.042412
$Q_0 \rightarrow Q_{10}$	3.264	379.9	0.00059	88a→ 93a : 0.070897
				89a→ 94a : 0.122358
				90a→ 93a : 0.059317
				91a→ 95a : 0.061701
				92a→ 95a : 0.022666
				92a→ 96a : 0.034397
				86b→ 90b : 0.100371
				87b→ 91b : 0.116546
				88b→ 90b : 0.027683
				88b→ 96b : 0.042520
				89b→ 95b : 0.052502
$Q_0 \rightarrow Q_{11}$	3.658	338.8	0.04758	91a→ 93a : 0.204109
				92a→ 94a : 0.250720
				88b→ 91b : 0.092302
				89b→ 90b : 0.338167
$Q_0 \rightarrow Q_{12}$	3.745	331.1	0.13767	87a→ 94a : 0.027567
				90a→ 93a : 0.058393
				91a→ 94a : 0.094070
				92a→ 93a : 0.325150
				88b→ 90b : 0.281409
				89b→ 91b : 0.109186
$Q_0 \rightarrow Q_{13}$	4.095	302.7	0.04677	87a→ 94a : 0.092273
				88a→ 93a : 0.062541
				90a→ 93a : 0.410546
				91a→ 94a : 0.147565
				88b→ 90b : 0.076096
				88b→ 93b : 0.020538
$Q_0 \rightarrow Q_{14}$	4.150	298.8	0.00322	87a→ 93a : 0.146082
				88a→ 94a : 0.036981
				90a→ 94a : 0.353798
				90a→ 95a : 0.026781
				91a→ 93a : 0.064765

				91a→ 96a : 0.028375<br>92a→ 94a : 0.045019<br>87b→ 90b : 0.021194<br>88b→ 95b : 0.032400
$Q_0 \rightarrow Q_{15}$	4.232	292.9	0.00260	87a→ 93a : 0.044214<br>88a→ 94a : 0.091913<br>89a→ 93a : 0.051583<br>90a→ 94a : 0.041307<br>91a→ 93a : 0.020641<br>91a→ 95a : 0.020929<br>91a→ 96a : 0.076279<br>91a→ 98a : 0.020299<br>92a→ 94a : 0.021655<br>92a→ 95a : 0.103317<br>86b→ 91b : 0.047358<br>87b→ 90b : 0.067042<br>88b→ 92b : 0.042015<br>88b→ 95b : 0.081247<br>89b→ 94b : 0.058133<br>89b→ 96b : 0.045118
$Q_0 \rightarrow Q_{16}$	4.234	292.8	0.00061	88a→ 93a : 0.080976<br>89a→ 94a : 0.056430<br>91a→ 95a : 0.105971<br>91a→ 96a : 0.020863<br>92a→ 96a : 0.114182<br>86b→ 90b : 0.062909<br>87b→ 91b : 0.066835<br>88b→ 94b : 0.069380<br>88b→ 96b : 0.055879<br>88b→ 98b : 0.022064<br>89b→ 92b : 0.044143<br>89b→ 95b : 0.100681
$Q_0 \rightarrow Q_{17}$	4.528	273.8	0.03542	83b→ 90b : 0.059867<br>85b→ 91b : 0.020771<br>88b→ 90b : 0.090049<br>88b→ 93b : 0.418479<br>89b→ 91b : 0.196244
$Q_0 \rightarrow Q_{18}$	4.544	272.8	0.02717	91a→ 93a : 0.030304<br>85b→ 93b : 0.022990<br>87b→ 93b : 0.024184<br>88b→ 91b : 0.130783<br>88b→ 92b : 0.031244<br>89b→ 90b : 0.056635<br>89b→ 93b : 0.496868
$Q_0 \rightarrow Q_{19}$	4.668	265.6	0.00452	79b→ 92b : 0.034073<br>82b→ 90b : 0.024822<br>83b→ 91b : 0.027801<br>85b→ 90b : 0.048084<br>86b→ 92b : 0.032628<br>87b→ 90b : 0.077587

				88b→ 91b : 0.046336<br>88b→ 92b : 0.525956
$Q_0 \rightarrow Q_{20}$	4.684	264.7	0.00014	78b→ 92b : 0.026672<br>83b→ 90b : 0.021301<br>85b→ 92b : 0.028434<br>87b→ 92b : 0.038893<br>89b→ 92b : 0.655778
$Q_0 \rightarrow Q_{21}$	4.775	259.6	0.06313	82b→ 90b : 0.085784<br>82b→ 93b : 0.033792<br>83b→ 91b : 0.092350<br>85b→ 90b : 0.255218<br>87b→ 90b : 0.074034<br>88b→ 92b : 0.196021
$Q_0 \rightarrow Q_{22}$	4.818	257.3	0.00451	89a→ 94a : 0.020836<br>90a→ 93a : 0.027571<br>82b→ 91b : 0.028478<br>83b→ 90b : 0.358255<br>83b→ 93b : 0.026889<br>85b→ 91b : 0.157428<br>87b→ 91b : 0.056451<br>88b→ 93b : 0.074164<br>89b→ 92b : 0.036754<br>89b→ 95b : 0.021485
$Q_0 \rightarrow Q_{23}$	5.076	244.2	0.37621	88a→ 94a : 0.102360<br>89a→ 93a : 0.198023<br>90a→ 94a : 0.020935<br>91a→ 96a : 0.023423<br>85b→ 90b : 0.106922<br>86b→ 91b : 0.132791<br>87b→ 90b : 0.132176<br>88b→ 95b : 0.022691<br>89b→ 90b : 0.020385<br>89b→ 94b : 0.022240
$Q_0 \rightarrow Q_{24}$	5.161	240.2	0.04392	88a→ 93a : 0.130075<br>89a→ 94a : 0.140174<br>91a→ 95a : 0.041308<br>92a→ 96a : 0.034165<br>83b→ 90b : 0.050301<br>85b→ 91b : 0.022541<br>86b→ 90b : 0.174625<br>87b→ 91b : 0.102855<br>88b→ 94b : 0.051553<br>89b→ 95b : 0.027763
$Q_0 \rightarrow Q_{25}$	5.178	239.4	0.0039	83a→ 94a : 0.045147<br>84a→ 93a : 0.081542<br>88a→100a : 0.024203<br>89a→ 96a : 0.022828<br>89a→ 98a : 0.040579<br>75b→ 91b : 0.028469<br>79b→ 91b : 0.020349

				80b→ 90b : 0.055943<br>81b→ 90b : 0.020721<br>82b→ 90b : 0.036283<br>84b→ 90b : 0.034263<br>84b→ 93b : 0.027914<br>86b→100b : 0.035665<br>87b→ 96b : 0.028163<br>87b→ 98b : 0.033416
$Q_0 \rightarrow Q_{26}$	5.211	237.9	0.00474	77a→ 93a : 0.021289<br>83a→ 93a : 0.053660<br>84a→ 94a : 0.068966<br>88a→ 98a : 0.029650<br>89a→100a : 0.037588<br>75b→ 90b : 0.039454<br>80b→ 91b : 0.050368<br>83b→ 90b : 0.065852<br>83b→ 93b : 0.032325<br>84b→ 91b : 0.021063<br>86b→ 96b : 0.030305<br>86b→ 98b : 0.023370<br>87b→ 95b : 0.022747<br>87b→100b : 0.041724
$Q_0 \rightarrow Q_{27}$	5.382	230.3	0.00061	82b→ 90b : 0.133749<br>82b→ 93b : 0.036484<br>84b→ 90b : 0.246168<br>84b→ 93b : 0.039511<br>85b→ 90b : 0.044347<br>85b→ 93b : 0.092146<br>87b→ 93b : 0.020139<br>88b→ 91b : 0.043730<br>89b→ 90b : 0.029233<br>89b→ 93b : 0.053440<br>89b→ 94b : 0.030005<br>89b→ 96b : 0.051252
$Q_0 \rightarrow Q_{28}$	5.415	229.0	0.00875	87a→ 94a : 0.021834<br>83b→ 90b : 0.053871<br>83b→ 93b : 0.252030<br>85b→ 91b : 0.324630<br>86b→ 90b : 0.028785<br>87b→ 91b : 0.026922<br>88b→ 93b : 0.041264<br>89b→ 91b : 0.028466
$Q_0 \rightarrow Q_{29}$	5.497	225.6	0.00439	86a→ 93a : 0.092165<br>87a→ 94a : 0.149077<br>91a→ 94a : 0.027327<br>91a→100a : 0.023905<br>92a→ 93a : 0.058216<br>92a→ 98a : 0.065967<br>84b→ 91b : 0.172745<br>88b→ 94b : 0.075061

				88b→ 96b : 0.025510<br>89b→ 91b : 0.038459<br>89b→100b : 0.038344
$Q_0 \rightarrow Q_{30}$	5.536	224.0	0.03145	86a→ 94a : 0.086207<br>87a→ 93a : 0.162699<br>92a→ 94a : 0.030887<br>92a→100a : 0.029174<br>82b→ 90b : 0.109662<br>82b→ 93b : 0.059667<br>84b→ 90b : 0.043583<br>84b→ 93b : 0.026942<br>85b→ 93b : 0.077827<br>87b→ 93b : 0.028692<br>89b→ 94b : 0.044559<br>89b→ 96b : 0.020990
$Q_0 \rightarrow Q_{31}$	5.546	223.6	0.00049	86a→ 94a : 0.082823<br>87a→ 93a : 0.189541<br>91a→ 93a : 0.045870<br>92a→ 94a : 0.046493<br>82b→ 90b : 0.066913<br>83b→ 92b : 0.156048<br>85b→ 90b : 0.026963<br>85b→ 93b : 0.042386<br>89b→ 94b : 0.069091<br>89b→ 96b : 0.032832
$Q_0 \rightarrow Q_{32}$	5.583	222.1	0.00158	86a→ 93a : 0.137134<br>87a→ 94a : 0.206506<br>81b→ 90b : 0.034254<br>83b→ 93b : 0.025993<br>84b→ 91b : 0.113118<br>88b→ 94b : 0.103611<br>88b→ 96b : 0.039856<br>89b→100b : 0.021023
$Q_0 \rightarrow Q_{33}$	5.606	221.2	0.10397	86a→ 94a : 0.034051<br>87a→ 93a : 0.086756<br>92a→ 94a : 0.026288<br>74b→ 92b : 0.025426<br>83b→ 91b : 0.028888<br>83b→ 92b : 0.558932<br>84b→ 90b : 0.022540
$Q_0 \rightarrow Q_{34}$	5.668	218.8	0.52834	91a→ 96a : 0.038287<br>92a→ 95a : 0.041644<br>83b→ 92b : 0.046764<br>84b→ 90b : 0.062975<br>84b→ 93b : 0.029012<br>85b→ 94b : 0.021493<br>87b→ 90b : 0.027765<br>87b→ 94b : 0.024523<br>88b→ 95b : 0.021384<br>89b→ 94b : 0.382954

$Q_0 \rightarrow Q_{35}$	5.705	217.3	0.00582	81b→ 90b : 0.034511<br>82b→ 92b : 0.164511<br>83b→ 93b : 0.036597<br>84b→ 91b : 0.110359<br>85b→ 92b : 0.045485<br>86b→ 94b : 0.026590<br>88b→ 94b : 0.244537<br>88b→ 96b : 0.044746
$Q_0 \rightarrow Q_{36}$	5.833	212.5	0.06178	88a→ 94a : 0.040019<br>90a→ 94a : 0.040567<br>91a→ 93a : 0.191920<br>91a→ 98a : 0.102301<br>92a→ 94a : 0.112478<br>92a→100a : 0.055596<br>82b→ 93b : 0.029179<br>84b→ 90b : 0.030260<br>89b→ 94b : 0.027999
$Q_0 \rightarrow Q_{37}$	5.850	212.0	0.00516	86a→ 93a : 0.027277<br>87a→ 94a : 0.027339<br>88a→ 93a : 0.022223<br>90a→ 93a : 0.066652<br>91a→ 94a : 0.118431<br>92a→ 93a : 0.087560<br>92a→ 98a : 0.022492<br>82b→ 92b : 0.106695<br>84b→ 91b : 0.045145<br>85b→ 92b : 0.026514<br>87b→ 92b : 0.028067<br>88b→ 90b : 0.032736<br>88b→ 93b : 0.034392<br>89b→ 91b : 0.034904
$Q_0 \rightarrow Q_{38}$	5.903	210.0	0.01656	88a→ 93a : 0.036006<br>91a→ 94a : 0.141146<br>92a→ 93a : 0.117224<br>80b→ 90b : 0.032311<br>81b→ 90b : 0.094482<br>82b→ 92b : 0.197357<br>83b→ 93b : 0.022091<br>85b→ 92b : 0.045430<br>88b→ 94b : 0.039561
$Q_0 \rightarrow Q_{39}$	5.913	209.7	0.00889	88a→ 95a : 0.099480<br>89a→ 95a : 0.029925<br>89a→ 96a : 0.107651<br>90a→ 95a : 0.027772<br>82b→ 93b : 0.043830<br>83b→ 91b : 0.037930<br>86b→ 92b : 0.037670<br>86b→ 95b : 0.148164<br>87b→ 94b : 0.078065<br>87b→ 96b : 0.079189

$Q_0 \rightarrow Q_{40}$	5.929	209.1	0.00314	88a→ 96a : 0.053320<br>89a→ 95a : 0.029180<br>89a→ 96a : 0.044478<br>82b→ 90b : 0.039620<br>82b→ 93b : 0.100259<br>83b→ 91b : 0.071925<br>83b→ 92b : 0.030580<br>85b→ 90b : 0.039650<br>86b→ 94b : 0.033268<br>86b→ 96b : 0.032095<br>87b→ 95b : 0.045895<br>88b→ 91b : 0.048290<br>89b→ 90b : 0.020263<br>89b→ 93b : 0.021691
$Q_0 \rightarrow Q_{41}$	5.932	209.0	0.00204	88a→ 95a : 0.024454<br>88a→ 96a : 0.042411<br>89a→ 95a : 0.089984<br>82b→ 90b : 0.028660<br>82b→ 92b : 0.034976<br>82b→ 93b : 0.078653<br>83b→ 91b : 0.054308<br>83b→ 92b : 0.022117<br>85b→ 90b : 0.028013<br>85b→ 92b : 0.028227<br>86b→ 94b : 0.052981<br>86b→ 96b : 0.046687<br>87b→ 95b : 0.076583<br>88b→ 91b : 0.031658
$Q_0 \rightarrow Q_{42}$	5.966	207.8	0.0019	88a→ 93a : 0.021137<br>90a→ 93a : 0.042320<br>91a→ 94a : 0.084475<br>92a→ 93a : 0.045736<br>80b→ 90b : 0.033454<br>81b→ 90b : 0.102708<br>82b→ 91b : 0.026911<br>85b→ 91b : 0.050404<br>86b→ 93b : 0.021717<br>88b→ 90b : 0.095870<br>88b→ 93b : 0.086803<br>88b→ 96b : 0.031380<br>89b→ 91b : 0.110037
$Q_0 \rightarrow Q_{43}$	5.997	206.8	0.19855	88a→ 94a : 0.028832<br>91a→ 93a : 0.064837<br>91a→ 96a : 0.057143<br>92a→ 94a : 0.048865<br>92a→ 95a : 0.041628<br>83b→ 92b : 0.025832<br>84b→ 90b : 0.028939<br>85b→ 90b : 0.030763<br>87b→ 90b : 0.035726

				88b→ 91b : 0.083964<br>88b→ 95b : 0.129127<br>88b→100b : 0.035582<br>89b→ 93b : 0.045005<br>89b→ 96b : 0.130286
$Q_0 \rightarrow Q_{44}$	6.047	205.0	0.05189	91a→100a : 0.094927<br>92a→ 98a : 0.105390<br>80b→ 90b : 0.021140<br>81b→ 90b : 0.065181<br>82b→ 92b : 0.035400<br>83b→ 94b : 0.040947<br>86b→ 90b : 0.032279<br>88b→ 94b : 0.143652<br>88b→ 98b : 0.078769<br>89b→100b : 0.043761
$Q_0 \rightarrow Q_{45}$	6.083	203.8	0.00742	80b→ 90b : 0.029634<br>81b→ 90b : 0.085267<br>81b→ 93b : 0.029901<br>82b→ 91b : 0.295695<br>83b→ 90b : 0.058611<br>83b→ 93b : 0.206009<br>85b→ 91b : 0.063256
$Q_0 \rightarrow Q_{46}$	6.110	202.9	0.09504	88a→ 93a : 0.028767<br>91a→ 94a : 0.023321<br>91a→ 95a : 0.110180<br>91a→100a : 0.021827<br>92a→ 96a : 0.108934<br>86b→ 90b : 0.106503<br>88b→ 96b : 0.153078<br>89b→ 95b : 0.170427
$Q_0 \rightarrow Q_{47}$	6.124	202.5	0.00117	91a→ 98a : 0.024494<br>92a→ 94a : 0.025167<br>81b→ 91b : 0.022919<br>82b→ 90b : 0.020576<br>82b→ 93b : 0.062821<br>83b→ 91b : 0.095642<br>84b→ 90b : 0.058458<br>85b→ 93b : 0.028013<br>88b→ 91b : 0.097490<br>89b→ 90b : 0.077558<br>89b→ 93b : 0.038941<br>89b→ 96b : 0.064886
$Q_0 \rightarrow Q_{48}$	6.137	202.0	0.01745	90a→ 94a : 0.061935<br>91a→ 93a : 0.026878<br>91a→ 98a : 0.072914<br>92a→ 94a : 0.061351<br>92a→ 95a : 0.031019<br>92a→100a : 0.062204<br>84b→ 90b : 0.047312<br>88b→ 91b : 0.058780

				88b→100b : 0.022503<br>89b→ 90b : 0.033449<br>89b→ 93b : 0.036054<br>89b→ 94b : 0.027213<br>89b→ 98b : 0.083150

Table S5. Selected spin-allowed electronic transitions for **Co-EtO**, determined using TD-DFT calculations on its quartet (S = 3/2) ground state. It includes the excitation energies (in eV), corresponding wavelengths (λ), oscillator strengths (f), and major orbital contributions for these transitions. Given the open-shell nature of the quartet state, orbitals are categorized as either alpha (α) spin orbitals or beta (β) spin orbitals. The three singly occupied α-spin orbitals (114a to 116a) are responsible for forming the quartet configuration. All listed transitions are spin-conserving, meaning they involve either an α→α or a β→β excitation. The orbital indices refer to the unrestricted Kohn-Sham orbitals of the quartet reference state, and the numerical value accompanying each orbital excitation indicates the weight of that configuration in the overall transition character.

Transition	Energy (eV)	λ (nm)	f	Major contributions
$Q_0 \rightarrow Q_5$	1.887	657.2	0.00130	97b→110b : 0.126422<br>97b→114b : 0.020167<br>101b→109b : 0.041232<br>101b→110b : 0.394478<br>101b→112b : 0.051243<br>101b→114b : 0.059782<br>101b→115b : 0.031763<br>103b→110b : 0.068696
$Q_0 \rightarrow Q_6$	2.504	495.1	0.00009	108a→109a : 0.177018<br>108a→110a : 0.241744<br>105b→106b : 0.215658<br>105b→107b : 0.185139
$Q_0 \rightarrow Q_7$	2.651	467.8	0.00007	107a→109a : 0.266455<br>107a→110a : 0.153509<br>108a→109a : 0.023307<br>104b→106b : 0.227559<br>104b→107b : 0.155020
$Q_0 \rightarrow Q_8$	2.723	455.4	0.00482	99b→109b : 0.030996<br>99b→110b : 0.524031<br>99b→112b : 0.067778<br>99b→114b : 0.072094<br>99b→115b : 0.038445<br>105b→110b : 0.044946
$Q_0 \rightarrow Q_9$	3.165	391.7	0.00045	105a→109a : 0.045652<br>105a→110a : 0.050724

				106a→109a : 0.059911<br>106a→110a : 0.090376<br>108a→112a : 0.126782<br>102b→106b : 0.020253<br>102b→107b : 0.034079<br>103b→106b : 0.088524<br>103b→107b : 0.080364<br>105b→111b : 0.021810<br>105b→112b : 0.094196
$Q_0 \rightarrow Q_{10}$	3.202	387.2	0.00059	104a→109a : 0.053680<br>104a→110a : 0.031306<br>105a→109a : 0.087863<br>105a→110a : 0.063834<br>106a→109a : 0.032837<br>107a→111a : 0.109051<br>102b→106b : 0.088066<br>102b→107b : 0.070211<br>103b→106b : 0.033323<br>103b→107b : 0.041429<br>104b→106b : 0.024261<br>104b→111b : 0.084918
$Q_0 \rightarrow Q_{11}$	3.555	348.8	0.03635	107a→109a : 0.066383<br>108a→109a : 0.108966<br>108a→110a : 0.248139<br>103b→106b : 0.024520<br>104b→107b : 0.033112<br>105b→106b : 0.305432<br>105b→107b : 0.072373
$Q_0 \rightarrow Q_{12}$	3.660	338.7	0.12545	103a→110a : 0.021243<br>106a→109a : 0.043746<br>107a→109a : 0.195517<br>107a→110a : 0.078614<br>108a→109a : 0.135624<br>102b→106b : 0.022602<br>104b→106b : 0.281313<br>104b→107b : 0.059180<br>105b→107b : 0.047182
$Q_0 \rightarrow Q_{13}$	4.000	310.0	0.02623	103a→110a : 0.060015<br>104a→109a : 0.049093<br>106a→109a : 0.227264<br>106a→110a : 0.043934<br>107a→110a : 0.095238<br>108a→112a : 0.082866<br>104b→106b : 0.033402<br>105b→108b : 0.023292<br>105b→109b : 0.020804<br>105b→110b : 0.020925<br>105b→111b : 0.023513<br>105b→112b : 0.052695
$Q_0 \rightarrow Q_{14}$	4.062	305.3	0.01594	103a→109a : 0.073423

				104a→109a : 0.038000<br>105a→109a : 0.053237<br>106a→109a : 0.092725<br>106a→110a : 0.166527<br>107a→109a : 0.020188<br>107a→111a : 0.022215<br>108a→110a : 0.033399<br>108a→112a : 0.053187<br>103b→106b : 0.029987<br>104b→106b : 0.034428<br>104b→111b : 0.040721<br>105b→110b : 0.020625<br>105b→112b : 0.032066
$Q_0 \rightarrow Q_{15}$	4.139	299.6	0.00507	103a→109a : 0.081462<br>103a→110a : 0.027912<br>104a→109a : 0.048354<br>104a→110a : 0.109833<br>105a→109a : 0.021814<br>105a→110a : 0.085044<br>106a→110a : 0.074146<br>107a→109a : 0.040158<br>107a→110a : 0.050460<br>108a→112a : 0.102971<br>103b→107b : 0.035739<br>105b→108b : 0.021896<br>105b→110b : 0.024881<br>105b→111b : 0.020787<br>105b→112b : 0.033386
$Q_0 \rightarrow Q_{16}$	4.197	295.4	0.00343	103a→109a : 0.029747<br>104a→109a : 0.064206<br>104a→110a : 0.048678<br>106a→110a : 0.023259<br>107a→111a : 0.213800<br>107a→114a : 0.021725<br>102b→106b : 0.047742<br>102b→107b : 0.032671<br>103b→106b : 0.022975<br>103b→107b : 0.021111<br>104b→108b : 0.027723<br>104b→110b : 0.061923<br>104b→111b : 0.118099<br>104b→114b : 0.029866
$Q_0 \rightarrow Q_{17}$	4.420	280.5	0.03852	98b→106b : 0.027580<br>103b→109b : 0.033458<br>104b→106b : 0.021601<br>104b→109b : 0.077364<br>105b→106b : 0.064959<br>105b→107b : 0.159616<br>105b→109b : 0.359627
$Q_0 \rightarrow Q_{18}$	4.493	276.0	0.03698	107a→109a : 0.025861

				104b→106b : 0.061332<br>104b→107b : 0.159738<br>104b→109b : 0.359872<br>105b→109b : 0.104872
$Q_0 \rightarrow Q_{19}$	4.552	272.4	0.00336	108a→112a : 0.023817<br>91b→108b : 0.033279<br>102b→108b : 0.031843<br>103b→106b : 0.043019<br>103b→108b : 0.049617<br>104b→107b : 0.022138<br>104b→108b : 0.104238<br>105b→108b : 0.483423
$Q_0 \rightarrow Q_{20}$	4.629	267.9	0.00225	92b→108b : 0.025172<br>101b→106b : 0.042243<br>102b→106b : 0.024791<br>103b→106b : 0.036033<br>103b→108b : 0.023282<br>104b→108b : 0.373547<br>105b→108b : 0.156594
$Q_0 \rightarrow Q_{21}$	4.713	263.1	0.09861	105a→109a : 0.029165<br>106a→110a : 0.026410<br>97b→106b : 0.059553<br>97b→109b : 0.022489<br>98b→107b : 0.072273<br>101b→106b : 0.193533<br>101b→109b : 0.020743<br>102b→108b : 0.023818<br>103b→106b : 0.089568<br>104b→108b : 0.235181
$Q_0 \rightarrow Q_{22}$	4.764	260.3	0.00016	105a→110a : 0.032540<br>106a→109a : 0.034087<br>97b→107b : 0.025853<br>98b→106b : 0.262791<br>100b→106b : 0.043072<br>101b→107b : 0.148728<br>102b→106b : 0.021672<br>103b→107b : 0.072111<br>104b→108b : 0.026902<br>104b→109b : 0.058379<br>105b→108b : 0.025067
$Q_0 \rightarrow Q_{23}$	4.983	248.8	0.23123	104a→110a : 0.058135<br>105a→109a : 0.128405<br>105a→110a : 0.027648<br>106a→109a : 0.021053<br>106a→110a : 0.042112<br>108a→112a : 0.030916<br>101b→106b : 0.133032<br>102b→107b : 0.104335<br>103b→106b : 0.105204<br>103b→107b : 0.042648

				105b→106b : 0.029552
$Q_0 \rightarrow Q_{24}$	5.095	243.3	0.08832	104a→109a : 0.111962<br>105a→109a : 0.030623<br>105a→110a : 0.092948<br>107a→111a : 0.066859<br>98b→106b : 0.054216<br>101b→106b : 0.029212<br>101b→107b : 0.045473<br>102b→106b : 0.154160<br>102b→107b : 0.030125<br>103b→107b : 0.052586<br>104b→110b : 0.036970
$Q_0 \rightarrow Q_{25}$	5.154	240.6	0.00726	100a→109a : 0.022790<br>105a→114a : 0.036410<br>89b→107b : 0.022027<br>90b→106b : 0.032150<br>92b→106b : 0.025685<br>97b→106b : 0.023930<br>99b→106b : 0.070775<br>99b→109b : 0.032480<br>100b→106b : 0.030367<br>102b→114b : 0.028677
$Q_0 \rightarrow Q_{26}$	5.217	237.7	0.00149	100a→110a : 0.033489<br>105a→116a : 0.030091<br>89b→106b : 0.036310<br>96b→107b : 0.025473<br>98b→106b : 0.056937<br>98b→109b : 0.032377<br>99b→106b : 0.062521<br>99b→107b : 0.034998<br>103b→116b : 0.037420
$Q_0 \rightarrow Q_{27}$	5.316	233.2	0.00420	97b→109b : 0.028467<br>99b→106b : 0.183372<br>99b→109b : 0.023258<br>101b→106b : 0.025662<br>101b→109b : 0.060293<br>105b→106b : 0.021736<br>105b→107b : 0.029377<br>105b→109b : 0.037977<br>105b→110b : 0.030989<br>105b→112b : 0.028625
$Q_0 \rightarrow Q_{28}$	5.352	231.7	0.00713	101a→109a : 0.032790<br>103a→110a : 0.053145<br>98b→106b : 0.052944<br>98b→109b : 0.204753<br>99b→106b : 0.021053<br>101b→107b : 0.228610<br>103b→107b : 0.027286<br>104b→109b : 0.031408
$Q_0 \rightarrow Q_{29}$	5.382	230.4	0.00569	101a→109a : 0.071020

				103a→109a : 0.036031<br>103a→110a : 0.122331<br>108a→109a : 0.081377<br>108a→110a : 0.026682<br>108a→114a : 0.038269<br>99b→107b : 0.126695<br>101b→107b : 0.045981<br>104b→110b : 0.037813<br>105b→107b : 0.039678<br>105b→110b : 0.044262<br>105b→116b : 0.021028
$Q_0 \rightarrow Q_{30}$	5.449	227.5	0.01033	101a→110a : 0.157361<br>103a→109a : 0.337578<br>103a→110a : 0.038135<br>107a→109a : 0.036915<br>107a→110a : 0.033221<br>108a→110a : 0.051980
$Q_0 \rightarrow Q_{31}$	5.474	226.5	0.01968	97b→106b : 0.154374<br>97b→109b : 0.040545<br>98b→108b : 0.035668<br>101b→106b : 0.049368<br>101b→109b : 0.094732<br>103b→109b : 0.036121<br>105b→110b : 0.125957<br>105b→112b : 0.032487
$Q_0 \rightarrow Q_{32}$	5.500	225.4	0.02555	101a→109a : 0.072272<br>103a→110a : 0.135130<br>108a→109a : 0.022799<br>93b→106b : 0.021096<br>98b→109b : 0.025020<br>99b→107b : 0.095988<br>101b→107b : 0.032131<br>104b→110b : 0.045640<br>104b→111b : 0.024337<br>105b→110b : 0.092644
$Q_0 \rightarrow Q_{33}$	5.565	222.8	0.02011	97b→109b : 0.036534<br>98b→107b : 0.030513<br>98b→108b : 0.419832<br>99b→106b : 0.030401<br>100b→108b : 0.049685<br>104b→110b : 0.054748
$Q_0 \rightarrow Q_{34}$	5.597	221.5	0.49635	107a→111a : 0.024306<br>108a→112a : 0.051815<br>98b→108b : 0.119607<br>99b→106b : 0.025559<br>99b→107b : 0.062419<br>103b→106b : 0.022125<br>104b→107b : 0.031620<br>104b→110b : 0.028833<br>105b→110b : 0.171924

$Q_0 \rightarrow Q_{35}$	5.646	219.6	0.07649	107a→111a : 0.023227<br>97b→108b : 0.067529<br>98b→108b : 0.041211<br>98b→109b : 0.045883<br>99b→107b : 0.107151<br>102b→110b : 0.026363<br>104b→110b : 0.228777<br>104b→112b : 0.038878<br>105b→106b : 0.023895<br>105b→107b : 0.039722
$Q_0 \rightarrow Q_{36}$	5.673	218.5	0.00966	101a→109a : 0.025199<br>104a→109a : 0.040277<br>104a→110a : 0.040545<br>107a→109a : 0.039416<br>107a→114a : 0.031982<br>108a→109a : 0.190122<br>108a→110a : 0.110752<br>108a→116a : 0.038986<br>99b→106b : 0.028392
$Q_0 \rightarrow Q_{37}$	5.728	216.4	0.00480	97b→109b : 0.099427<br>100b→106b : 0.132198<br>100b→107b : 0.145887<br>100b→109b : 0.034271<br>101b→106b : 0.063482<br>101b→107b : 0.045401<br>105b→106b : 0.033791<br>105b→107b : 0.046364<br>105b→109b : 0.025295
$Q_0 \rightarrow Q_{38}$	5.762	215.2	0.06640	107a→109a : 0.065394<br>107a→110a : 0.111157<br>107a→114a : 0.060032<br>93b→106b : 0.024087<br>97b→108b : 0.072406<br>104b→110b : 0.022378<br>104b→114b : 0.026779<br>105b→106b : 0.028085<br>105b→107b : 0.059047<br>105b→109b : 0.024100
$Q_0 \rightarrow Q_{39}$	5.822	213.0	0.00318	104a→109a : 0.039468<br>107a→109a : 0.036021<br>107a→110a : 0.150391<br>108a→109a : 0.058646<br>108a→110a : 0.022051<br>108a→114a : 0.022123<br>108a→116a : 0.036228<br>97b→108b : 0.087784<br>99b→106b : 0.021971<br>103b→108b : 0.021654<br>105b→106b : 0.022342<br>105b→112b : 0.053078

				105b→116b : 0.037465
$Q_0 \rightarrow Q_{40}$	5.852	211.9	0.03989	104a→112a : 0.052087<br>105a→112a : 0.145309<br>106a→112a : 0.132512<br>108a→112a : 0.053129<br>102b→111b : 0.029242<br>102b→112b : 0.057029<br>103b→108b : 0.021231<br>103b→110b : 0.067224<br>103b→111b : 0.040281<br>103b→112b : 0.145239
$Q_0 \rightarrow Q_{41}$	5.879	210.9	0.03499	93b→106b : 0.034787<br>97b→107b : 0.029775<br>97b→108b : 0.064772<br>97b→109b : 0.047899<br>98b→107b : 0.030274<br>98b→109b : 0.034648<br>101b→106b : 0.026636<br>101b→107b : 0.038918<br>102b→111b : 0.026207<br>104b→106b : 0.044747<br>104b→107b : 0.113338<br>104b→109b : 0.057221
$Q_0 \rightarrow Q_{42}$	5.896	210.3	0.00413	104a→111a : 0.064054<br>105a→111a : 0.088269<br>107a→109a : 0.026948<br>107a→110a : 0.032621<br>97b→108b : 0.082571<br>101b→108b : 0.024534<br>102b→110b : 0.046364<br>102b→111b : 0.100573<br>103b→111b : 0.046314<br>105b→112b : 0.023040
$Q_0 \rightarrow Q_{43}$	5.905	210.0	0.07109	104a→111a : 0.034700<br>105a→111a : 0.054069<br>107a→109a : 0.028816<br>107a→110a : 0.026742<br>107a→111a : 0.037821<br>93b→106b : 0.040971<br>98b→108b : 0.027315<br>100b→106b : 0.031056<br>100b→107b : 0.028586<br>102b→110b : 0.020274<br>102b→111b : 0.022261<br>103b→111b : 0.024400<br>104b→111b : 0.029001<br>105b→107b : 0.043200<br>105b→109b : 0.026161<br>105b→112b : 0.054389
$Q_0 \rightarrow Q_{44}$	5.936	208.9	0.03776	102a→109a : 0.054042

				102a→110a : 0.034239<br>108a→112a : 0.021999<br>93b→106b : 0.045122<br>97b→108b : 0.084216<br>99b→106b : 0.026733<br>100b→106b : 0.050830<br>100b→107b : 0.035090<br>101b→108b : 0.024747<br>103b→106b : 0.021931<br>104b→110b : 0.038222<br>105b→112b : 0.039938
$Q_0 \rightarrow Q_{45}$	5.979	207.4	0.02071	102a→109a : 0.283682<br>102a→110a : 0.156897<br>102a→114a : 0.033650<br>93b→106b : 0.065131<br>104b→111b : 0.034799
$Q_0 \rightarrow Q_{46}$	5.997	206.7	0.04709	102a→109a : 0.068165<br>102a→110a : 0.048699<br>107a→114a : 0.025578<br>107a→116a : 0.043908<br>108a→114a : 0.066452<br>108a→116a : 0.052699<br>97b→108b : 0.056222<br>98b→110b : 0.034758<br>104b→107b : 0.022299<br>104b→110b : 0.080859<br>104b→114b : 0.021455<br>105b→110b : 0.024987<br>105b→112b : 0.022145
$Q_0 \rightarrow Q_{47}$	6.032	205.5	0.17369	102a→109a : 0.028502<br>106a→110a : 0.028228<br>107a→111a : 0.056516<br>107a→114a : 0.030504<br>108a→112a : 0.113853<br>108a→116a : 0.049564<br>99b→106b : 0.024482<br>102b→106b : 0.050773<br>104b→111b : 0.057958<br>104b→112b : 0.030282<br>104b→114b : 0.021577<br>105b→111b : 0.044102<br>105b→112b : 0.073545
$Q_0 \rightarrow Q_{48}$	6.052	204.9	0.01660	93b→106b : 0.109987<br>97b→107b : 0.053319<br>97b→109b : 0.076636<br>98b→107b : 0.073089<br>98b→109b : 0.063261<br>99b→106b : 0.037069<br>104b→107b : 0.057034<br>104b→109b : 0.021339

				105b→106b : 0.028488<br>105b→112b : 0.025041
$Q_0 \rightarrow Q_{49}$	6.075	204.1	0.04297	107a→110a : 0.035786<br>107a→111a : 0.131723<br>107a→114a : 0.082850<br>107a→116a : 0.026974<br>108a→116a : 0.026586<br>102b→106b : 0.035076<br>104b→111b : 0.118617<br>104b→112b : 0.028336
$Q_0 \rightarrow Q_{50}$	6.152	201.5	0.00681	107a→114a : 0.021099<br>93b→106b : 0.045420<br>97b→106b : 0.046181<br>97b→107b : 0.075414<br>97b→109b : 0.050198<br>98b→106b : 0.068695<br>98b→107b : 0.119974<br>98b→109b : 0.044551<br>100b→106b : 0.080614<br>100b→107b : 0.039849<br>101b→107b : 0.026956

Table S6. Selected spin-allowed electronic transitions for **Co-Cl**, determined using TD-DFT calculations on its quartet (S = 3/2) ground state. It includes the excitation energies (in eV), corresponding wavelengths (λ), oscillator strengths (f), and major orbital contributions for these transitions. Given the open-shell nature of the quartet state, orbitals are categorized as either alpha (α) spin orbitals or beta (β) spin orbitals. The three singly occupied α-spin orbitals (114a to 116a) are responsible for forming the quartet configuration. All listed transitions are spin-conserving, meaning they involve either an α→α or a β→β excitation. The orbital indices refer to the unrestricted Kohn-Sham orbitals of the quartet reference state, and the numerical value accompanying each orbital excitation indicates the weight of that configuration in the overall transition character.

Transition	Energy (eV)	λ (nm)	f	Major contributions
$Q_0 \rightarrow Q_5$	2.157	574.8	0.00076	88b→102b : 0.134248<br>88b→105b : 0.027726<br>92b→101b : 0.089331<br>93b→102b : 0.332509<br>93b→103b : 0.042316<br>93b→105b : 0.064084<br>95b→102b : 0.035126<br>97b→102b : 0.069553
$Q_0 \rightarrow Q_6$	2.518	492.4	0.00389	92b→102b : 0.559936

				92b→103b : 0.073432<br>92b→104b : 0.024685<br>92b→105b : 0.109095<br>94b→102b : 0.072496
$Q_0 \rightarrow Q_7$	2.728	454.6	0.00003	99a→101a : 0.059149<br>99a→102a : 0.091392<br>100a→101a : 0.157127<br>100a→102a : 0.091794<br>94b→ 98b : 0.036690<br>96b→ 98b : 0.165323<br>97b→ 98b : 0.169302<br>97b→ 99b : 0.036406
$Q_0 \rightarrow Q_8$	2.763	448.7	0.00004	99a→101a : 0.151176<br>99a→102a : 0.097463<br>100a→101a : 0.069550<br>100a→102a : 0.113566<br>94b→ 99b : 0.024651<br>95b→ 99b : 0.023474<br>96b→ 99b : 0.165450<br>97b→ 98b : 0.027645<br>97b→ 99b : 0.173484
$Q_0 \rightarrow Q_9$	3.190	388.7	0.00011	96a→101a : 0.059547<br>97a→102a : 0.109192<br>98a→101a : 0.048238<br>99a→101a : 0.026891<br>99a→103a : 0.053569<br>100a→102a : 0.023380<br>100a→104a : 0.060068<br>94b→ 98b : 0.065729<br>94b→ 99b : 0.056222<br>95b→ 98b : 0.070361<br>95b→100b : 0.027346<br>96b→103b : 0.027642<br>96b→104b : 0.021362<br>97b→104b : 0.044368
$Q_0 \rightarrow Q_{10}$	3.192	388.4	0.00011	96a→102a : 0.058669<br>97a→101a : 0.115893<br>98a→102a : 0.045311<br>99a→102a : 0.027324<br>99a→104a : 0.058492<br>100a→101a : 0.021339<br>100a→103a : 0.055610<br>94b→ 98b : 0.048556<br>94b→ 99b : 0.026167<br>94b→100b : 0.021714<br>95b→ 99b : 0.142269<br>96b→103b : 0.024296<br>96b→104b : 0.027400<br>97b→103b : 0.046991
$Q_0 \rightarrow Q_{11}$	3.706	334.5	0.04510	99a→101a : 0.028236

				99a→102a : 0.153031<br>100a→101a : 0.198167<br>100a→102a : 0.034438<br>96b→ 98b : 0.245732<br>97b→ 98b : 0.143738<br>97b→ 99b : 0.074422
$Q_0 \to Q_{12}$	3.815	325.0	0.12781	98a→101a : 0.066574<br>99a→101a : 0.232751<br>99a→102a : 0.045954<br>100a→102a : 0.085823<br>96b→ 99b : 0.161141<br>97b→ 98b : 0.124185<br>97b→ 99b : 0.153890
$Q_0 \to Q_{13}$	4.100	302.4	0.03097	95a→102a : 0.109736<br>96a→101a : 0.156937<br>98a→101a : 0.402496<br>100a→102a : 0.095614<br>97b→ 98b : 0.053300
$Q_0 \to Q_{14}$	4.159	298.1	0.01065	97a→101a : 0.049875<br>98a→102a : 0.034241<br>99a→104a : 0.092291<br>100a→103a : 0.099102<br>100a→106a : 0.020244<br>94b→ 98b : 0.022589<br>95b→ 99b : 0.054869<br>96b→ 98b : 0.045087<br>96b→100b : 0.047423<br>96b→101b : 0.080507<br>96b→104b : 0.053408<br>97b→102b : 0.057422<br>97b→103b : 0.042293<br>97b→105b : 0.021291
$Q_0 \to Q_{15}$	4.178	296.7	0.00994	96a→101a : 0.036994<br>97a→102a : 0.055213<br>99a→103a : 0.108399<br>99a→106a : 0.021143<br>100a→104a : 0.121592<br>94b→ 98b : 0.029296<br>94b→ 99b : 0.039869<br>95b→ 98b : 0.027935<br>96b→102b : 0.065368<br>96b→103b : 0.045126<br>96b→105b : 0.020562<br>97b→100b : 0.028297<br>97b→101b : 0.078011<br>97b→104b : 0.065785
$Q_0 \to Q_{16}$	4.207	294.7	0.00106	95a→101a : 0.197637<br>96a→102a : 0.143886<br>98a→102a : 0.383686<br>98a→104a : 0.020468

				99a→102a : 0.043738<br>100a→101a : 0.049925
$Q_0 \rightarrow Q_{17}$	4.353	284.8	0.01780	99a→104a : 0.024420<br>83b→100b : 0.021813<br>94b→ 98b : 0.021553<br>96b→ 98b : 0.162324<br>96b→100b : 0.186401<br>97b→ 98b : 0.100274<br>97b→100b : 0.157410
$Q_0 \rightarrow Q_{18}$	4.460	278.0	0.02484	84b→100b : 0.023077<br>93b→ 98b : 0.020651<br>96b→ 99b : 0.077690<br>96b→100b : 0.230329<br>97b→ 99b : 0.048726<br>97b→100b : 0.317601
$Q_0 \rightarrow Q_{19}$	4.685	264.6	0.03303	99a→104a : 0.034856<br>100a→103a : 0.034254<br>83b→101b : 0.034266<br>94b→101b : 0.043481<br>96b→101b : 0.530860<br>97b→101b : 0.094879
$Q_0 \rightarrow Q_{20}$	4.716	262.9	0.00409	99a→103a : 0.021040<br>100a→104a : 0.029491<br>84b→101b : 0.038122<br>93b→101b : 0.047670<br>94b→ 98b : 0.023144<br>96b→101b : 0.112553<br>97b→101b : 0.480906
$Q_0 \rightarrow Q_{21}$	4.805	258.0	0.02994	98a→102a : 0.021260<br>88b→ 98b : 0.060930<br>90b→ 98b : 0.023773<br>91b→ 98b : 0.380712<br>91b→100b : 0.034766<br>93b→ 98b : 0.125660<br>93b→ 99b : 0.050029<br>95b→ 98b : 0.021736<br>96b→100b : 0.048729
$Q_0 \rightarrow Q_{22}$	4.914	252.3	0.03562	97a→101a : 0.022552<br>88b→ 98b : 0.102941<br>88b→ 99b : 0.027986<br>88b→100b : 0.031992<br>91b→ 99b : 0.119964<br>93b→ 98b : 0.075209<br>93b→ 99b : 0.186857<br>95b→ 99b : 0.057436<br>96b→ 99b : 0.039461<br>97b→103b : 0.026716
$Q_0 \rightarrow Q_{23}$	5.039	246.0	0.18439	96a→102a : 0.066341<br>97a→101a : 0.099431<br>91b→ 98b : 0.128710

				91b→ 99b : 0.026016<br>91b→100b : 0.075079<br>91b→101b : 0.025888<br>94b→ 98b : 0.061639<br>95b→ 98b : 0.025946<br>95b→ 99b : 0.049615<br>96b→ 98b : 0.026289<br>96b→104b : 0.024661<br>97b→104b : 0.022734
$Q_0 \rightarrow Q_{24}$	5.072	244.4	0.07383	80a→101a : 0.020665<br>90a→101a : 0.039310<br>97a→101a : 0.031579<br>97a→107a : 0.021142<br>82b→ 98b : 0.023353<br>87b→ 98b : 0.034152<br>92b→ 98b : 0.036236<br>92b→ 99b : 0.053871<br>94b→103b : 0.020720<br>94b→105b : 0.022677<br>95b→ 99b : 0.025914
$Q_0 \rightarrow Q_{25}$	5.081	244.0	0.06443	91a→101a : 0.054808<br>97a→101a : 0.022496<br>97a→103a : 0.021977<br>97a→106a : 0.022373<br>82b→ 99b : 0.027002<br>87b→ 99b : 0.030366<br>91b→ 98b : 0.020566<br>91b→ 99b : 0.021702<br>91b→100b : 0.020782<br>92b→ 99b : 0.051024<br>94b→ 98b : 0.032970<br>95b→103b : 0.028681<br>95b→105b : 0.024695
$Q_0 \rightarrow Q_{26}$	5.145	241.0	0.03236	96a→101a : 0.096208<br>97a→102a : 0.120377<br>98a→101a : 0.025643<br>99a→103a : 0.027167<br>100a→104a : 0.034006<br>88b→101b : 0.027527<br>94b→ 98b : 0.044700<br>94b→ 99b : 0.092319<br>95b→ 98b : 0.049299<br>95b→ 99b : 0.054623<br>95b→100b : 0.021550<br>96b→102b : 0.054622<br>97b→101b : 0.038139<br>97b→104b : 0.029723
$Q_0 \rightarrow Q_{27}$	5.246	236.3	0.08838	96a→102a : 0.023598<br>97a→101a : 0.025424<br>88b→100b : 0.020708

				91b→ 98b : 0.029183<br>91b→100b : 0.151672<br>93b→ 98b : 0.062961<br>93b→ 99b : 0.226579<br>94b→ 98b : 0.027426<br>94b→ 99b : 0.025082<br>96b→100b : 0.026290<br>97b→ 99b : 0.046098
$Q_0 \rightarrow Q_{28}$	5.424	228.6	0.00511	88b→ 98b : 0.141445<br>88b→100b : 0.149489<br>91b→ 99b : 0.063438<br>93b→ 98b : 0.025607<br>93b→100b : 0.213215<br>95b→100b : 0.038676<br>96b→ 99b : 0.043960<br>97b→ 98b : 0.033418<br>97b→100b : 0.046334
$Q_0 \rightarrow Q_{29}$	5.476	226.4	0.18751	91b→100b : 0.049771<br>91b→101b : 0.718540
$Q_0 \rightarrow Q_{30}$	5.507	225.1	0.02293	92a→101a : 0.066481<br>95a→102a : 0.115245<br>99a→101a : 0.042272<br>99a→106a : 0.069423<br>100a→102a : 0.021637<br>100a→107a : 0.027773<br>91b→101b : 0.022968<br>92b→ 99b : 0.155396<br>96b→102b : 0.044009<br>96b→103b : 0.030339<br>97b→107b : 0.028014
$Q_0 \rightarrow Q_{31}$	5.565	222.8	0.07730	92a→101a : 0.026622<br>92a→102a : 0.043834<br>95a→101a : 0.075334<br>95a→102a : 0.050129<br>99a→107a : 0.030635<br>100a→106a : 0.033122<br>88b→ 99b : 0.031078<br>91b→100b : 0.042373<br>92b→ 98b : 0.021218<br>96b→107b : 0.025649<br>97b→102b : 0.156729<br>97b→103b : 0.039667
$Q_0 \rightarrow Q_{32}$	5.607	221.1	0.03311	92a→101a : 0.024704<br>95a→101a : 0.027079<br>95a→102a : 0.056831<br>88b→101b : 0.125365<br>93b→101b : 0.050584<br>94b→102b : 0.025112<br>96b→102b : 0.257823<br>96b→103b : 0.060026

$Q_0 \rightarrow Q_{33}$	5.614	220.9	0.05375	90a→102a : 0.021449<br>92a→102a : 0.101838<br>95a→101a : 0.298147<br>95a→102a : 0.029316<br>95a→103a : 0.023775<br>98a→102a : 0.023729<br>99a→102a : 0.059808<br>100a→101a : 0.054939<br>97b→102b : 0.049297
$Q_0 \rightarrow Q_{34}$	5.671	218.6	0.13585	92a→101a : 0.044551<br>95a→102a : 0.101662<br>99a→101a : 0.025066<br>88b→101b : 0.082085<br>92b→ 98b : 0.035460<br>92b→ 99b : 0.114533<br>93b→101b : 0.022539<br>95b→ 98b : 0.038486<br>97b→102b : 0.119338
$Q_0 \rightarrow Q_{35}$	5.730	216.4	0.26055	99a→102a : 0.067557<br>99a→104a : 0.028626<br>99a→107a : 0.026165<br>100a→101a : 0.085533<br>100a→106a : 0.063550<br>92b→ 98b : 0.064114<br>92b→100b : 0.029642<br>93b→102b : 0.020578<br>96b→104b : 0.026900<br>97b→102b : 0.186599<br>97b→105b : 0.034419
$Q_0 \rightarrow Q_{36}$	5.748	215.7	0.00642	99a→102a : 0.035601<br>100a→101a : 0.032304<br>85b→ 98b : 0.090422<br>88b→ 98b : 0.026979<br>88b→ 99b : 0.130523<br>88b→101b : 0.101834<br>91b→100b : 0.116404<br>92b→ 98b : 0.038197<br>93b→ 98b : 0.020269<br>93b→101b : 0.027616
$Q_0 \rightarrow Q_{37}$	5.776	214.6	0.00016	97a→104a : 0.029454<br>99a→101a : 0.020345<br>100a→101a : 0.021017<br>85b→ 99b : 0.038665<br>88b→ 99b : 0.114285<br>88b→101b : 0.070037<br>91b→ 99b : 0.030193<br>92b→ 99b : 0.023780<br>93b→ 99b : 0.041819<br>93b→101b : 0.032625<br>94b→102b : 0.024356

				94b→104b : 0.022518<br>95b→100b : 0.023100<br>95b→104b : 0.021889
$Q_0 \rightarrow Q_{38}$	5.810	213.4	0.00380	96a→104a : 0.095970<br>97a→103a : 0.130564<br>98a→104a : 0.038050<br>94b→ 98b : 0.028075<br>94b→100b : 0.021796<br>94b→101b : 0.068810<br>94b→104b : 0.098625<br>95b→102b : 0.082994<br>95b→103b : 0.081930
$Q_0 \rightarrow Q_{39}$	5.829	212.7	0.00320	96a→103a : 0.027872<br>97a→104a : 0.038890<br>88b→ 98b : 0.025884<br>88b→100b : 0.101964<br>88b→101b : 0.022901<br>91b→ 99b : 0.075179<br>93b→ 98b : 0.020480<br>93b→101b : 0.027450<br>94b→102b : 0.033675<br>95b→ 98b : 0.025918<br>95b→103b : 0.021826<br>95b→104b : 0.030042<br>96b→ 99b : 0.057039<br>97b→100b : 0.032117
$Q_0 \rightarrow Q_{40}$	5.841	212.3	0.00079	96a→101a : 0.029408<br>96a→103a : 0.033607<br>97a→104a : 0.038779<br>99a→101a : 0.074729<br>100a→102a : 0.082559<br>88b→100b : 0.051254<br>91b→ 99b : 0.040295<br>91b→100b : 0.026867<br>92b→ 99b : 0.054419<br>94b→102b : 0.023693<br>94b→103b : 0.025552<br>95b→101b : 0.036256<br>95b→103b : 0.021049<br>96b→ 99b : 0.027127
$Q_0 \rightarrow Q_{41}$	5.908	209.9	0.06260	92a→101a : 0.023297<br>95a→102a : 0.023593<br>96a→101a : 0.063938<br>99a→101a : 0.104092<br>99a→106a : 0.021531<br>100a→102a : 0.082818<br>88b→101b : 0.059337<br>91b→102b : 0.052282<br>93b→101b : 0.023366<br>96b→102b : 0.170858

				96b→105b : 0.031505
$Q_0 \to Q_{42}$	5.945	208.6	0.05227	98a→101a : 0.023975<br>99a→101a : 0.073874<br>99a→106a : 0.024862<br>100a→102a : 0.139489<br>100a→107a : 0.030912<br>93b→ 99b : 0.021325<br>95b→ 98b : 0.046831<br>96b→ 99b : 0.030699<br>96b→102b : 0.060477<br>96b→105b : 0.070346<br>97b→104b : 0.025002
$Q_0 \to Q_{43}$	5.956	208.2	0.04837	92a→102a : 0.025738<br>95a→101a : 0.043440<br>96a→102a : 0.059668<br>98a→102a : 0.035467<br>99a→102a : 0.061315<br>100a→101a : 0.114048<br>100a→103a : 0.026454<br>85b→ 98b : 0.020898<br>92b→ 98b : 0.055428<br>92b→100b : 0.030786<br>93b→ 99b : 0.047980<br>94b→ 98b : 0.033405<br>97b→103b : 0.027137
$Q_0 \to Q_{44}$	6.021	205.9	0.15937	99a→102a : 0.048258<br>99a→107a : 0.027485<br>100a→101a : 0.032550<br>100a→103a : 0.031765<br>100a→106a : 0.032930<br>85b→ 98b : 0.063288<br>85b→ 99b : 0.021054<br>91b→100b : 0.030443<br>94b→100b : 0.029369<br>96b→ 98b : 0.029780<br>96b→104b : 0.072100<br>97b→103b : 0.081259
$Q_0 \to Q_{45}$	6.105	203.1	0.03295	99a→103a : 0.042735<br>99a→106a : 0.048248<br>100a→102a : 0.033631<br>100a→104a : 0.065339<br>100a→107a : 0.054393<br>85b→ 98b : 0.024548<br>93b→ 98b : 0.041896<br>93b→100b : 0.037106<br>96b→ 98b : 0.042723<br>96b→ 99b : 0.033019<br>96b→104b : 0.028841<br>97b→ 98b : 0.035858<br>97b→100b : 0.028464

				97b→104b : 0.029234
$Q_0 \rightarrow Q_{46}$	6.106	203.1	0.10527	98a→102a : 0.027195<br>99a→102a : 0.075008<br>99a→103a : 0.026731<br>99a→104a : 0.085764<br>99a→107a : 0.045955<br>100a→101a : 0.020694<br>100a→103a : 0.052587<br>100a→106a : 0.045834<br>96b→103b : 0.046785<br>97b→102b : 0.026192<br>97b→103b : 0.097055<br>97b→105b : 0.067295
$Q_0 \rightarrow Q_{47}$	6.122	202.5	0.03379	99a→103a : 0.038622<br>100a→104a : 0.048150<br>85b→ 98b : 0.022047<br>92b→ 98b : 0.025724<br>92b→ 99b : 0.064056<br>92b→100b : 0.026033<br>93b→ 98b : 0.024671<br>93b→100b : 0.026052<br>95b→ 98b : 0.053494<br>96b→ 98b : 0.050304<br>96b→100b : 0.070764<br>96b→103b : 0.027285<br>97b→ 98b : 0.053823<br>97b→ 99b : 0.063704<br>97b→104b : 0.079516
$Q_0 \rightarrow Q_{48}$	6.145	201.8	0.03167	97a→104a : 0.020809<br>85b→ 98b : 0.027226<br>92b→ 98b : 0.031169<br>92b→ 99b : 0.040770<br>93b→ 99b : 0.062777<br>93b→100b : 0.026660<br>95b→ 98b : 0.061565<br>95b→ 99b : 0.026147<br>95b→100b : 0.061983<br>96b→ 99b : 0.092804<br>96b→100b : 0.045632<br>96b→103b : 0.026312<br>97b→ 99b : 0.126462<br>97b→104b : 0.032322

Table S7. Molar extinction coefficient (ε) of the Co(II) complexes at their maximum absorption wavelengths and of the LEDs used in the photopolymerization experiments (365 nm, 405 nm, and 420 nm).

Complex	λ_{1max}(nm)	λ_{2max}(nm)	ε_1 (mol/ L cm^{-1})	ε_2 (mol/ L cm^{-1})	ε_{365nm} (mol/L cm^{-1})	ε_{405nm} (mol/L cm^{-1})	ε_{420nm} (mol/L cm^{-1})
Co-Ph	362	423	23500	19800	23200	15900	19700
Co-EtO	335	388	22600	18700	19300	17800	15000
Co-Cl	354	415	11300	13000	10700	11100	12300
Co-Me	355	413	7500	8400	7000	7400	7400
Co-*t*Bu	351	414	9300	9500	8200	8200	9000

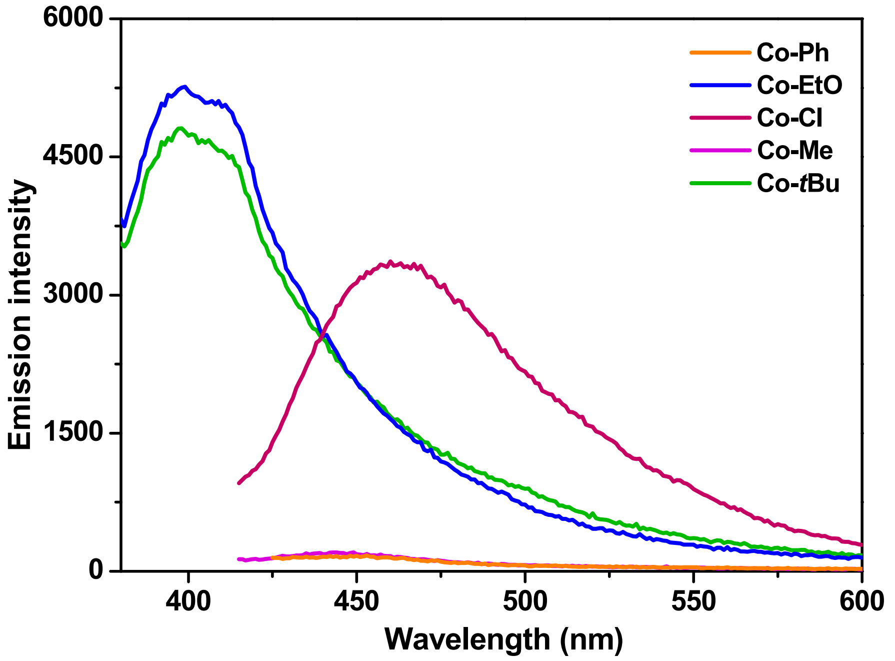

Figure S27. Emission spectra of the Complexes **Co-Ph**, **Co-EtO**, **Co-Cl**, **Co-Me** and **Co-*t*Bu** in CH_2Cl_2 ([Co] = 1 × 10^{-5} mol L^{-1}) at 25 °C; excitation was performed at 365.

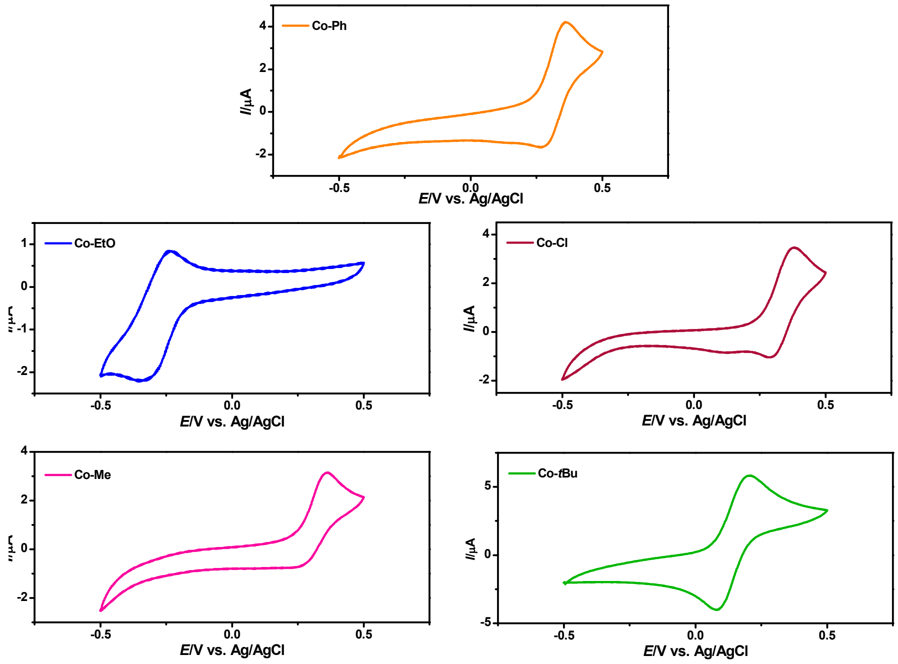

Figure S28. Cyclic voltammetry of the **Co-Ph**, **Co-EtO**, **Co-Cl**, **Co-Me**, and **Co-*t*Bu** complexes in DMF ([Co] = 1 × 10^{-3} mol L^{-1}; [n-Bu_4NPF_6] = 0.10 mol L^{-1}) at 25 °C, with a scan rate of 100 mV s^{-1} and a potential window from −0.5 to 0.5 V.

Table S8. Cyclic voltammetry results for Co(II) complexes

Complex	E_{ap} (V)	E_{cp}(V)	$E_{1/2}^{b}$(V)	ΔE_p^{c} (V)
Co-Ph	0.36	0.27	0.32	0.09
Co-EtO	−0.24	−0.34	−0.29	0.10
Co-Cl	0.38	0.29	0.34	0.09
Co-Me	0.35	0.25	0.30	0.10
Co-*t*Bu	0.20	0.08	0.14	0.12

[a] Conditions: DMF, *n*-Bu_4NPF_6 (supporting electrolyte, 0.1 mol L^{-1}), [Co] = 1 mmol L^{-1} (scan rate = 100 mV s^{-1}), platinum disk and wire (working and auxiliary electrodes), Ag/AgCl in saturated KCl (reference electrode); [b] $E_{1/2}$ is the half-wave potential for the complex; [c] ΔE_p is the cathodic-anodic peak separation.

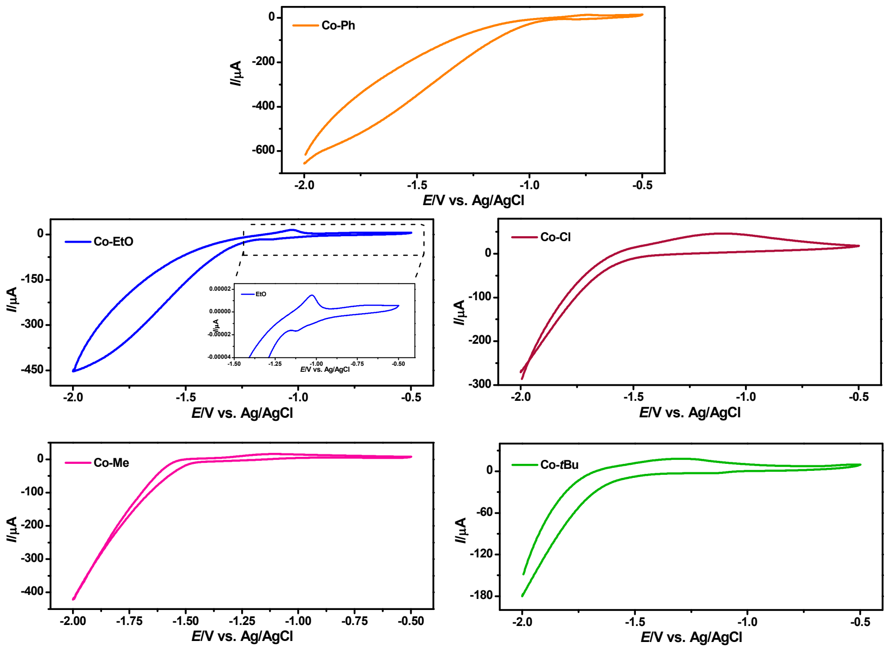

Figure S29. Cyclic voltammetry of the **Co-Ph**, **Co-EtO**, **Co-Cl**, **Co-Me**, and **Co-*t*Bu** complexes in DMF ([Co] = 1×10^{-3} mol L^{-1}; [n-Bu_4NPF_6] = 0.10 mol L^{-1}) at 25 °C, with a scan rate of 100 mV s^{-1} and a potential window from −2.0 to −0.5 V.

Table S9. Cyclic voltammetry results for Co(II) complexes

Complex	E_{ap} (V)	E_{cp}(V)	$E_{1/2}$[b](V)	ΔE_p[c] (V)
Co-Ph	−0.75	−0,80	−0.78	0.05
Co-EtO	−1.03	−1.13	−1.08	0.10
Co-Cl	−1.07	--	--	--
Co-Me	−1.11	−1,24	−1.18	0.13
Co-*t*Bu	−1.27	−1.19	−1.23	0.08

[a] Conditions: DMF, *n*-Bu_4NPF_6 (supporting electrolyte, 0.1 mol L^{-1}), [Co] = 1 mmol L^{-1} (scan rate = 100 mV s^{-1}), platinum disk and wire (working and auxiliary electrodes), Ag/AgCl in saturated KCl (reference electrode); [b] $E_{1/2}$ is the half-wave potential for the complex; [c] ΔE_p is the cathodic-anodic peak separation.

Table S10. Excited state energies E^*, oxidation potentials E_{ox}, reduction potentials E_{red}, and free energy change (ΔG_{et}) for the PC/Iod and PC/EDB interactions.

	Co-Ph	Co-EtO	Co-Cl	Co-Me	Co-*t*Bu
λ_{E*}(nm)	450	445	440	443	445
$^{a}E_{S1}*$ (eV)	2.75	2.79	2.82	2.80	2.79
$^{b}E_{ox}$ vs. SCE* (V)	0.28	−0.35	0.27	0.27	0.09
$^{c}E_{red}$ vs. SCE* (V)	---	−1.00	---	---	---
$^{d}\Delta G^{S1}_{et}$ (eV)^{Iod}	−2.27	−2.94	−2.35	−2.33	−2.50
$^{e}\Delta G^{S2}_{et}$ (eV)^{EDB}	---	−0.79	---	---	---

[a] Non-luminescent; the excited state energy level is obtained from the edge of the UV-Vis absorption spectrum.

[b] The oxidation potential was determined from the onset of the cyclic voltammogram.

[c] The reduction potential was determined from the onset of the cyclic voltammogram.

[d] The reduction potential Iod is –0.20 V.[1]

[e] The oxidation potential EDB is 1.00 V.[2].

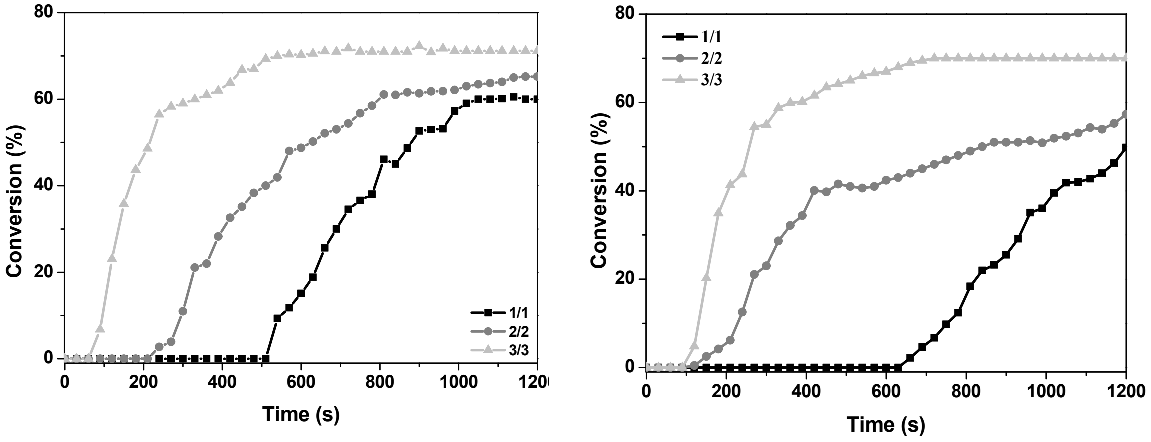

Figure S30. Conversion of TMPETA and irradiation time in laminate, using different photocatalysts with 1%/1%, 2%/2% and 3%/3% w/w/w for Iod/EDB. **(a)** LED@365 nm, **(b)** LED@390-405 nm.

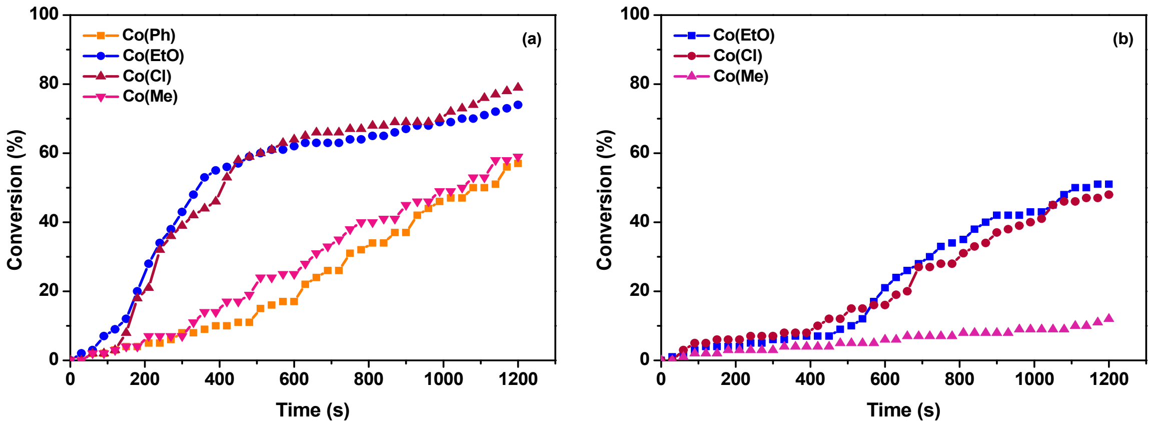

Figure S31. Conversion of TMPETA and irradiation time in laminate, using different photocatalysts with 0.1%/1%/1% w/w/w for Co^{II}/Iod/EDB. **(a)** LED@365 nm, **(b)** LED@390-405 nm.

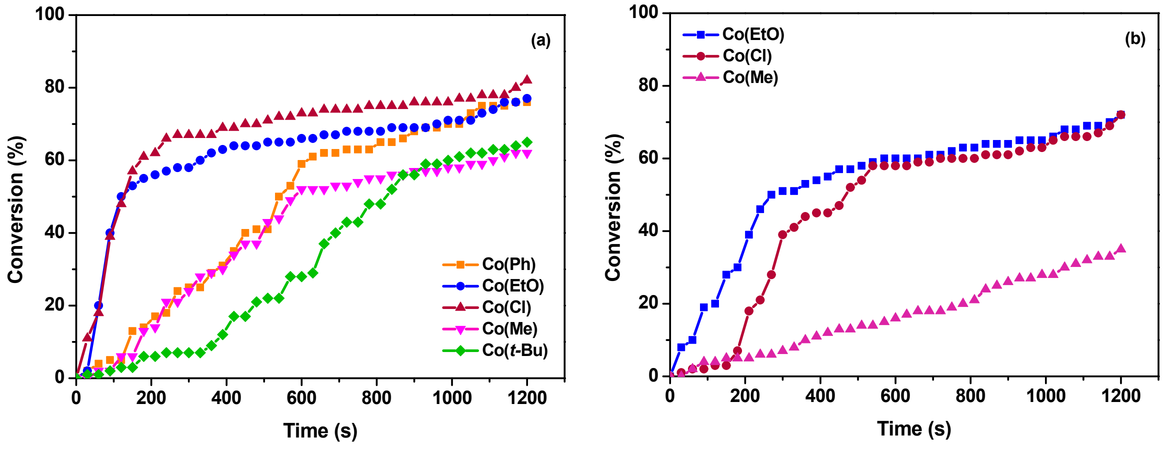

Figure S32. Conversion of TMPETA and irradiation time in laminate, using different photocatalysts with 0.1%/2%/2% w/w/w for Co^{II}/Iod/EDB. **(a)** LED@365 nm, **(b)** LED@390-405 nm.

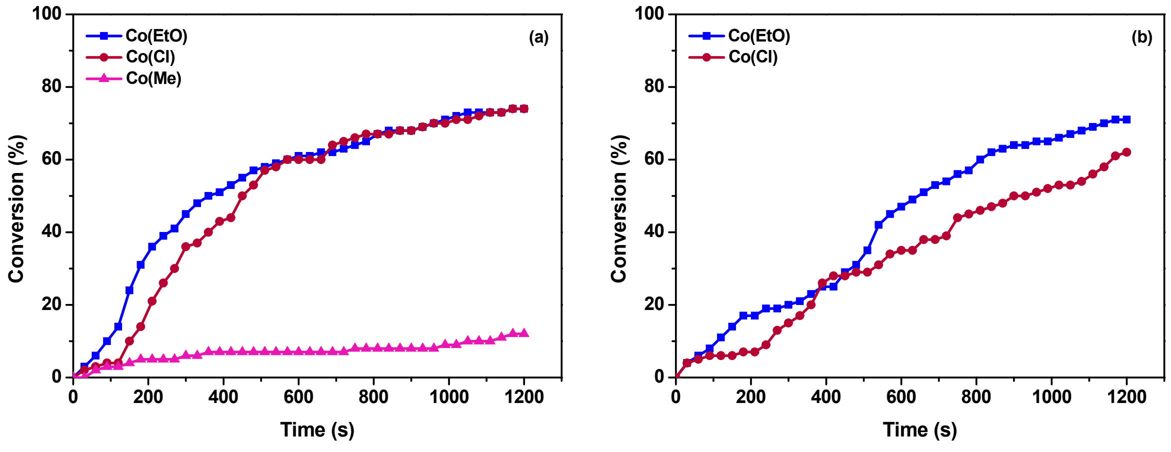

Figure S33. Conversion of TMPETA and irradiation time in laminate, using different photocatalysts with 0.2%/1%/1% w/w/w for Co^{II}/Iod/EDB. **(a)** LED@365 nm, **(b)** LED@390-405 nm.

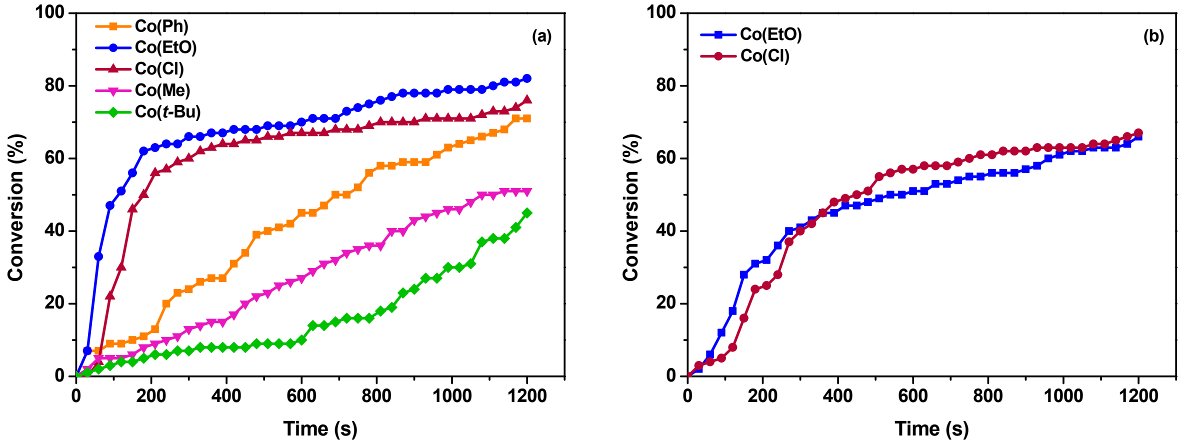

Figure S34. Conversion of TMPETA and irradiation time in laminate, using different photocatalysts with 0.2%/2%/2% w/w/w for Co^{II}/Iod/EDB. **(a)** LED@365 nm, **(b)** LED@390-405 nm.

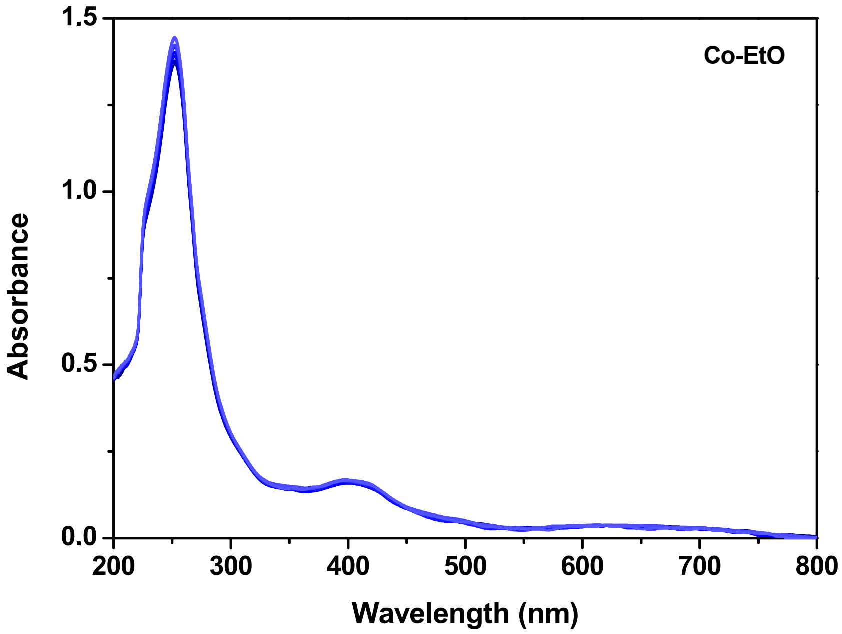

Figure S35. UV-Vis absorption spectra of **Co-EtO** in CH_2Cl_2 under LED@365 nm for 150 minutes.

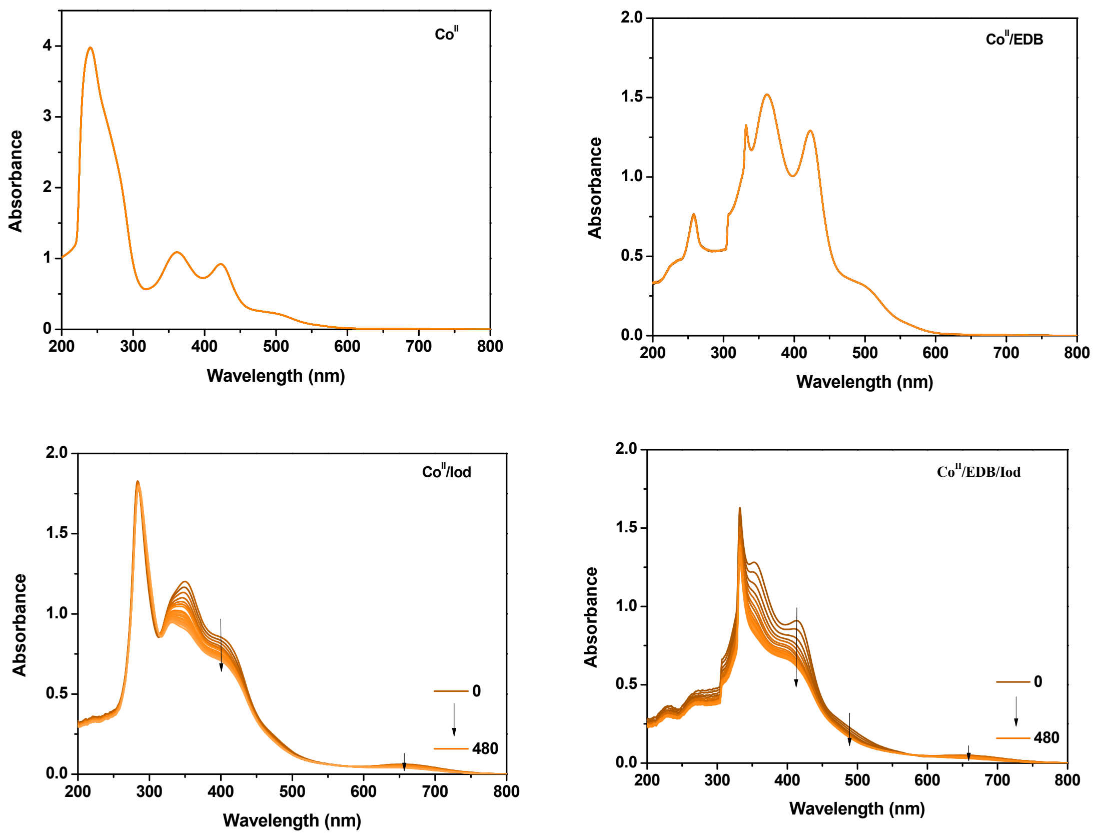

Figure S36. UV-Vis absorption spectra of **Co-Ph** in CH_2Cl_2 under LED@365 nm for different times; (a) **Co-Ph**, (b) **Co-Ph**/EDB, (c) **Co-Ph**/Iod and (d) **Co-Ph**/EDB/Iod.

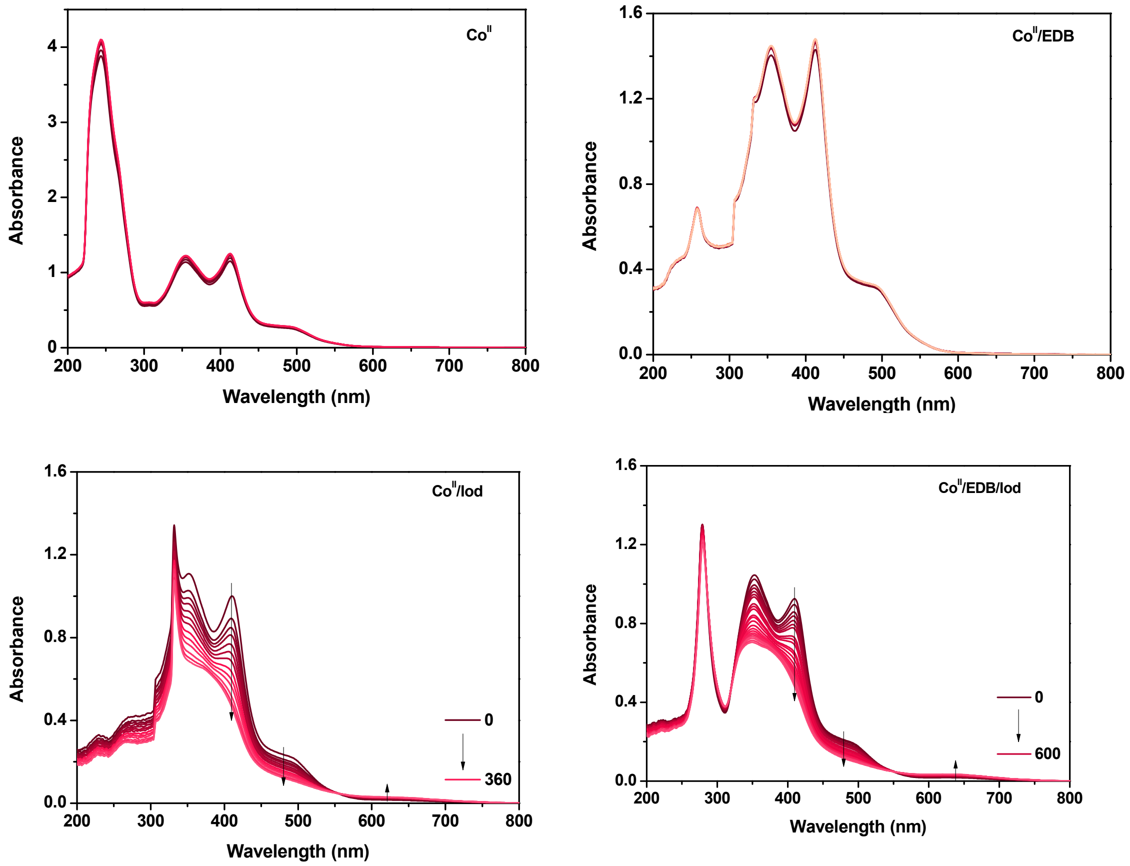

Figure S37. UV-Vis absorption spectra of **Co-Cl** in CH_2Cl_2 under LED@365 nm for different times; (a) **Co-Cl**, (b) **Co-Cl**/EDB, (c) **Co-Cl**/Iod and (d) **Co-Cl**/EDB/Iod.

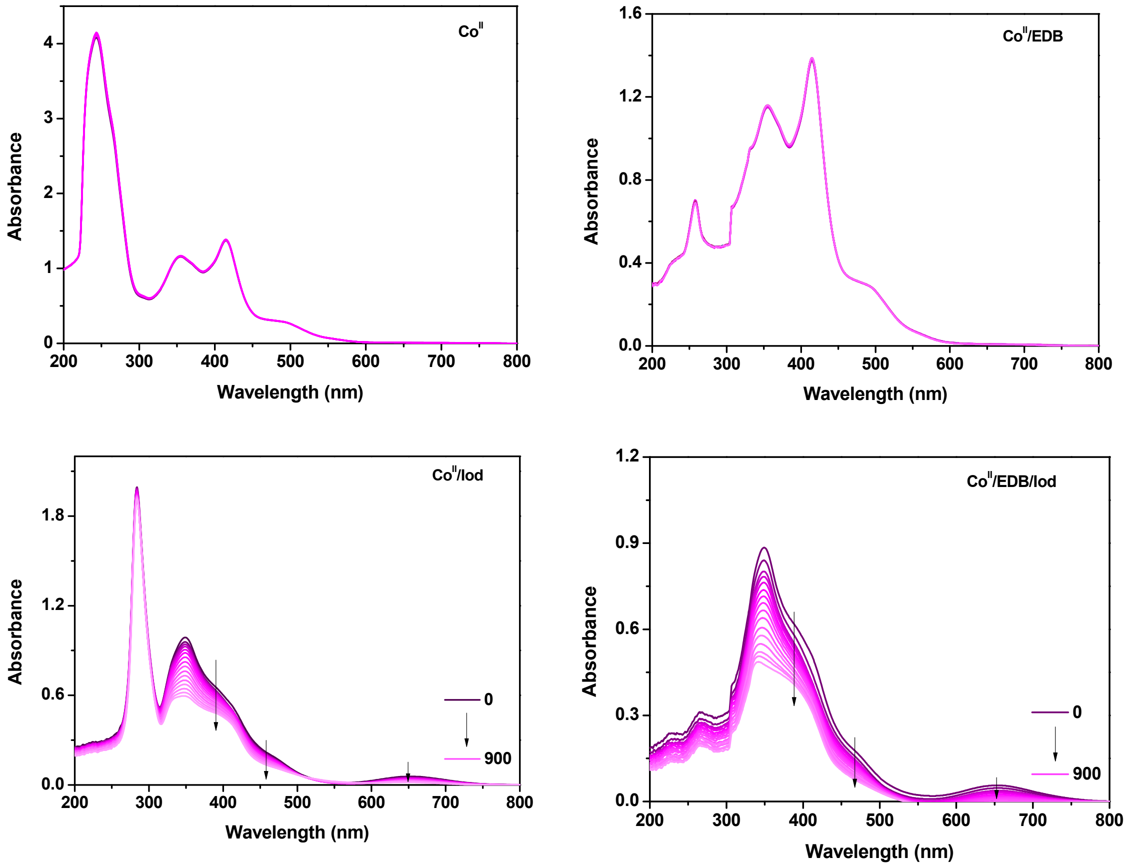

Figure S38. UV-Vis absorption spectra of **Co-Me** in CH_2Cl_2 under LED@365 nm for different times; (a) **Co-Me**, (b) **Co-Me**/EDB, (c) **Co-Me**/Iod and (d) **Co-Me**/EDB/Iod.

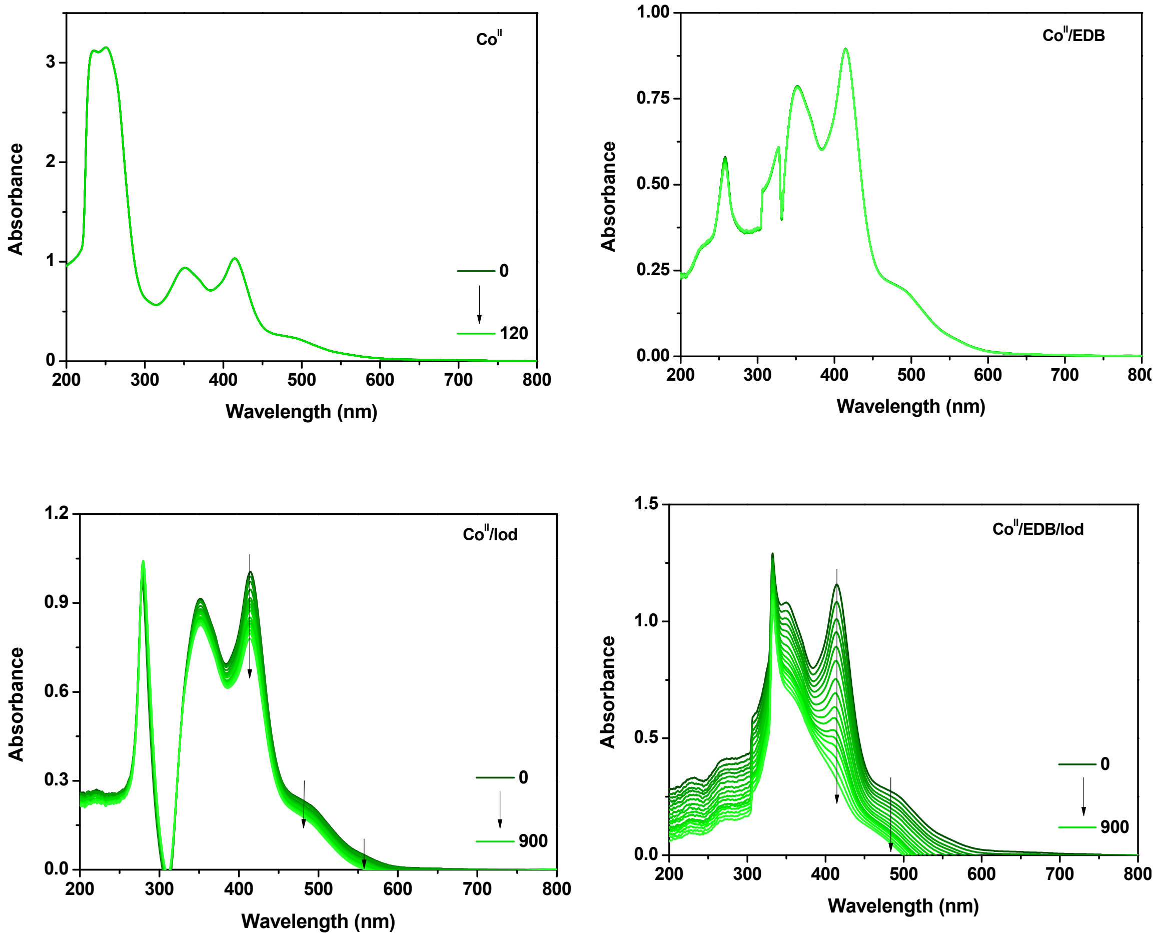

Figure S39. UV-Vis absorption spectra of **Co-*t*Bu)** in CH_2Cl_2 under LED@365 nm for different times; (a) **Co-*t*Bu)**, (b) **Co-*t*Bu**/EDB, (c) **Co-*t*Bu**/Iod and (d) **Co-*t*Bu**/EDB/Iod.

References

(1) Al Mousawi, A.; Kermagoret, A.; Versace, D. L.; Toufaily, J.; Hamieh, T.; Graff, B.; Dumur, F.; Gigmes, D.; Fouassier, J. P.; Lalevée, J. Copper photoredox catalysts for polymerization upon near UV or visible light: Structure/reactivity/efficiency relationships and use in LED projector 3D printing resins, *Polym Chem* **2017**, *8* (3) 568–580. https://doi.org/10.1039/C6PY01958G.

(2) Pesqueira, N. M.; Morlet-Savary, F.; Schmitt, M.; Carvalho-Jr, V. P.; Goi, B. E.; Lalevée, J. Advancing Photopolymerization and 3D Printing: High-Performance NiII Complexes Bearing N2O2 Schiff-Base Ligands as Photocatalysts. *Eur. Polym. J.* **2024**, 216 (113279), 113279. https://doi.org/10.1016/j.eurpolymj.2024.113279.
